# A Systematic Review of the Efficacy of Interventions to Control Aflatoxins in the Dairy Production Chain—Feed Production and Animal Feeding Interventions

**DOI:** 10.3390/toxins14020115

**Published:** 2022-02-03

**Authors:** Zsuzsa Farkas, Erika Országh, Tekla Engelhardt, Szilveszter Csorba, Kata Kerekes, Andrea Zentai, Miklós Süth, Attila Nagy, Gabriella Miklós, Krisztina Molnár, Csaba Rácz, Tamás Dövényi-Nagy, Árpád Ambrus, Zoltán Győri, Attila Csaba Dobos, Tünde Pusztahelyi, István Pócsi, Ákos Jóźwiak

**Affiliations:** 1 Digital Food Institute, University of Veterinary Medicine Budapest, 1078 Budapest, Hungary; orszagh.erika@univet.hu (E.O.); engelhardt.tekla@univet.hu (T.E.); csorba.szilveszter@univet.hu (S.C.); suth.miklos@univet.hu (M.S.); jozwiak.akos@univet.hu (Á.J.); 2 System Management and Supervision Directorate, National Food Chain Safety Office, 1024 Budapest, Hungary; kerekes.kata@gmail.com (K.K.); a.zentai@gmail.com (A.Z.); 3 Food Chain Safety Laboratory Directorate, National Food Chain Safety Office, 1095 Budapest, Hungary; nagyattila@nebih.gov.hu; 4 Székesfehérvár Regional Food Chain Laboratory, National Food Chain Safety Office, 8000 Székesfehérvár, Hungary; MiklosG@nebih.gov.hu; 5 Centre for Precision Farming R&D Services, Faculty of Agriculture, Food Science and Environmental Management, University of Debrecen, 4032 Debrecen, Hungary; molnark@agr.unideb.hu (K.M.); raczcs@agr.unideb.hu (C.R.); dovenyi@agr.unideb.hu (T.D.-N.); dobosa@agr.unideb.hu (A.C.D.); 6 Doctoral School of Nutrition and Food Sciences, University of Debrecen, 4032 Debrecen, Hungary; ambrusadr@yahoo.co.uk; 7 Institute of Nutrition, Faculty of Agricultural, Food and Environmental Management of University of Debrecen, 4032 Debrecen, Hungary; gyori.zoltan@unideb.hu; 8 Central Laboratory of Agricultural and Food Products, Faculty of Agricultural and Food Sciences and Environmental Management, University of Debrecen, 4032 Debrecen, Hungary; pusztahelyi@agr.unideb.hu; 9 Department of Molecular Biotechnology and Microbiology, Institute of Biotechnology, Faculty of Science and Technology, University of Debrecen, 4032 Debrecen, Hungary; pocsi.istvan@science.unideb.hu

**Keywords:** aflatoxin, control strategies, aflatoxin mitigation, feed and farm interventions, dairy production chain, maize contamination

## Abstract

The study presents a systematic review of published scientific articles investigating the effects of interventions aiming at aflatoxin reduction at the feed production and animal feeding phases of the milk value chain in order to identify the recent scientific trends and summarize the main findings available in the literature. The review strategy was designed based on the guidance of the systematic review and knowledge synthesis methodology that is applicable in the field of food safety. The Web of Science and EBSCOhost online databases were searched with predefined algorithms. After title and abstract relevance screening and relevance confirmation with full-text screening, 67 studies remained for data extraction, which were included in the review. The most important identified groups of interventions based on their mode of action and place in the technological process are as follows: low-moisture production using preservatives, acidity regulators, adsorbents and various microbiological additives. The results of the listed publications are summarized and compared for all the identified intervention groups. The paper aimed to help feed producers, farmers and relevant stakeholders to get an overview of the most suitable aflatoxin mitigation options, which is extremely important in the near future as climate change will likely be accompanied by elevated mycotoxin levels.

## 1. Introduction

Aflatoxins are a group of toxic secondary metabolites produced by certain filamentous fungi (moulds), which infect important staple crops, predominantly with *Aspergillus flavus*, *A. parasiticus* and *A. nomius* [1,2]. The most important types of aflatoxins are B1, B2, G1, G2, M1 and M2 [3].

Aflatoxins have been found in major food crops, notably nuts, grains and their derived products, which may become contaminated both before and after harvesting [4]. Contaminated food crops pose serious economic and health challenges. Aflatoxins are carcinogenic and immunosuppressive compounds and may affect all organs, especially the liver and the kidneys. Aflatoxin B1 (AFB1) is carcinogenic to humans, and the International Agency for Research on Cancer (IARC) classifies aflatoxins as Group 1—carcinogenic to humans [5].

Acute intake of aflatoxins in high amounts may cause serious poisoning (aflatoxicosis), which can be life-threatening, usually through damage to the liver. Several outbreaks of aflatoxicosis have been observed in animals and in human populations since the 1960s [6,7]. Aflatoxins may also lead to health problems in animals; furthermore, the destruction of contaminated crops poses a severe burden on the economy [7]. When fed to livestock animals, e.g., dairy cows, the aflatoxin contamination of feed is metabolized into aflatoxin M1 (AFM1) and appears in the milk. Although less potent than AFB1, it still has toxic and carcinogenic properties, therefore, any amount of AFM1 in milk is undesirable and should be avoided [8,9].

The infection of crops by moulds and their toxin production are dependent on several factors. The growth range of *A. flavus* and *A. parasiticus* takes place over a temperature range of 20–35 °C, with a water activity of >0.90 [10]. Under favourable conditions, they are typically found in tropical and subtropical regions with high temperatures and high humidity. However, the occurrence of aflatoxins has become usual in more temperate regions like Central Europe, too [11,12,13,14,15]. Research indicates that aflatoxin production can be observed at the marginal growth conditions [10,16]. Drought stress, insect damage and poor hygienic conditions during transportation and storage also contribute to contamination.

Several factors have been investigated in connection to aflatoxin contamination of the milk value chain. A promising direction to address the challenge may be the breeding of crops that are resistant to mycotoxins. Another direction is the utilisation of nontoxigenic *Aspergillus* strains outcompeting the toxigenic strains and thereby limiting toxin production. There are also different postharvest practices (cleaning, sorting, chemical treatments, etc.) to eliminate the contamination or mitigate the adverse effects in animals [7].

This study presents a systematic review of the published articles investigating any effects on the aflatoxin content of corn and subsequently in cow milk. The final objective was to identify the critical points in the feed production, processing and animal feeding parts of the milk value chain where aflatoxin contamination can be effectively controlled.

## 2. Materials and Methods

### 2.1. Review Approach

The review strategy was designed based on the guidance of the systematic review and knowledge synthesis methodology that is applicable in the field of food safety [17,18,19]. The basic systematic review methodology was adjusted to be fit for purpose, e.g., the search was targeted, detailed analyses were prioritized, and only one reviewer conducted the relevance confirmation and data extraction steps. Meta-analysis was not performed. The PRISMA 2020 checklist [20] can be seen in Appendix E.

The primary data source was peer-reviewed scientific articles, risk assessments, as well as primary research. The intention was to provide results with data from the findings of the publications (can be seen in the Supplementary Materials), therefore, reviews containing no data were not considered.

### 2.2. Review Team

The core review team consisted of ten individuals with relevant (agriculture, food safety, microbiology and veterinary public health) and methodological (knowledge synthesis) expertise. The method-related activities were implemented and executed by the core team members who met regularly throughout the review procedure. Prior to implementing the review, the review protocol, the proposed approach and the inclusion and exclusion criteria during the screening and selection of relevant articles were shared with members of the group for feedback.

### 2.3. Review Question, Scope and Eligibility Criteria

The key review question was: What is the effect of targeted feed production, animal feeding or animal husbandry interventions on aflatoxin levels of corn, feed, milk and milk products from feed processing to milk production? It was framed by using the PICO (population, intervention, comparison, outcome) process which is widely used in systematic human health reviews and had been adapted to our purposes.

The population of interest included corn produced for animal (cattle and other ruminants) feeding purposes. Interventions comparing the effect with other interventions or with situations where no interventions were implemented were sought. The main examined outcome was the aflatoxin level in corn-based feed, aflatoxin levels in animals and milk, as well as animal health, status and zootechnical parameters. This paper focused on the interventions applicable at the feed production and animal feeding intervention phases of the milk processing chain.

### 2.4. Search Strategy

Studies that evaluated the effect of agricultural interventions on aflatoxin levels of corn and in animals were identified by searching the Web of Science (https://www.webofscience.com (accessed on 2 January 2022)) and EBSCOhost (https://www.ebsco.com (accessed on 2 January 2022)) online databases. The keyword selections comprised combinations of terms related to the targeted population of interest and intervention types, hence, the following general form was used:

[Keywords regarding the fungal infections which affect the aflatoxin levels of corn (e.g., aflatoxin OR *Aspergillus*); all separated by OR] AND [types of the population of interest (e.g., milk OR corn); all separated by OR] AND [types of intervention (e.g., preservation OR rodent control); all separated by OR]. Searches were run in the publication titles, abstracts and keywords and were restricted to only those studies which were published in English from 2013 to November 2019. More details on the search algorithms are reported in Appendix A. A limitation of the review process was that publications that cover the topic but are written in a language other than English are not indexed by EBSCOhost or Web of Science; the studies that did not meet the inclusion/exclusion criteria or did not fall into the defined timeframe were excluded from the review.

### 2.5. Title and Abstract Relevance Screening (AS)

Abstract screening was performed based on relevance regarding the research question in the Zotero web application (https://www.zotero.org (accessed on 2 January 2022)). Abstract selection was performed by two independent reviewers. In case of conflicting opinions regarding the exclusion or inclusion of a publication based on the content of the abstract, the decision was made by an independent supervisor. As the two domains investigated (storage and feed; farm) had many articles in common, the duplicates between the domains were also excluded. The inclusion and exclusion criteria for the title and abstract relevance screening are summarized in Appendix B.

### 2.6. Relevance Confirmation during Full Text Screening

The papers selected during the title and abstract relevance screening were accessed as full-text articles and relevance confirmation was performed with the help of a predefined form (Appendix C) by single reviewers. During this phase, the papers which did not investigate the effect of interventions on aflatoxins in maize, the papers not in English and the papers that did not contain data on the magnitude of the effect of interventions on aflatoxins available for extraction were excluded. The papers passing this stage were assessed in detail and data were extracted from them in a subsequent step. The keywords were aimed at searching for cattle as the target population; however, if relevant information was found with results for other ruminants, they were not excluded during relevance screening.

### 2.7. Data Extraction

As the main objective of the study was to summarize the effect of various interventions on aflatoxins, data providing evidence on intervention effectiveness were extracted from the selected papers. The extraction was performed by a single reviewer with the help of a data extraction form (Appendix D). The form included data fields on paper identification (authors, title, publication details), on the main characteristics of the study (point in the food chain, intervention category), on intervention details (intervention description, target population/sample, outcome measured, description of the outcome and the data extracted from the outcomes) and on the study quality indicators (study design, number (magnitude) of samples, level of data reported, dose–response gradient, region of the study). Study quality indicators were used to prioritize outcomes in cases when conflicting outcomes were present throughout the search corpus.

## 3. Results

### 3.1. Results of the Review Process

**Figure 1:**
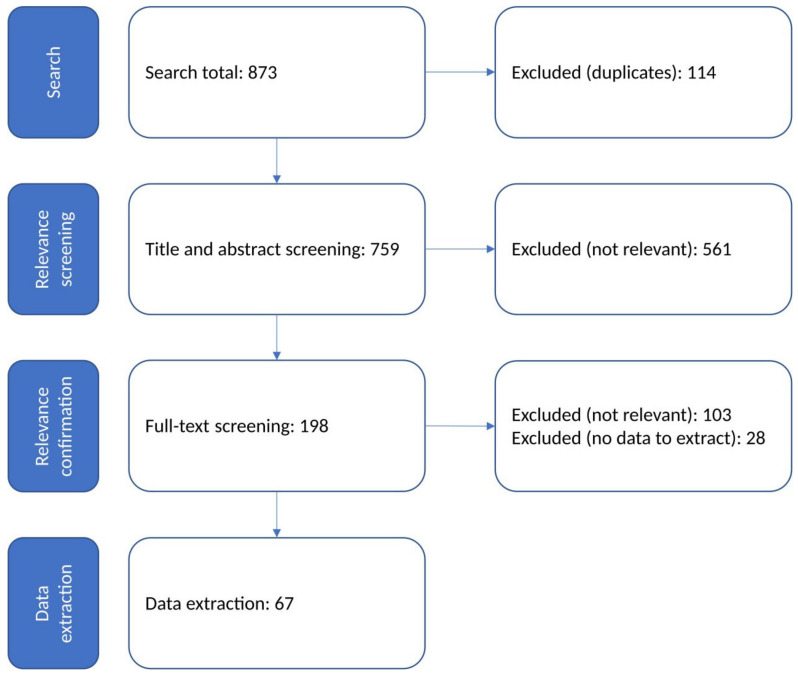
Systematic review knowledge synthesis process flow diagram.

A process flow diagram of the knowledge synthesis process for the review is shown in Figure 1.

The 67 papers which were subject for detailed assessment and data extraction contained 126 different interventions. The key characteristics of the 126 relevant interventions are shown in Table 1.

### 3.2. Summary of the Key Findings Regarding Feed Production and Animal Feeding Interventions

The risk reduction interventions regarding aflatoxins during feed production and animal feeding encompass a multitude of various methods. The most important identified groups of interventions based on their mode of action and place in the technological process are the following: low-moisture production using preservatives, acidity regulators, adsorbents and various microbiological additives. A summary of the key findings is presented here. The detailed extracted data and information related to the intervention groups and individual interventions are presented in the Supplementary Materials.

#### 3.2.1. Low-Moisture Production

Aflatoxins are heat-stable molecules and cannot be eliminated using different heat treatments, therefore aflatoxin formation prevention is essential. The rapid drying of agricultural products to reduce their moisture content is an important method, which prevents the formation of favourable conditions for the growth of fungi.

Chiewchan et al. (2015) [21] reviewed the possibilities of application of different drying methods to control mould growth and aflatoxin production. The first method was drying sheep feed composed of crushed maize, wheat bran and peanut meal as a thin layer using a hot air oven at 80 °C for 6 h. This technology resulted in a 57.6% reduction of aflatoxins. The second method, drying under sunlight at ambient temperature for 2 days, resulted in an 83.7% reduction of aflatoxins. However, in other studies, drying whole maize kernels at 40–70 °C presented no eradication efficacy regarding aflatoxins.

#### 3.2.2. Preservatives

Testing of naturally occurring antimicrobials for the preservation of food and feed products has been receiving increasing attention due to the growing concern of microbial resistance towards conventional preservatives. The safety of chemical preservatives generated a strong debate since they are considered responsible for many carcinogenic and teratogenic attributes as well as residual toxicity.

Koc and Kara (2014) [22] investigated the antifungal potential of thyme, laurel and rosemary essential oils against *A. flavus* and *A. parasiticus* in order to use them as an alternative to preservatives such as potassium sorbate.

Each essential oil—especially at higher concentrations—showed antifungal activities against *A. parasiticus* and *A. flavus*. The storage time had no significant effect on the antifungal activity. The most promising essential oil was thyme oil, which featured the highest inhibition of mould growth at all concentrations, followed by the preservative, potassium sorbate, then rosemary and laurel oils.

In another study, conducted by Garcia et al. (2012) [23], the antifungal capacity of the *Equisetum arvense* extract was tested against *A. flavus*. The inhibitory effect of the extract was only observed in the inoculated treatments regardless of the incubation time, with a 45% population reduction.

Ashgar et al. (2018) [24] investigated the antifungal activity of and reduction of aflatoxin production by iron (Fe), copper (Cu) and silver (Ag) nanoparticles (NPs) extracted from green tea and black tea leaves. Ag NPs showed the highest antifungal activity and aflatoxin reduction in comparison to Fe NPs and Cu NPs.

The study also examined the effect of NPs on AFB1 adsorption activity in different conditions. The adsorption activities of the metal NPs followed the order of Fe NPs > Cu NPs > Ag NPs, but they were not significantly different from each other at *p* ≤ 0.05. The study concluded that metal NPs may be utilized as a possible aflatoxin adsorbent in human food and animal feed such as rice, wheat, maize, red chili peppers and poultry feed.

The effectiveness of four additives was tested by Shi et al. (2017) [25] in distillers wet grains (DWG) and condensed distillers solubles (CDS), namely sodium bisulfite, sodium hypochlorite, citric acid and ammonium persulfate. According to the results, sodium bisulfite was not highly efficient in degrading aflatoxins neither in DWG (24% reduction) nor in CDS (35%). Among the four additives tested, sodium hypochlorite was the most effective (42% reduction in DWG and 56% in CDS), but it bleached the substrate and left an off odour, therefore the authors concluded that sodium hypochlorite is not suitable for aflatoxin degradation in food and feed products. Citric acid and ammonium persulfate reduced aflatoxin levels by 31–51% and the effect of citric acid could be further enhanced by increasing the addition level and prolonging the heating time.

#### 3.2.3. Acidity Regulators

Organic acids—similarly to some nutrients like selenium [26]—are natural preservatives and antioxidants and are present in feed as common constituents or could be added artificially to enhance their flavour. They are also suitable for aiding the degradation of AFB1 in food.

Aiko et al. (2016) [27] investigated the effect of three different organic acids on the degradation of AFB1 at high temperatures. The results showed that among acetic acid, citric acid and lactic acid, the latter was the most efficient in degrading AFB1, and the efficacy increased with concentration, heating temperature and duration. The most effective degradation of AFB1 was observed at the 1 mol L^−1^ concentration of lactic acid when heated for 60 min to 80 °C.

Singh and Mandal (2014) [28] studied the efficacy of fumaric and citric acids in preventing the biosynthesis of aflatoxins in poultry feed. The results showed that at the 11% moisture level, none of the studied aflatoxins were recorded in any of the treatments, but with the increase in moisture in feed, the production of aflatoxins also increased. It was concluded that storage of feed for 1 month with 13% moisture content is only safe if the production of aflatoxins is inhibited by adding fumaric acid or citric acid at the concentration of 0.2% and 0.45%, respectively.

Propionic acid is a very effective and cheap mould inhibitor; however, it has an unpleasant odour and corrosive nature, which hinder its use in food and feed products. This problem can be solved using its salts, sodium and calcium propionates, which have no offensive odour and are not corrosive but have a fungistatic effect [29].

Alam et al. (2014) [30] studied the effects of calcium propionate, water activity (a_w_), and incubation time on the total fungal count and aflatoxins B1 (AFB1), B2 (AFB2), G1 (AFG1) and G2 (AFG2) production in broiler feed. All the factors (preservative, a_w_ and storage time) alone and in combination significantly reduced the total fungal count and aflatoxin production in the feed. All the aflatoxins increased over the storage time; however, the increase was moderate in the preserved feed that contained a lower amount of available water. This study proved that calcium propionate along with decreased water activity can serve as an effective tool for controlling mould incidence and aflatoxin production.

Lee, Her and Lee (2015) [31] observed a high reduction (93–95%) of aflatoxin levels after treating soybean with 1.0 N citric, lactic and tartaric acids for 18 h.

#### 3.2.4. Adsorbents

Decontamination of feed by adding adsorbents to the diet that can bind aflatoxin molecules is a common way of controlling aflatoxin contamination and thereby protecting animal and human health from adverse effects of aflatoxins. Clay-based feed additives are most frequently used for this purpose; charcoal, glucomannan and plant-based products are also used or investigated. The most important questions regarding the adsorbents used as feed additives are their effectiveness and their physiological effects, therefore the examined parameters in the studies presented can be grouped into the following categories (Table 2): in vitro examinations of aflatoxin adsorption/binding capacity, antifungal activity, ruminal fermentation parameters and in vivo examinations of aflatoxin degradation/reduction in animals (aflatoxin levels in blood, urine, faeces, milk), carryover/transfer rate, reduction of aflatoxin excretion, as well as animal health status/zootechnical parameters like blood (plasma) parameters, performance (e.g., milk yield, feed intake, milk composition), general health status and immune status.

This section provides an insight regarding the abovementioned adsorbent types, their combinations and toxin-binding premixes (containing not only technological additives, but other beneficial substances as well) by giving a brief description of the outcomes of the studies in this field, summarized in Table 2. The chapter is divided by considering the adsorbent type and the study type—comparative or single-substance investigation.

In conclusion, almost all the studies presented that the investigated adsorbents or adsorbent-based premixes are efficient regarding aflatoxin adsorption or reduction of aflatoxin levels, transfer rate and excretion of aflatoxins. Most of the results were statistically significant regarding the examined parameters. The exceptions were as follows:

In the study of Pate et al. (2018) [36], there was no change in aflatoxin excretion after adding aluminosilicate clay to the diet, which was explained by increased milk yield and feed efficiency parameters by the authors.

Kissel et al. (2012) [42] found no effects regarding aflatoxin reduction in the case of glucomannan and aluminosilicate blend and modified glucomannan treatment.

Weatherly et al. (2018) [44] found no change in the transfer rate and excretion parameters when adding yeast fractions and bentonite to the diet; however, aflatoxin B1 levels in faeces decreased in the study in a quadratic manner.

In the study of Ogunade et al. (2016) [55], no differences were shown regarding aflatoxin levels, transfer rate and excretion. The feed additives used were *Saccharomyces cerevisiae* fermentation products containing a low or high dose of a chlorophyll-based additive or a low dose of a chlorophyll-based additive and sodium bentonite clay. However, when AFB1 was withdrawn from the diet, AFM1 concentrations decreased rapidly in the treated groups.

There is an unambiguous relationship regarding the interactions between aflatoxins and adsorbents. Some studies investigated the nature of these relationships from various aspects. In the studies of Maki et al. (2016a) [34], and Maki et al. (2016b) [35], a clear clay dose-dependent reduction of aflatoxin concentration was shown. Antonelo et al. (2017) [33] proved a linear toxin dose effect, while Xiong et al. (2015) [50] showed that the adsorbent at high AFB1 concentrations was not effective. Weatherly et al. (2018) [44] found a quadratic decrease in AFB1 reduction by the adsorbent treatment in faeces.

Regarding animal health status and zootechnical parameters, it can be concluded that no negative effects of the adsorbent treatments were shown in any of the studies for any of the examined parameters. The results were mainly neutral—meaning that the feed additives did not have any adverse effects on the animals. Nine studies showed positive effects regarding any of the parameters belonging to this group (Table 2) [37,39,43,44,50,51,53,54,55]; there was significant improvement in the following parameters: blood (plasma) parameters and performance in the case of glucomannan treatment in the study of Akhtar et al. (2016) [39]; general health status for all the three examined feed additives in the study of Naveed et al. (2018) [53]; immune status in the case of a *Saccharomyces cerevisiae* fermentation product containing a dose of a chlorophyll-based additive in the study of Ogunade et al. (2016) [55].

#### 3.2.5. Microbes and Enzymes

Biodegradation of aflatoxins by microorganisms and other biological organisms is an increasingly studied area as it provides an alternative for the control and elimination that is safe and has the potential not only to remove the aflatoxins, but also to extinguish its adverse health effects. Probiotic strains may also have beneficial effects on general animal health. Yeast preparations are commonly used in feed additive premixes for mycotoxin decontamination; besides, lactobacilli are well-studied for this purpose. Lactic acid bacteria (LAB) and different yeast strains are also widely used to initiate and improve silage fermentation. The decline in pH correlates with the lactic acid concentration produced by LAB, which have antimicrobial properties; besides that, yeasts may also have an adverse effect on moulds with the production of killer toxins.

In this chapter, publications found in the experimental period regarding aflatoxin-decreasing potential of microbes and enzymes are classified based on the type of organisms: yeasts, lactic acid bacteria, other microbes and enzymes of *Basidiomycota*.

The studies usually contain experiments regarding aflatoxin adsorption/binding (in vitro), antifungal activity (in vitro), aflatoxin degradation in feed or in animals (e.g., serum levels, carryover (in vivo)), animal health status (e.g., body weight gain, feed intake (in vivo)) and zootechnical parameters (e.g., dry matter, crude protein, in vitro digestibility).

In some cases, environmental effects (pH, temperature) and dose dependency (number of colony-forming units, aflatoxin concentration), etc., were also studied.

Below, a brief description of the experiments and outcomes of the studies are presented together with the most relevant data (quantified results).

Generally, all the listed publications report positive (mainly significant) results regarding aflatoxin control by microbes and/or enzymes. Besides the known and practically used microbes such as *Saccharomyces* strains, there is an abundance of promising research aiming at new candidates that are isolated from normal animal microbiota, thereby increasing the probability of colonization [56,57,58,59], while other studies focus on affordable mass production options [60]. Antimicrobial/antifungal effects of LAB were shown in the studies of Dogi et al. (2013 and 2015) [61,62], and Drobná et al. (2017) [57], while the synergistic effects of different LAB strains on aflatoxin degradation were published in the study of Zielinska and Fabiszewska (2018) [63]. Strains isolated from novel sources, such as Korean kimchi [64], Tunisian artificial butter [65,66] and feedstuff [67] are also presented. Besides novel yeast [56,68] and bacterial strains [57,58,59,60,65,67,69], the use of *Basidiomycota* extracellular enzymes has also been investigated with promising results regarding aflatoxin degradation [70,71,72]. Results regarding *Aspergillus*/aflatoxin inhibition/degradation and decreasing the adverse effects caused by aflatoxins are summarized in Table 3. Findings for other related studied topics that have been investigated in numerous publications are summarized hereunder.

##### Use of Cell-Free Supernatants

There were altogether five publications with experiments using supernatants of cultures. Three of them compared the efficiency regarding aflatoxin inhibition/degradation of supernatants versus intracellular extracts, cell pellets or viable cells [59,67,69]. In all the cases, the supernatants showed significantly better results than the others. The comparative studies were usually conducted with experimental cultures (not commonly used in practice for aflatoxin degradation at the time of publication) such as microbial consortium TMDC [69], *Bacillus shackletonii* [67] and *Escherichia coli* [59]. In the studies of Drobná et al. (2017) and Rather et al. (2014) [57,64], supernatants of *Lactobacillus* species were used, with significant results in aflatoxin reduction. This indicates that feed additives containing supernatants of bacteria may be the most effective for aflatoxin degradation.

##### Cell Viability

Yeast cell wall is often used in toxin-binding feed additive premixes; nonetheless, whole cells of microbes are used for aflatoxin-degrading experiments in many studies. Viable yeast cells showed significant results in the studies of Dogi et al. (2017) and Gonzalez Pereyra et al. (2014) [73,74]. Gonçalves et al. (2017) [76] compared different types of yeast preparations and concluded that cell wall and autolysed yeast showed high AFM1-binding capability; however, viable cells were not included in the comparison [73,74,76]. Regarding bacteria, a strong strain dependency can be seen from the results of the studies, for example, Ma et al. (2017) [78] concluded that dead *Lactobacillus plantarum* PT5B was more effective than viable cells; however, *L. plantarum* MON03 showed better results when using live cell preparations in the study of Jebali et al. (2015) [65].

##### Effect of pH and Temperature

The effect of pH on aflatoxin inhibition/degradation was studied in six publications. Strains belonging to the *Lactobacillus* genus were shown to function optimally at acidic pH (2.5–6, with the optimal value of 4) [57,61,65,78], which is favourable to tolerate gastric conditions. However, in the case of *Bacillus shackletonii* [67] and *Escherichia coli* [59], pH 8 and 8.5 proved to be effective, respectively. In these two cases, the optimal temperatures for aflatoxin-degrading enzymes proved to be effective (70 and 55 °C, respectively) than in the case of other microbes.

##### Dose Dependency

The number of initial cells reported varied in terms of the units of measurement and place of administration (depending on the experiment type), but it can be generally said that more microbes produced better results regarding aflatoxin decontamination [56,78].

For the initial aflatoxin concentration, in the case of studies examining extracellular enzymes of *Basidiomycota*, high initial aflatoxin concentrations inhibited aflatoxin degradation [71,72]. In the study of Intanoo et al. (2018) [58], inhibition at high aflatoxin concentrations was shown for bacteria; however, yeast strains functioned well at high aflatoxin concentrations as well. A positive correlation was shown for yeasts in the study of Magnoli et al. (2016) [56] as well.

## 4. Conclusions

Based on the systematic search of scientific literature, the main findings regarding intervention options for effective reduction and control of aflatoxins were identified and summarized. A detailed briefing containing data of the main results of the studies can be found in the Supplementary Materials. The identified studies in the animal feeding topic form the four main topics: low-moisture production, preservatives, acidity regulators, adsorbents and various microbiological additives.

As aflatoxins are heat-stable molecules, prevention steps are essential. Some findings indicated promising results for drying methods; however, other studies showed controversial results, meaning that the circumstances and conditions must be more precisely studied. As conventional preservatives may lead to antimicrobial resistance, naturally occurring preservatives are being more and more investigated, with positive results for essential oils and nanoparticles. Acidity regulators, as well as natural preservatives, antioxidants and flavour enhancers, were also capable of reducing aflatoxin levels in the studies found.

Using adsorbent-based feed additives is a common way of controlling aflatoxin contamination in practice. Several publications investigated the aflatoxin degradation capabilities of different clay types by themselves, in comparison with other clays, used together with other clays or other types of adsorbents or used as ingredients in feed premixes (Table 2). In conclusion, almost all the publications showed efficiency (in most cases with statistical significance) regarding aflatoxin adsorption or reduction of aflatoxin levels, transfer rate and excretion of aflatoxins. The most important concerns regarding adding adsorbents to an animal’s diet are the potential changes in the animal’s health status and zootechnical parameters as adsorbents may bind useful ingredients of the diet as well. According to the results of the studies found by systematic search, it can be concluded that no negative effects could be shown in any of the studies for any of the examined parameters. The results were mainly neutral—meaning that the feed additives did not have any adverse effects on the animals, and in some cases, on the contrary, were positive for animal health status parameters.

Using biological organisms such as microorganisms as feed additives is a well-studied area according to the results of the systematic review. Not only does it provide an option for safe prevention of aflatoxin formation and removal of aflatoxins, but it might also extinguish its adverse health effects and have beneficial effects on the general animal health. In the studies found, these were the mainly investigated areas. In general, all the listed publications reported positive (mainly significant) results regarding aflatoxin control by microbes and/or their enzymes; however, the optimal conditions of use (e.g., effect of pH and temperature, dosage) and form of usage (dead or viable cells) are strongly dependent on the type of organism. However, cell-free supernatants produced the best results in all the cases.

Ensuring product compliance is always the responsibility of the producer. Because of this and also animal welfare issues, it is of utmost importance for farmers to be able to choose the best, most suitable and fit-for-purpose animal feeding options to protect the health of the animals and thereby protect human health by placing safe milk and meat products on the market. Furthermore, in the case of aflatoxins, human health cannot be guaranteed by only maintaining the levels under the legal limits as it is a carcinogenic compound, of which any consumed amount is to be avoided. This systematic review helps feed and feed additive producers and authorities and might also help farmers or advisors of farmers, veterinarians, farmer associations to get an overview of the most suitable aflatoxin mitigation options, which will be extremely important in the near future as climate change will likely be accompanied by elevated mycotoxin levels.

## Figures and Tables

**Table 1:** Key characteristics of the interventions assessed.

Study Design	Was the Dose–Response Gradient Measured?			Sum
	No	Yes	Not Specified	
1. Experimental research	52	16		68
1.1. Randomized controlled trial	26	14		40
1.2. Challenge trial	1			1
2. Observational research	3			3
3. Narrative review	3		11	14

**Table 2:** Summary of the main findings of the studies on adsorbents and their combinations, as well as toxin-binding premixes in aflatoxin (AF) mitigation.

Adsorbents		In Vitro			In Vivo			Animal Health Status/Zootechnical Parameters				Other Experiments	Remarks
		AF Adsorption/Binding	Antifungal Activity	Ruminal Fermentation Parameters	AF Degradation/Reduction in Animals ^1^	Carryover/Transfer Rate	Reduction in AF Excretion ^2^	Blood (Plasma) Parameters ^3^	Performance, e.g., Milk Yield, Feed Intake, Milk Composition	General Health Status	Immune Status		
Alam et al. (2015) [32]	Smectite	++										Effects of glucose and ethanol on AFB1 adsorption by smectites; at least 90% of the smectites’ AFB1 adsorption capacity was preserved even with high concentrations of ethanol and glucose	
Antonelo et al. (2017) [33]	Smectite	++											Linear toxin dose effect
Maki et al. (2016a) [34]	Calcium montmorillonite				++	++		0	0				Dose-dependent reduction of the AFM1 concentration
Maki et al. (2016b) [35]	Calcium montmorillonite				++	++	++	0	0				Dose-dependent reduction of the AFM1 concentration
Pate et al. (2018) [36]	Aluminosilicate clay				++		0						
Sulzberger et al. (2017) [37]	Clay-containing vermiculite, nontronite and montmorillonite				+	+	+	+	0	0			
Soufiani et al. (2016) [38]	Activated montmorillonite clay/nonactivated montmorillonite clay/commercially available clay binder (G.Bind)				+/+/++	+/+/++							
Akhtar et al. (2016) [39]	Glucomannan/hydrated sodium calcium aluminosilicates (HSCAS)/activated charcoal							++/+/+	++/+/+				
Jiang et al. (2014) [40]	Bamboo charcoal/smectite	++/+		++/++									
Rojo et al. (2014) [41]	Aluminosilicate adsorbents/yeast cell wall glucomannan				++/+	++/+							
Kissel et al. (2012) [42]	Glucomannan and aluminosilicate blend/modified glucomannan/Alltech product (ingredients not specified in the study)/sodium bentonite				0/0/++								
Jiang et al. (2018) [43]	Bentonite clay/bentonite clay with a *Saccharomyces cerevisiae* fermentation product				+/+	+/+		+	+				
Weatherly et al. (2018) [44]	Yeast fractions and bentonite				+	0	0	+	0				A quadratic trend was observed for AFB1 presence in faeces
Ramales-Valderrama et al. (2016) [45]	*Pyracantha koidzumii* biomasses (leaves/berries/mixture of leaves and berries)	++/+/++										According to the analysis of zeta (or electrokinetic) potential, the authors concluded that the interaction type between aflatoxins and the biosorbent is primarily electrostatic. According to FTIR analysis, hydroxyl, amine, carboxyl, amide, phosphate and ketone groups are likely responsible for biosorption of AFB1 molecules	
Naseer et al. (2018) [46]	Garlic (*Allium sativum* L.)/clove (*Syzygium aromaticum*)/neem (*Azadirachta indica*)		+/+/+									According to the results of feed sample analyses (*n* = 74), in the mycotoxin-contaminated concentrate feed samples, the highest frequency of *Aspergillus* (43.3%) was observed. Out of 29 *Aspergilli*, maximum frequency (72.4%) of *A. flavus* was recorded, followed by *A. parasiticus* (13.7%), *A. fumigates* (6.8%) and *A. niger* (6.8%). Out of the total 74 concentrate feed samples collected, 67 samples had > 20 ppb of AFB1	
Fani-Makki et al. (2018) [47]	Milk thistle (*Silybum marianum*) seeds	++											The mechanism by which MT seeds decrease AFB1 is not fully understood. The presence of fibre in the seeds acting as adsorbents, silymarin, a natural polyphenolic flavonoid, and polyunsaturated fatty acids may also contribute to the beneficial characteristics regarding aflatoxin diminishing
Rychen et al. (2016) [48]	Algae interspaced bentonite	++											
Xiong et al. (2018) [49]	Solis mos (sodium montmorillonite, live yeast, yeast culture, mannan oligosaccharide and vitamin E)				++	++	++		0				
Xiong et al. (2015) [50]	Solis mos (sodium montmorillonite, live yeast, yeast culture, mannan oligosaccharide and vitamin E)			+	++	++	++			+		No effect was detected when the adsorbent was added to the diet containing a higher level of AFB1	
Jovaisiene et. al. (2016) [51]	Mycofix Plus 3.E (mineral components, biological constituent, live organism, phytogenic substances, phycophytic constituents)								0/+		+		Decrease in urea in the treatment groups, but other biochemistry data showed no change. Non-significant change in the immunity status
Aslam et al. (2016) [52]	50/50% mixture of Mycofix Secure (bentonite/dioctahedral montmorillonite) and Mycofix Plus (bentonite/dioctahedral montmorillonite, Biomin BBSH 797, Biomin MTV (*Trichosporon mycotoxinivorans* DSM 14153), phytophytic (*Ascophyllum nodosum*) and phytogenic (silymarin) substances)				+	+							
Naveed et al. (2018) [53]	Fixar Viva/Mycosorb/T5X (ingredients not specified)				++	++	++			++			
Ullah et al. (2016) [54]	Toxfin (sepiolite, bentonite and companion clays)/Elitox (enzymes, HSCAS, biopolymers, vitamin C and natural extracts)						++		+				
Ogunade et al. (2016) [55]	*Saccharomyces cerevisiae* fermentation product containing a low or high dose of a chlorophyll-based additive/or a low dose of a chlorophyll-based additive and sodium bentonite clay				0	0	0				++/+/+	When AFB1 was withdrawn from the diet, AFM1 concentrations decreased rapidly in the treatment groups, such that they fell below the FDA action level within 24 h, whereas it took 48 h in case of the control group (only the toxin)	

Legend: ++—significant; +—not significant or not indicated in the study; 0—no change; empty cell—not examined. Different results for different food additives examined in the same study are separated with “/”. ^1^ Parameters such as aflatoxin levels in blood, urine, faeces, milk. ^2^ Generally calculated from the AFM1 concentration and milk yield. ^3^ Including liver and kidney functions (if measured).

**Table 3:** Summary of the main findings of the studies on the effect of microbes and enzymes on aflatoxin (AF) mitigation.

Microbes and Enzymes		In Vitro		In Vivo					Animal Health Status/Zootechnical Parameters	Other Experiments	Remarks
		AF Adsorption/Binding	Antifungal Activity AF Inhibition	AF Degradation/Detoxification in Feed	AF Degradation/Reduction in Animals ^1^	Extinguishing AF Immunomodulation	Extinguishing AF Genotoxic Effect	Extinguishing AF Cytotoxic Effect			
Dogi et al. (2017) [73]	*Saccharomyces cerevisiae* RC016								+	AFB1 effect on *S. cerevisiae* cells—significant increase in cell diameter	
Gonzales Pereyra et al. (2014) [74]	*Saccharomyces cerevisiae* RC016						++	0	0/+		
Magnoli et al. (2016) [56]	*Clavispora lusitaniae, Pichia kudriavzevii, Cyberlindnera fabianii, Candida tropicalis*	+								AFB1 desorption study—irreversible binding was shown	All the tested strains were able to bind AFB1; however, the highest AFB1 affinity was observed for *Cl. lusitaniae* from feedstuff and the lowest value was observed for *P. kudriavzevii* from feedstuff. *Cy. fabianii* isolated from faeces and *Ca. tropicalis* isolated from the gut showed moderate affinity
Poloni et al. (2015) [75]	*Saccharomyces cerevisiae* strains RC009	0									Potentiation of a feed additive premix by different strains was investigated
Poloni et al. (2015) [75]	*Saccharomyces cerevisiae* strains RC012	++									Potentiation of a feed additive premix by different strains was investigated
Poloni et al. (2015) [75]	*Saccharomyces cerevisiae* strains RC016	++									Potentiation of a feed additive premix by different strains was investigated
Gonçalves et al. (2017) [76]	*Saccharomyces cerevisiae*				++				0		*S. cerevisiae* types studied were cell wall, dried yeast, autolyzed yeast and brewery yeast. Cell wall and autolyzed yeast showed the best results for aflatoxin reduction
Tayel et al. (2013) [68]	*Pichia anomala* ATCC 34080		+						+	Hydrolytic enzyme secretion experiment—β-1,3-glucanase and exo-chitinase activity	
Dogi et al. (2015) [62]	*Lactobacillus rhamnosus* RC007 and *Lactobacillus plantarum* RC009		++								*L. rhamnosus* RC007 was the most efficient at inhibiting the three fungal species
Zielinska and Fabiszewska (2018) [63]	*Lactobacillus buchneri* A KKP 2047 p, *Lactobacillus reuteri* M KKP 2048 p, *Lactobacillus plantarum* K KKP 593 p, *Lactobacillus plantarum* S KKP 2021 p, *Lactobacillus fermentum* N KKP 2020		++							Studies relating to the synergistic activity of bacterial strains were also conducted on a production scale. It resulted in a decrease in mould count and a decrease in AFB1 levels in silages by 74% and 75%, respectively, compared to the negative control	The bacterial strains had a synergistic effect and decreased the AFB1 levels by about 80% compared to the control silage and by about 74% compared to the silage inoculated with only one strain (*L. buchneri* A KKP 2047 p)
Ying et al. (2017) [77]	*Lactobacillus rhamnosus*			++						Fermentation characteristics, in vitro digestibility—positive effects	Reduction of aflatoxin production in silage was investigated during exposure to air
Ma et al. (2017) [78]	Ten *Lactobacillus species*	++								Viability and pH studies on binding: the greatest binding of AFB1 within a bacterium was achieved by dead *L. plantarum* and *L. buchneri* and viable *Pediococcus acidilactici* at pH 2.5. Binding efficacy generally decreased in a quadratic manner as the acidity of the culture media decreased	When applied at 10^9^ CFU/mL, all the 10 bacteria bound AFB1, but *L. plantarum* R2014 (Lp) and EQ12, *L. buchneri* R1102 (Lb) and *Pediococcus acidilactici* R2142 and EQ01 (Pa) had the greatest capacity
Drobná et al. (2017) [57]	*Lactobacillus reuteri* E and *Lactobacillus mucosae* D, *Lactobacillus murinus* C, *Lactobacillus reuteri* KO5, *Lactobacillus reuteri* KO4b, *Lactobacillus reuteri* KO4m, *Lactobacillus plantarum* KG1, *Lactobacillus plantarum* KG4	++	++							pH studies—the highest inhibition of fungal growth was observed at pH 4	The highest growth inhibition of *A. flavus* was shown by *L. mucosae* D. The best results concerning AFB1 reduction were obtained with the *L. reuteri* KO4b strain followed by *L. plantarum* KG4
Rather et al. (2014) [64]	*Lactobacillus plantarum* YML007		++	++					+		
Dogi et al. (2013) [61]	*Lactobacillus rhamnosus* RC007		++							Antibiotic resistance—no genes for resistance to the tested antibiotics	
Dogi et al. (2013) [61]	*Lactobacillus plantarum* RC009		++		-						Inhibition only at pH 4
Nasrabadi et al. (2013) [79]	*Lactobacillus casei* Shirota				+			+	++		
Jebali et al. (2015) [65]	*Lactobacillus plantarum* MON03	++				++			++		
Zhang et al. (2019) [80]	*Lactobacillus rhamnosus* GG				++			+			Single dose of AFB1 administration
Ben Salah-Abbés et. al. (2015) [66]	*Lactobacillus plantarum* MON03	++				++	++	++			Live LP showed better binding percentages than heat-killed LP
Intanoo et al. (2018) [58]	Ruminal fluid isolates—*Kluyveromyces marxianus* and *Pichia kudriavzevii* (yeast); *Enterococcus faecium, Corynebacterium phoceense* and *Corynebacterium vitaeruminis* (bacteria)			++						Preliminary assessment on biomass production—the isolates could be produced in bulk for their potential use as feed supplements for dairy cattle	The best yeast isolates were identified as *K. marxianus* and *P. kudriavzevii*. Generally, yeasts showed better detoxifying performance than bacteria in liquid media and similar but faster detoxification rates in TMR
Wang et al. (2018) [69]	Microbial consortium TMDC (*Geobacillus* (12.3%), *Tepidimicrobium* (36.65%), *Clostridium* III (21.2%), *Aeribacillus* (8.84%), *Cellulosibacter* (5.1%), *Desulfotomaculum* (6.44%) and *Tepidanaerobacter* (3.14%))			++						Simultaneous degradation of AFB1 and ZEA was studied	Cell-free supernatants, cell pellets and intracellular extracts of TMDC were studied. Supernatants of TMDC played a dominant role in mycotoxin degradation by the microbial consortium. *Geobacillus* and *Tepidimicrobium* genera played important roles in mycotoxin degradation
Wang et al. (2019) [59]	*Escherichia coli* CG1061			++						Temperature studies—the active component might be heat-resistant; pH studies—degradation rates of alkaline conditions were higher than those of acidic conditions; toxicity studies—biotransformed AFB1 was less toxic	The culture supernatant showed a significantly higher degradation rate than that of intracellular extracts
Prettl et al. (2017) [60]	*Rhodococcus pyridinivorans* K408			++						Biomass growth—changed to a stagnant state after seven days of incubation in harmony with the mycotoxin degradation rate	
Xu et al. (2017) [67]	*Bacillus shackletonii* LMG 18435			++						Enzyme characterization—thermostable enzyme named *Bacillus* aflatoxin-degrading enzyme (BADE) responsible for AFB1 degradation activity was purified and characterized	The culture supernatant of the tested isolate was more effective than viable cells and cell extracts
Scarpari et al. (2014) [70]	*Trametes versicolor* TF294, CF294		++	++						AFB1 degradation experiments with the laccase enzyme—significant decrease under in vitro and in vivo conditions (liquid culture and maize). Toxicity study of the AFB1 by-product of the laccase enzyme—no toxic effects were shown	
Das et al. (2014) [71]	*Pleurotus ostreatus* MTCC 142 and *Pleurotus ostreatus* GHBBF10			++						Effect of metal ions and surfactants on degradation—enhanced degradation was noted for *P. ostreatus* MTCC 142 in the presence of Cu^2+^ and Triton X-100 at the toxin concentration of 5 µg/mL. *P. ostreatus* GHBBF10 showed the highest degradation in the presence of Zn^2+^ and Tween 80	The highest degradation was recorded for both strains at the 0.5 µg/mL initial concentration of AFB1. With an increase in AFB1 concentration, progressive decrease in degradation was encountered
Branà et al. (2017) [72]	*Pleurotus eryngii*		++	++						Translocation of AFB1 and aflatoxicol through the thallus to the basidiocarps (fruit bodies)—neither the biomass produced on the mushroom substrate nor the mature basidiocarps contained detectable levels of AFB1 or its metabolite aflatoxicol	The addition of 5% wheat straw to the culture medium increased the tolerance of *P. eryngii* to AFB1

Legend: ++—significant; +—not significant or not indicated in the study; 0—no change; -—negative effect; empty cell—not examined. Results of the same publication are indicated with a thick frame. ^1^ Parameters such as carryover rate, aflatoxin excretion, aflatoxin levels in blood, urine, faeces, milk.

## Data Availability

No new data were created or analyzed in this study. Data sharing is not applicable to this manuscript.

## Supplementary Materials

The following supporting information can be downloaded at: https://www.mdpi.com/article/10.3390/toxins14020115/s1, Title: Detailed extracted data and information related to the intervention groups and individual interventions.

## Appendix A. Search Strategy Details: Feed and Farm

**Table A1:** Point in the food chain: storage and feed-producing facility.

Search Date	28 March 2019
Databases	EBSCOhost
Intervention phase	Storage and feed-producing facility
Search string	TI (aflatoxin OR “aflatoxin B*” OR “aflatoxin M*” OR AFM* OR AFB* OR Aspergillus) AND TI Feed AND TI (maize OR “zea mays” OR corn) AND TI (storage OR silo-bag OR rotation OR aeration OR (modif* atmosphere) OR pest control OR insect control OR rodent control OR preservation OR “aflatoxin reduc*” OR torrefaction OR irradiat* OR ammonia* OR acidificat* OR microorganism transformation OR enzymatic transformation OR (solvent extract*) OR roughage* OR forage* OR silage OR ensilage* OR silage additive* OR “by-products” OR cgf OR corn gluten feed OR ddgs OR distillers dried grain* with soluble* OR whey OR buttermilk OR permeate OR concentrate* OR biotransform* OR degrad* OR binding OR adsorbent* OR absorbent* OR clay or HSCAS OR “sodium calcium aluminosilicate” OR charcoal OR bentonite OR zeolite OR clinoptilolite OR silicate* OR chlorofillin OR “lactic acid bacteri*” OR ferment*)
Field	TI (title)
Search mode	
Filters	1 January 2013–28 March 2019
Number of records	0

**Table A2:** Point in the food chain: storage and feed-producing facility.

Search Date	28 March 2019
Databases	EBSCOhost
Intervention phase	Storage and feed-producing facility
Search string	AB (aflatoxin OR “aflatoxin B*” OR “aflatoxin M*” OR AFM* OR AFB* OR Aspergillus) AND AB Feed AND AB (maize OR “zea mays” OR corn) AND (storage OR silo-bag OR rotation OR aeration OR (modif* atmosphere) OR pest control OR insect control OR rodent control OR preservation OR “aflatoxin reduc*” OR torrefaction OR irradiat* OR ammonia* OR acidificat* OR microorganism transformation OR enzymatic transformation OR (solvent extract*) OR roughage* OR forage* OR silage OR ensilage* OR silage additive* OR “by-products” OR cgf OR corn gluten feed OR ddgs OR distillers dried grain* with soluble* OR whey OR buttermilk OR permeate OR concentrate* OR biotransform* OR degrad* OR binding OR adsorbent* OR absorbent* OR clay or HSCAS OR “sodium calcium aluminosilicate” OR charcoal OR bentonite OR zeolite OR clinoptilolite OR silicate* OR chlorofillin OR “lactic acid bacteri*” OR ferment*)
Field	AB (abstract or author-supplied abstract)
Search mode	
Filters	1 January 2013–28 March 2019
Number of records	72

**Table A3:** Point in the food chain: storage and feed-producing facility.

Search Date	28 March 2019
Databases	EBSCOhost
Intervention phase	Storage and feed-producing facility
Search string	KW (aflatoxin OR “aflatoxin B*” OR “aflatoxin M*” OR AFM* OR AFB* OR Aspergillus) AND KW Feed AND KW (maize OR “zea mays” OR corn) AND KW (storage OR silo-bag OR rotation OR aeration OR (modif* atmosphere) OR pest control OR insect control OR rodent control OR preservation OR “aflatoxin reduc*” OR torrefaction OR irradiat* OR ammonia* OR acidificat* OR microorganism transformation OR enzymatic transformation OR (solvent extract*) OR roughage* OR forage* OR silage OR ensilage* OR silage additive* OR “by-products” OR cgf OR corn gluten feed OR ddgs OR distillers dried grain* with soluble* OR whey OR buttermilk OR permeate OR concentrate* OR biotransform* OR degrad* OR binding OR adsorbent* OR absorbent* OR clay or HSCAS OR “sodium calcium aluminosilicate” OR charcoal OR bentonite OR zeolite OR clinoptilolite OR silicate* OR chlorofillin OR “lactic acid bacteri*” OR ferment*)
Field	KW (author-supplied keywords)*
Search mode	
Filters	1 January 2013–28 March 2019
Number of records	1

* Note that this search field does not exist in EBSCOhost anymore.

**Table A4:** Point in the food chain: storage and feed-producing facility.

Search Date	26 March 2019
Databases	Web of Science
Intervention phase	Storage and feed-producing facility
Search string	(((TS = (aflatoxin OR “aflatoxin B*” OR “aflatoxin M*” OR AFM* OR AFB* OR Aspergillus)) AND TS = (Feed)) AND TS = (maize OR “zea mays” OR corn)) AND TS = (storage OR silo-bag OR rotation OR aeration OR (modif* atmosphere) OR pest control OR insect control OR rodent control OR preservation OR “aflatoxin reduc*” OR torrefaction OR irradiat* OR ammonia* OR acidificat* OR microorganism transformation OR enzy-matic transformation OR (solvent extract*))
Field	TS (topic)
Filters	1 January 2013–26 March 2019
Number of records	69

**Table A5:** Point in the food chain: storage and feed-producing facility.

Search Date	26 March 2019
Databases	Web of Science
Intervention phase	Storage and feed-producing facility
Search string	(((TS = (aflatoxin OR “aflatoxin B*” OR “aflatoxin M*” OR AFM* OR AFB* OR Aspergillus)) AND TS = (Feed)) AND TS = (maize OR “zea mays” OR corn)) AND TS = (roughage* OR forage* OR silage OR ensilage* OR silage additive* OR “by-products” OR cgf OR corn gluten feed OR ddgs OR distillers dried grain* with soluble* OR whey OR buttermilk OR permeate OR concentrate* OR biotransform* OR degrad*)
Field	TS (topic)
Filters	1 January 2013–26 March 2019
Number of records	105

**Table A6:** Point in the food chain: storage and feed-producing facility.

Search Date	26 March 2019
Databases	Web of Science
Intervention phase	Storage and feed-producing facility
Search string	(((TS = (aflatoxin OR “aflatoxin B*” OR “aflatoxin M*” OR AFM* OR AFB* OR Aspergillus)) AND TS = (Feed)) AND TS = (maize OR “zea mays” OR corn)) AND TS = (binding OR adsorbent* OR absorbent* OR clay or HSCAS OR “sodium calcium aluminosilicate” OR charcoal OR bentonite OR zeolite OR clinoptilolite OR silicate* OR chlorofillin OR “lactic acid bacteri*” OR ferment*)
Field	TS (topic)
Filters	1 January 2013–26/03/2019
Number of records	127

**Table A7:** Point in the food chain: farm.

Search Date	25 March 2019
Databases	EBSCOhost
Intervention phase	Farm
Search string	AB (Aflatoxin OR Aspergillus OR AFM* OR AFB*) AND AB (milk OR cow OR cattle) AND AB (“livestock condition” OR yield* OR breed OR Holstein OR Jersey OR feeding OR feed quality OR lactation OR “carry over”)
Field	AB (abstract or author-supplied abstract)
Search mode	
Filters	1 January 2013–25 March 2019
Number of records	85

**Table A8:** Point in the food chain: farm.

Search Date	25 March 2019
Databases	EBSCOhost
Intervention phase	Farm
Search string	TI (Aflatoxin OR Aspergillus OR AFM* OR AFB*) AND TI (milk OR cow OR cattle) AND TI (“livestock condition” OR yield* OR breed OR Holstein OR Jersey OR feeding OR feed quality OR lactation OR “carry over”)
Field	TI (title)
Search mode	
Filters	1 January 2013–25 March 2019
Number of records	9

**Table A9:** Point in the food chain: farm.

Search Date	25 March 2019
Databases	EBSCOhost
Intervention phase	Farm
Search string	KW (Aflatoxin OR Aspergillus OR AFM* OR AFB*) AND KW (milk OR cow OR cattle) AND KW (“livestock condition” OR yield* OR breed OR Holstein OR Jersey OR feeding OR feed quality OR lactation OR “carry over”)
Field	KW (author-supplied keywords)*
Search mode	
Filters	1 January 2013–25 March 2019
Number of records	2

* Note that this search field does not exist in EBSCOhost anymore.

**Table A10:** Point in the food chain: farm.

Search Date	28 March 2019
Databases	Web of Science
Intervention phase	Farm
Search string	((TS = ((Aflatoxin OR Aspergillus OR AFM* OR AFB*))) AND TS = ((milk OR cow OR cattle))) AND TS = ((“livestock condition” OR yield* OR breed OR Holstein OR Jersey OR feeding OR feed quality OR lactation OR “carry over”))
Field	TS (topic)
Filters	1 January 2013–28 March 2019
Number of records	403

## Appendix B. Title and Abstract Relevance Screening Form

**Table A11:** Title and abstract relevance screening form.

Question	Options
Is the article written in English?	Yes → Proceed No → Exclude
Is the publication type other than peer-reviewed systematic review, risk assessment or primary research (e.g., editorial letter)?	• Yes → Exclude
Is contamination of non-cereal commodities discussed?	Yes → Exclude No → Proceed
Is non-feed or non-food use discussed?	• Yes → Exclude
Is the publication about aflatoxin measurement with no conclusions on the magnitude of specific intervention effects?	• Yes → Exclude
Is the publication about aflatoxin laboratory analysis?	• Yes → Exclude
Is the publication about an atomic force microscope?	• Yes → Exclude
Does the study discuss industrial utilisation (production of beneficial substances) of *Aspergillus niger*?	• Yes → Exclude

## Appendix C. Full-Text Relevance Confirmation Form

**Table A12:** Full-text relevance confirmation form.

Question	Options
Did the article investigate the effect of interventions on aflatoxins in maize or milk or milk products?	Yes → Proceed No, but the results might be extrapolated to maize or milk or milk products → Proceed No, and the results could not be extrapolated to corn or milk or milk products → Exclude
Did the article investigate the effect of interventions on aflatoxins?	Yes → Proceed No, it investigated the effect on survival/death/toxin-producing capacity of Aspergillus species, but the results might be extrapolated to levels of aflatoxins → Proceed No → Exclude
Is the text in English?	Yes → Proceed No → Exclude
Are data on the magnitude of effect of the interventions against aflatoxins available for extraction?	Yes → Proceed No → Exclude

## Appendix D. Data Extraction Form

**Table A13:** The data extraction form used during the study had the following fields.

Field	Attributes
Authors	
Title	
Published	
Point in the food chain	Values: storage and feed, farm
Intervention category	Values: 1. Feed production, 1.1. High moisture (silage/haylage/pasture), 1.2. Silage additives, 1.3. Low moisture (legume hays/fodder/straw/hulls and shells), 2. Feed additives, 2.1. Technological additives, 2.1.1. Preservatives, 2.1.2. Acidity regulators, 2.1.3. Adsorbents, 2.1.3.1. Bentonites, 2.1.3.2. Silicates, 2.1.4. Enzymes, 2.1.4.1. Extracellular enzymes of *Basidiomycota*, 2.1.5. Microbes, 2.1.5.1. Lactic acid bacteria (*Lactobacillus* and others), 2.1.5.2. Yeasts (*Saccharomyces* and others), 2.1.5.3. *Aspergillus*, 2.1.5.4. Other microbes, 2.1.6. Plant-based absorbents (biosorbents), 2.1.7. Combination of adsorbents and other technological additives, 2.2. Nutritional additives, 2.3. Combination of miscellaneous types of feed additives (e.g., toxin-binding premixes)
Intervention description	free text
Target population/sample	free text, e.g., corn, cows, etc.
Outcome measured	free text, e.g., aflatoxin M1, aflatoxin G1, Aspergillus spp., etc.
Description of the outcome	free text
Data extraction from the outcome	free text
Study design	Values: 1. Experimental research, 1.1. Randomized controlled trial, 1.2. Nonrandomized controlled trial, 1.3. Challenge trial, 1.4. Quasi-experiment, 2. Observational research, 2.1. Cohort study, 2.2. Case–control study, 2.3. Cross-sectional study, 2.4. Other, 3. Systematic review/meta-analysis, 4. Risk assessment, risk profile, cost–benefit analysis or other risk-based tool
Number (magnitude) of samples	free text
Level of data reported	Values: individual, group
Was the dose response gradient measured?	Values: yes, no, not specified
Region of the study conducted	Values: Europe, North America, South America and the Caribbean, Africa, Asia, Australia

## Appendix E

**Table A14:** PRISMA 2020 checklist.

Section and Topic	Item No.	Checklist Item	Page No. Where the Item is Reported
**TITLE**			
Title	1	Identify the report as a systematic review.	1
**ABSTRACT**			
Abstract	2	See the PRISMA 2020 for Abstracts checklist.	1
**INTRODUCTION**			
Rationale	3	Describe the rationale for the review in the context of existing knowledge.	2
Objectives	4	Provide an explicit statement of the objective(s) or question(s) the review addresses.	2–3
**METHODS**			
Eligibility criteria	5	Specify the inclusion and exclusion criteria for the review and how studies were grouped for the syntheses.	3–5, 27 (Appendix B and Appendix C)
Information sources	6	Specify all databases, registers, websites, organisations, reference lists and other sources searched or consulted to identify studies. Specify the date when each source was last searched or consulted.	3, 23–26 (Appendix A)
Search strategy	7	Present the full search strategies for all databases, registers and websites, including any filters and limits used.	3, 23–26 (Appendix A)
Selection process	8	Specify the methods used to decide whether a study met the inclusion criteria of the review, including how many reviewers screened each record and each report retrieved, whether they worked independently, and if applicable, details of automation tools used in the process.	2–3
Data collection process	9	Specify the methods used to collect data from reports, including how many reviewers collected data from each report, whether they worked independently, any processes for obtaining or confirming data from study investigators, and if applicable, details of automation tools used in the process.	4, 28 (Appendix D)
Data items	10a	List and define all outcomes for which data were sought. Specify whether all results that were compatible with each outcome domain in each study were sought (e.g., for all measures, time points, analyses), and if not, the methods used to decide which results to collect.	3, Table 2 and Table 3
	10b	List and define all other variables for which data were sought (e.g., participant and intervention characteristics, funding sources). Describe any assumptions made about any missing or unclear information.	4, 28 (Appendix D)
Study risk of bias assessment	11	Specify the methods used to assess risk of bias in the included studies, including details of the tool(s) used, how many reviewers assessed each study and whether they worked independently, and if applicable, details of automation tools used in the process.	4,
Effect measures	12	Specify for each outcome the effect measure(s) (e.g., risk ratio, mean difference) used in the synthesis or presentation of results.	2, not applicable
Synthesis methods	13a	Describe the processes used to decide which studies were eligible for each synthesis (e.g., tabulating the study intervention characteristics and comparing against the planned groups for each synthesis (item #5)).	not applicable
	13b	Describe any methods required to prepare the data for presentation or synthesis, such as handling of missing summary statistics, or data conversions.	not applicable
	13c	Describe any methods used to tabulate or visually display results of individual studies and syntheses.	4–5, Table 2 and Table 3, Supplementary Materials
	13d	Describe any methods used to synthesize results and provide a rationale for the choice(s). If meta-analysis was performed, describe the model(s), method(s) to identify the presence and extent of statistical heterogeneity, and software package(s) used.	not applicable
	13e	Describe any methods used to explore possible causes of heterogeneity among study results (e.g., subgroup analysis, meta-regression).	not applicable
	13f	Describe any sensitivity analyses conducted to assess robustness of the synthesized results.	not applicable
Reporting bias assessment	14	Describe any methods used to assess risk of bias due to missing results in a synthesis (arising from reporting biases).	not applicable
Certainty assessment	15	Describe any methods used to assess certainty (or confidence) in the body of evidence for an outcome.	not applicable
**RESULTS**			
Study selection	16a	Describe the results of the search and selection process, from the number of records identified in the search to the number of studies included in the review, ideally using a flow diagram.	4
	16b	Cite studies that might appear to meet the inclusion criteria, but which were excluded, and explain why they were excluded.	3–4
Study characteristics	17	Cite each included study and present its characteristics.	Table 2 and Table 3, Supplementary Materials
Risk of bias in studies	18	Present assessments of risk of bias for each included study.	not applicable
Results of individual studies	19	For all outcomes, present, for each study: (a) summary statistics for each group (where appropriate) and (b) an effect estimate and its precision (e.g., confidence/credible interval), ideally using structured tables or plots.	not applicable
Results of syntheses	20a	For each synthesis, briefly summarise the characteristics and risk of bias among contributing studies.	not applicable
	20b	Present results of all statistical syntheses conducted. If meta-analysis was done, present for each the summary estimate and its precision (e.g., confidence/credible interval) and measures of statistical heterogeneity. If comparing groups, describe the direction of the effect.	not applicable
	20c	Present results of all investigations of possible causes of heterogeneity among study results.	not applicable
	20d	Present results of all sensitivity analyses conducted to assess the robustness of the synthesized results.	not applicable
Reporting biases	21	Present assessments of risk of bias due to missing results (arising from reporting biases) for each synthesis assessed.	not applicable
Certainty of evidence	22	Present assessments of certainty (or confidence) in the body of evidence for each outcome assessed.	not applicable
**DISCUSSION**			
Discussion	23a	Provide a general interpretation of the results in the context of other evidence.	22
	23b	Discuss any limitations of the evidence included in the review.	13
	23c	Discuss any limitations of the review processes used.	3
	23d	Discuss implications of the results for practice, policy, and future research.	22
**OTHER INFORMATION**			
Registration and protocol	24a	Provide registration information for the review, including register name and registration number, or state that the review was not registered.	not registered
	24b	Indicate where the review protocol can be accessed, or state that a protocol was not prepared.	27–28 (Appendix B, Appendix C and Appendix D)
	24c	Describe and explain any amendments to information provided at registration or in the protocol.	not applicable
Support	25	Describe sources of financial or non-financial support for the review, and the role of the funders or sponsors in the review.	22
Competing interests	26	Declare any competing interests of review authors.	22
Availability of data, code and other materials	27	Report which of the following are publicly available and where they can be found: template data collection forms; data extracted from included studies; data used for all analyses; analytic code; any other materials used in the review.	28 (Appendix D), Supplementary Materials
