# Supplementary material for: A Systematic Review of the Efficacy of Interventions to Control Aflatoxins in the Dairy Production Chain—Feed Production and Animal Feeding Interventions
Source: Toxins (Basel). 2022 Feb 3;14(2):115. doi: 10.3390/toxins14020115 (PMC8878089; doi:10.3390/toxins14020115)
Supplement: Supplementary file 1 [file toxins-14-00115-s001.zip › toxins-1565954-supplementary.pdf]

toxins MDPI

Supplementary Materials:

A Systematic Review of the Efficacy of Interventions to Control Aflatoxins in Dairy Production Chain – Feeding and Farm Interventions

Zsuzsa Farkas, Erika Országh, Tekla Engelhardt, Szilveszter Csorba, Kata Kerekes, Andrea Zentai, Miklós Süth, Attila Nagy, Gabriella Miklós, Krisztina Molnár, Csaba Rácz, Tamás Dövényi-Nagy, Árpád Ambrus, Zoltán Győri, Attila Csaba Dobos, Tünde Pusztahelyi, István Pócsi and Ákos Jóźwiak

Detailed extracted data and information related to the intervention groups and individual interventions

Feed production and animal feeding interventions

The risk reduction interventions of aflatoxins during feed production and animal feeding encompass a multitude of various methods. The most important identified groups of interventions, based on their mode of action and place in the technological process are: low moisture production, using preservatives, acidity regulators, adsorbents and various microbiological additives.

1. Low moisture production

The rapid drying of agricultural products to reduce their moisture content is an important method, which prevents the formation of favorable conditions for the growth of fungi.

Chiewchan et al. (2015) [1] reviewed the possibilities of the application of different drying methods to control mould growth and aflatoxin production. The first method was drying sheep feed composed of crushed maize, wheat bran and peanut meal as a thin layer using hot air oven at 80°C for 6 h. This technology resulted in a 57.6% reduction of aflatoxins. The second method, drying under sunlight at ambient temperature for 2 days, resulted in an 83.7% reduction of aflatoxins. However, in other studies, drying whole maize kernels at 40–70°C, presented no eradication efficacy on aflatoxins.

2. Preservatives

Feed additives are chemical compounds e.g., different acids, bases, aldehydes, ozone, hydrogen peroxide or various types of gases which are able to degrade mycotoxins. It has been proven that aqueous extracts also have chemically active properties against them. The inactivation of aflatoxins may be a consequence of hydrolysis of the aflatoxins lactone ring, resulting in the conversion of the parent compound into several less toxic products [2].

Testing of naturally occurring antimicrobials for the preservation of food and feed products receives increasing attention due to the growing concern of microbial resistance towards conventional preservatives. The safety of chemical preservatives generated a strong debate since they are considered responsible for many carcinogenic and teratogenic attributes as well as residual toxicity.

Koc and Kara (2014) [3] investigated the antifungal potential of thyme, laurel, and rosemary essential oils against *Aspergillus flavus* and *Aspergillus parasiticus* in order to use them as an alternative to preservatives such as potassium sorbate.

Each essential oil – especially in higher concentrations – showed antifungal activities against *A. parasiticus* and *A. flavus*. The storage time had no significant effect on the antifungal activity. The most promising essential oil was Thyme, which had the highest

inhibition of mould growth at all concentrations, followed by the preservative, potassium sorbate, then Rosemary and Laurel oil. At the highest concentration (120 mg/mL), the inhibition zones against *Aspergillus flavus* were 40 mm, 25 mm, 15 mm and 10 mm, respectively. Thyme essential oil have notable antimicrobial and antioxidant properties that may be utilized in the food industry.

In another study, conducted by Garcia et al. (2012) [4] the antifungal capability of Equisetum arvense extract was tested against A. flavus. The inhibitory effect of the extract was only observed in the inoculated treatments, regardless of the incubation time, with a 45% population reduction. Therefore, the E. arvense extract has proven to be effective in stored maize with high mould levels and accordingly, it could be an alternative to synthetic fungicides. Although, the effect of the E. arvense extract on mycotoxin production in maize could not be confirmed as no aflatoxins were detected in any of the treatments or in the controls.

The effectiveness of four food additives were tested by Shi et al. (2017) [5] in distillers wet grains (DWG) and condensed distillers solubles (CDS), namely sodium bisulfite, sodium hypochlorite, citric acid, and ammonium persulfate. According to the results, sodium bisulfite was not highly efficient in degrading aflatoxin neither in DWG (24% reduction) nor in CDS (35%). Among the four food additives tested, sodium hypochlorite was the most effective (42% reduction in DWG and 56% in CDS), but it bleached the substrate and left an off-odor, therefore, the authors concluded, sodium hypochlorite is not suitable for aflatoxin degradation in food and feed products. Citric acid and ammonium persulfate reduced aflatoxin levels by 31 to 51% and the effect of the citric acid could be further enhanced by increasing the addition level and prolonging the heating time. Reduction levels of 65 and 80% in DWG and CDS, respectively, were obtained by the addition of 2.5% (by weight) citric acid and increased heating at
90°C for 1 h. Therefore, citric acid appeared to be the most promising additive for degrading aflatoxin in distillers grain.

Ashgar et al. (2018) [6] investigated the antifungal activity and reduction of aflatoxin production of iron (Fe), copper (Cu) and silver (Ag) nanoparticles (NPs) extracted from green tea and black tea leaves.
Ag-NPs showed the highest antifungal activities and aflatoxin reduction in comparison to Fe-NPs and Cu-NPs. The latter was directly proportional to the amount of NPs. The synthesized metal nanoparticles found to be effective in controlling phytopathogenic fungi in plants and AFs contamination.

The study also examined the effect of NPs on aflatoxin B1 adsorption activity in different conditions. The adsorption activities of the metal NPs followed the order of Fe-NPs > Cu-NPs > Ag-NPs, but they were not significantly different from each other at $p \leq 0.05$.

Higher adsorption of AFB1 was achieved with an increase in the solution pH, incubation time and temperature. The adsorption of AFB1 decreased with higher initial AFB1 concentration. The study concluded that metal NPs may be utilized as a possible aflatoxins adsorbent in human food and animal feed such as rice, wheat, maize, red chillies and poultry feed.

3. Acidity regulators

Organic acids are natural preservatives and antioxidants and are present in food as common constituents or could be added artificially to enhance their flavour. They are also suitable in aiding the degradation of AFB1 in food.

Aiko et al. (2016) [7] investigated the effect of three different organic acids on the degradation of AFB1, at high temperature (heating at 80°C for 10, 30 and 60 min). The results showed that among acetic, citric and lactic acid, the latter was the most efficient in degrading AFB1, and the efficacy increased with concentration, heating temperature and time period. The most effective degradation of AFB1 was observed at 1 mol L^{-1} concentration of lactic acid, when heated for 60 min to 80°C.

A cytotoxicity assay was also carried out to confirm if degradation of AFB1 by lactic acid resulted in detoxification. The 10 min lactic acid treatment degraded AFB1 by 10%, which resulted in 92% cytotoxicity, a 90 min treatment resulted 77% degradation with 23% cytotoxicity.

Singh and Mandal (2014) [8] studied the efficacy of fumaric and citric acids in preventing biosynthesis of aflatoxins in poultry feed. The results showed that at 11% moisture level, none of the studied aflatoxins were recorded in any of the treatments, but with the increase in moisture in feed, the production of aflatoxins also increased. The biosynthesis of all the aflatoxins was inhibited with 0.20% fumaric acid or 0.45% citric acid in feed at 13% moisture level. However, both fumaric and citric acid could only decrease, but not inhibit the synthesis of aflatoxins in 0.50% concentration at 15 and 17% moisture level. It has been concluded that storage of feed for 1 month with 13% moisture content is only safe if the production of AF is inhibited by adding fumaric acid at 0.20% or citric acid at 0.45% concentration. At 15 and 17% moisture level, more than 0.50% of fumaric acid or citric acid is required for complete inhibition of biosynthesis of aflatoxins.

Propionic acid is a very effective and cheap mould inhibitor, however, it has an unpleasant odour and corrosive nature, which hinders its use in food and feed products. This problem can be solved by the use of its salts, sodium and calcium propionates, which have no offensive odour and are not corrosive but have fungistatic effect.

Alam et al. (2014) [9] studied the effects of calcium propionate, water activity (a_w) and incubation time on the total fungal count and aflatoxins B1 (AFB1), B2 (AFB2), G1 (AFG1) and G2 (AFG2) production in broiler feed. They investigated the application of two levels (0 and 5 g/kg of feed) of calcium propionate on contaminated feed at three different a_w levels (0.85, 0.90 and 0.95 a_w) for 28 days at 25°C. All the factors (preservative, aw and storage time) alone and in combination significantly reduced the total fungal count and aflatoxins production in the feed. The total fungal counts on day 1 were 2.29×10^2, 2.57×10^2 and 2.39×10^2 CFU/g, at 0.85, 0.90 and 0.95 a_w and increased to 31.62×10^7, 263.03×10^7 and 436.51×10^7 CFU/g feed by Day 28 in the control samples. But in preserved feed, the total fungal count reached only 22.90×10^4, 45.71×10^4 and 89.12×10^4 CFU/g at 0.85, 0.90 and 0.95 a_w. The AFB1 content increased from 11.35 to 73.44, from 11.58 to 81.81 and from 11.54 to 102.68 ng/g in the control samples, whereas in preserved feed the content of B1 increased from 11.47 to 37.83, from 11.54 to 49.07 and 11.20 to 53.14 ng/g at 0.85, 0.90 and 0.95 a_w, respectively. Similar patterns were observed for AFB2, AFG1 and AFG2. All the aflatoxins increased over storage time; however, the increase was moderate in preserved feed that contained a lower amount of available water. This study has proven that calcium propionate along with decreased water activity can serve as an effective tool for controlling mould incidence and aflatoxin production.

Lee, Her, and Lee (2015) [10] observed high reduction (93–95%) of aflatoxin levels after treating soybean with 1.0 N citric, lactic and tartaric acid for 18 h.

4. Adsorbents and adsorption-based feed additive combinations

4.1. Clay-based adsorbents

4.1.1. Studies evaluating a single clay-based adsorbent

Alam et al. (2015) [11] assessed the AFB1 adsorption capacity of smectite active mineral of bentonite clay and the inhibition effect of ethanol and glucose on the AFB1 adsorption, as these two compounds might compete for clay adsorption sites during fermentation processes in biofuel production. As the aflatoxins are concentrated in the co-product distiller's grain, which is often used as feed, detoxification is crucial. Aflatoxin adsorption capacities of four smectites from Mississippi (Bentonite 2MS, 3MS, and 4MS - branded as NovaSil™ provided by Office of the Texas State Chemist). Bentonite MBBO1 mined in Alabama and supplied by the American Colloid Company. Smectites were evaluated in water and in 10% ethanol and 20% glucose solutions. According to the results, maximum adsorption capacity of 0.59 mol/kg (18% by weight) was found for one of the Mississippi

bentonites 3MS in water. The adsorption capacity varied from 0.43–0.59 mol/kg among the four smectites, therefore all of them are considered to be good adsorbents (>0.3 mol/kg) Regarding the effects of glucose and ethanol on AFB1 adsorption by smectites, at least 90% of the smectite's AFB1 adsorption capacity was preserved even with such high concentrations of ethanol and glucose. The study also shows that aflatoxins did enter the interlayer spaces of the smectites despite the presence of high concentrations of ethanol and glucose. They also evaluated the effect of glucose and ethanol on the AFB1-smectite bonding by Fourier-transform infrared spectroscopy (FTIR) spectroscopy and found that the smectites were selective for aflatoxins in the presence of ethanol and glucose, however, the AFB1 band positions were influenced by the types of exchange cations and humidity.

In the study of Antonelo et al. (2017) [12], toxin binding capacity of smectite clay was investigated in vitro. Aflatoxin B1 was intubated with 150 mg clay in 10 mL ruminal fluid obtained from ruminally cannulated cows. This ratio represents the concentration ~1% clay inclusion treatment. Three concentrations (52, 520, and 5,200 μg/mL) of AFB1 were tested. The percentage of adsorbed toxins was calculated according to Joannis-Cassan et al. (2011) [13] using the following equation:

$$\text{percent adsorption} = (C_{ads}/C_0) \times 100 = [(C_0 - C_{eq})/C_0] \times 100, \quad \text{equ. 1} \tag{1}$$

Copyright: © 2022 by the authors. Licensee MDPI, Basel, Switzerland. This article is an open access article distributed under the terms and conditions of the Creative Commons Attribution (CC BY) license (https://creativecommons.org/licenses/by/4.0/).

in which C_{ads} is the concentration (μg/mL) of adsorbed toxins, C0 is the concentration (μg/mL) of toxins in the supernatant of the blank control (with no clay), and C_{eq} is the toxin concentration (μg/mL) in the supernatant of the sample corrected for the basal toxin concentration of the ruminal fluid. According to the results, clay bound 100.1 and 98.8% of aflatoxin when dosed at 52 or 520 μg/mL but only 35.5% when dosed at 5,200 μg/mL (linear toxin dose effect, $p < 0.01$). The authors concluded that smectite reaches its adsorption maximum at concentrations higher than would occur under practical conditions and what is considered toxic (300 ng/mL of AFB1, Fraser, 1991 [14]), therefore it is capable of preventing toxicosis from ruminal adsorption of aflatoxins.

Maki et al. (2016b) [15] studied the effects of calcium montmorillonite clay (NovaSil Plus, NSP, BASF Chemical Company (Ludwigshafen, Germany)) on the reduction of AFM1 in milk of dairy cows. Each experimental period included a treatment period of 7 days, followed by a 5-day depletion period. 15 animals were divided into 5 treatment groups: control (no aflatoxin or NSP), aflatoxin control (121 ppb), NSP control (12.1 g/kg), high dose NSP (aflatoxin + 12.1 g/kg NSP) and low dose NSP (aflatoxin + 6.0 g/kg NSP). The results showed that both NSP treatments reduced AFM1 concentrations in milk significantly ($p < 0.0001$), in a dose-dependent manner, by 55% and 68% in cases of low-dose NSP and high-dose NSP, respectively. Transfer rate from feed to milk was reduced ($p < 0.0001$) from 1.13% in the aflatoxin diet, to 0.35 and 0.52% with the inclusion of NSP at 12.1 and 6.0 g/kg of dry matter intake (DMI). Concentrations of milk composition, vitamin A, and riboflavin; and dry matter intake and milk yield were unaffected by any of the treatments.

In, Maki et al. (2016a) [16] the authors published an experiment investigating the effects of calcium montmorillonite clay (NovaSil Plus, NSP) aflatoxin concentrations, dry matter intake, milk production and milk composition on fifteen primiparous crossbred dairy cows. There were five treatment groups; 1. control (CON, receiving only TMR); 2. high-dose NSP diet (NSP-1%), consisting of total mix ration (TMR) plus 230 g of NSP; 3. aflatoxin diet (AFD), consisting of the TMR plus aflatoxin challenge; 4. low-dose NSP with aflatoxin (NSP-0.5% + AFD) composed of TMR plus 115 g of NSP and AF challenge and 5. high-dose NSP with AF (NSP-1% + AFD), consisting of TMR plus 230 g of NSP and

aflatoxin challenge. The AF challenge consisted of top dressing a daily dose of 100 μg/kg estimated dry matter intake (DMI); similarly, NSP was fed at 1.0 or 0.5% of estimated DMI. The authors reported a dose-dependent reduction ($p < 0.01$) in AFM1 concentrations with NSP inclusion, the decrease was 47.3% and 70.9% when NSP was fed at 0.58% and 1.17% of DMI, respectively. The transfer rate was reduced from 1.07% (AFD treatment) to 0.52% and 0.32% (low-dose and high-dose NSP treatments, respectively). Because of the reduced transfer rate, total AFM1 excretion (calculated with equ. 1) was also reduced ($p < 0.01$) in a dose-dependent manner, by 51.3% and 69.7% in low- and high-dose NSP treatments, respectively. The authors concluded that using NSP feed additives in case of lactating cows is effective in the reduction of transfer and excretion of AFM1 in milk with no deleterious effects on milk production and composition including vitamin A, riboflavin, and mineral content.

In the study of Pate et al. (2018) [17], the effects of a commercially available aluminosilicate clay were investigated in terms of production responses, blood chemistry, and liver inflammatory markers. AFM1 levels in milk, urine, feces and aflatoxin transfer were also investigated. 16 lactating Holstein cows were divided into four groups: no adsorbent and no AF challenge (CON), no adsorbent and an AF challenge (POS), 113 g of aluminosilicate clay (adsorbent) top-dressed on the total mixed ration with an AF challenge (F4), or 227 g of adsorbent with an AF challenge (F8). The challenge consisted of 100 μg of AFB1/kg of dietary dry matter intake administered orally. For each 21-day treatment period, milk was sampled 3 times daily from day 14 to 21; blood, feces, and urine were sampled on day 14, 18, and 21. The results showed that cows receiving aflatoxin challenge and adsorbent had lower AFM1 concentrations on day 18 in milk (POS = 0.33, F4 = 0.32, and F8 = 0.27 μg/kg) and urine (POS = 2.10, F4 = 1.89, and F8 = 1.78 μg/kg) and lower AFB1 concentrations in feces (POS = 4.68, F4 = 3.44, and F8 = 3.17 μg/kg). Significant decrease was observed in milk AFM1 proportion of 3% (F4), 19% (F8) at peak exposure (day 18). The excretion of AFM1 was calculated in comparison with Maki et al. (2016b) [15] when adsorbent was added to the diet. The authors concluded no difference in AFM1 excretion caused by adding adsorbent to the diet.

Sulzberger et al. (2017) [18] examined the effects of dietary clay (containing vermiculite, nontronite, and montmorillonite) in three different concentrations on aflatoxin excretion, blood chemistry, immune response, milk composition, and health of mid-lactation Holstein cows after an aflatoxin challenge. The challenge consisted of 100 μg of aflatoxin B1 (AFB1)/kg of dietary dry matter intake for 3 days. The material was fitted into 10-mL gelatin capsules and administered into the rumen through a rumen-cannula. There were five treatments; no clay plus an AF challenge (POS); 3 different concentrations of clay (0.5, 1, or 2% of dietary DMI) plus an AF challenge; and a control consisting of no clay and no AF challenge (C). Two contrasts, CONT1 (POS vs. C) and CONT2 (POS vs. the average of 0.5, 1, and 2% clay), were compared in the statistical analysis. Cows supplemented with clay had lower aflatoxin excretion in milk as aflatoxin M1 (AFM1; 0.5% = 20.83 μg/d, 1% = 22.82 μg/d, and 2% = 16.51 μg/d) and aflatoxin transfer from rumen fluid to milk (AFM1; 0.5% = 1.01%, 1% = 0.98%, and 2% = 0.74%) compared with cows in POS group (AFM1 = 27.81 μg/d and aflatoxin transfer = 1.37%, CONT2). Similarly, concentrations of AFM1 in milk (0.5% = 0.35 μg/kg, 1% = 0.30 μg/kg, 2% = 0.25 μg/kg), AFB1 in feces (0.5% = 1.79 μg/g, 1% = 1.52 μg/kg, 2% = 1.48 μg/kg), and AFB1 in rumen fluid (0.5% = 0.05 μg/kg, 1% = 0.02 μg/kg, 2% = 0.02 μg/kg) were reduced in cows fed clay compared with POS group (0.43 μg/kg, 2.78 μg/kg, and 0.10 μg/kg, respectively, CONT2). The authors concluded that the clay product did not improve the lactation and the health status of the cow when being challenged with aflatoxin. Aflatoxin concentrations in rumen, milk and feces decreased, however, there was no reduction in adsorption of analysed vitamins. An improved liver function was shown in cows in clay-treated groups.

4.1.2. Studies comparing different clay-based adsorbents

Soufiani et al. (2016) [19] investigated the effects of activated clay in comparison to both non-activated and commercially available clay binders on AFB1 adsorption and transport rate from feed to milk in Holstein dairy cows. The experiment was conducted on 12 animals for two weeks after a week of adaptation period. The toxin has been added to the diets via a source of cottonseed meal, which had been naturally contaminated with AFB1. The three binders, 1) unprocessed montmorillonite (Mt) (F), 2) imported commercially available clay (M), and 3) local processed Mt (G.Bind™) were added to the diets according to recommendations of producing companies. According to the results, all binders were able to decrease AFM1, but significant decrease was only shown in case of G.Bind™ ($p < 0.05$). G.Bind™ lowered the transfer rate of aflatoxin B1 from 1.17% at the beginning of the experiment to 0.43% and 0.39% after the first and second weeks, respectively. The decrease in transfer rates were only significant ($p < 0.05$) in case of this binder. In conclusion, processing of bentonites (basic processing in present study) can considerably help in aflatoxin adsorption from feed and also in decreasing aflatoxin transfer to milk.

4.1.3. Comparison of different adsorbent types (clay-based, glucomannan, clay + glucomannan, charcoal)

Akhtar et al. (2016) [20] assessed the physiological responses of Nili-ravi buffaloes against AFB1 for three adsorbents, glucomannan, hydrated sodium calcium aluminosilicates (HSCAS, smectite) and activated charcoal. They investigated serum levels and complete blood count on five different groups. The control group contained no AFB1 and no adsorbent. The remaining four contained 500 µg/kg AFB1 per animal per day. There was a positive control group with no adsorbent, in the remaining three, adsorbents were added at an inclusion level of 0.2% of feed dry matter per animal per day. The results show that the average daily feed intake of AFB1-treated buffaloes significantly declined ($p < 0.05$) compared with the control. Serum levels of total bilirubin, alkaline phosphatase (ALP), alanine aminotransferase (ALT), aspartate aminotransferase (AST) and creatinine were significantly increased ($p < 0.05$) in response to AFB1, but the change in blood urea nitrogen (BUN) levels was nonsignificant ($p > 0.05$). In haematology, the total erythrocyte count (TEC), total leukocyte count (TLC), haemoglobin concentration (HGB), haematocrit levels (HCT), mean corpuscular haemoglobin (MCH) and mean corpuscular haemoglobin concentration (MCHC) decreased significantly ($p < 0.05$) in the AFB1 group on days 14 and 21 of the experiment. From among the three toxin adsorbents, 0.2% glucomannan significantly ($p < 0.05$) improved the feed intake levels of buffaloes and also significantly ($p < 0.05$) minimized the AFB1-induced adverse effects on haematology and serum biochemistry, but was unable to totally ameliorate its effect, while HSCAS was less effective, and activated charcoal, the least.

In the study of Jiang et al. (2014) [21], bamboo charcoal was compared to a smectite clay product regarding AFB1 adsorption capacity and the ability to reduce the adverse effects of AFB1 on rumen fermentation. From the two in vitro experiments, the first was an equilibrium adsorption test, where the adsorption capacity and proportion adsorbed (0.381 µg/mg and 0.955, respectively) for bamboo charcoal were found to be greater than for smectite clay (0.372 µg/mg and 0.931, respectively). In the second experiment, the effects of in vitro ruminal fermentation of hay-rich feed incubated with 1.0 µg/mL AFB1 for 0, 0.1, 1 and 10 g/L doses of bamboo charcoal and smectite clay were measured. According to the results, both binders significantly increased AFB1 loss at dosages ≥ 1.0 g/L. Average AFB1 loss ($p < 0.0001$) was greater for smectite clay (0.904) than for bamboo charcoal (0.881). Both materials similarly increased in vitro dry matter loss. Asymptotic gas volume and volatile fatty acid production were greater for bamboo charcoal than for smectite clay. Overall, the authors concluded that bamboo charcoal can be as effective as smectite in removing the adverse effects of AFB1 on rumen degradability and fermentation under the occurrence of microbial aflatoxin degradation.

Rojo et al. (2014) [22] investigated the carry-over rate of aflatoxin B1 to aflatoxin M1 in two comparative in vivo experiments conducted on Holstein cows in order to compare

the artificial aflatoxin B1 exposure methods. The adsorption effects of two commercial aluminosilicate adsorbents (0.2% dry matter intake) and one commercial yeast cell wall glucomannan (0.075% dry matter intake) was also investigated. In both experiments, one positive control group and the three treatment groups (according to the type of binders) received 40 μg/kg AFB1 daily for 5 days after 6 days of depletion period. According to the results of the study, the carryover rates for the control groups were 3.35 and 1.8 % in the first and second experiments, respectively. The authors concluded that the exposure method of the second experiment was in line with previous studies mainly conducted with naturally contaminated feed. The main differences were that in the second experiment, AFB1 cultured rice powder was mixed into TMR, on the contrary, in the first experiment, AFB1 cultured rice was only mixed with grain concentrate and fed separately. The higher observed levels of AFM1 in milk in the first experiment may be caused by a fast passage of AFB1 through the gastrointestinal tract, and enhanced AFB1 oxidation process.

According to the results of the experiments, that can be seen in Table S1, unlike yeast cell wall glucomannan, both aluminosilicate adsorbents were found to significantly reduce the carry-over of AFM1 to raw milk ($p < 0.05$).

Table S1. Results of the experiment of Rojo et al. (2014) [22] 'Carryover of AFB1 to AFM1 in milk and percent of reduction of AFM1 in cow's milk (%)'.

	**Carry-Over**		**Reduction of AFM1 Milk**	
Treatments	Exp. No. 1	Exp. No. 2	Exp. No. 1	Exp. No. 2
T1 = AFB1	3.4	1.8	-	-
T2 = AFB1 + AA-1	2.7	1.2	19.3	33.2
T3 = AFB1 + AA-2	2.8	1.2	17.6	36.8
T3 = AFB1 + Glucomannan derived from yeast cell walls	2.9	1.6	11.2	11.8

Exp = Experiment; AA = Aluminosilicate adsorbent

In the publication of Kissel et al. (2012) [23], three experiments on the effects of different feed additives on AFM1 concentration were demonstrated. All experiment lasted for two weeks, when animals received aflatoxin contaminated TMR, after a depletion period. 12 and 2–5 Holstein cows formed a treatment group in experiments 1–2 and 3, respectively. In the experiments, treatment groups were control (no feed additive), 100g/cow daily experimental Lallemand product (a blend of yeast cell wall extract – glucomannan and aluminosilicate), 10 g/cow daily of MTB-100-2004 (Alltech, Inc., a modified glucomannan product (also known as Integral or Mycosorb in some countries), 10 g/cow daily of MTB-100-2006, (Alltech, Inc., MTB-100-2004 and MTB-100-2006 were commercially available formulations during the years 2004 and 2006), 10 g/cow daily of experimental Alltech product (Alltech, Inc., ingredients not specified in the study), and 227 g/cow daily of Astra-Ben 20 (AB-20; Prince Agri Products, Inc., sodium bentonite). Corn naturally contaminated with aflatoxin was used as AFB1 contamination source added to TMR. Animals received feed additive only on the second week and AFM1 concentrations were measured 2 times on the first week and the second week as well. According to the authors' conclusions, only sodium bentonite (AB-20) fed at 227 g/cow daily reduced AFM1 concentrations significantly (by 60.4%) when cows were fed diets containing 86.0 ppb of AFB1. Other four feed additives used in this study did not affect AFM1 concentrations.

In the experiment of Jiang et al. (2018) [24], the effects of bentonite clay supplementation with or without a *Saccharomyces cerevisiae* fermentation product on the performance and health of dairy cows challenged with aflatoxin B1 (AFB1) were examined. Twenty-four lactating Holstein cows (64 ± 11 day in milk) were involved in the study. There were four 33-day periods (1–25 days adaptation period, 26–30 days AFB1 dosing, 31–33 days withdrawal) and 5-day washout periods in between. Treatments were: 1. control (no additives), 2. toxin (T; 1,725 μg of AFB1/head per day), 3. T + clay (CL; Astra-Ben-20, Prince

Agri Products Inc., Quincy, IL, 200 g/head per day; top-dressed), and 4. CL+*Saccharomyces cerevisiae* fermentation product, SCFP (SCFP is a proprietary product containing 19 g of NutriTek and 16 g of MetaShield (Diamond V, Cedar Rapids, IA) (CL+SCFP; 35 g/head per day; top-dressed). Milk samples were collected twice daily from d 21 to 33, and plasma was sampled on d 25 and 30. Aflatoxin transfer to milk was higher in T than in CL or CL+SCFP (1.65 vs. 1.01 and 0.94%, respectively). Milk AFM1 concentrations were reduced in CL and CL+SCFP treatments compared with T (0.45 and 0.40 vs. 0.75 μg/kg, respectively). The authors stated that in their experiment, dietary addition of AFB1 increased milk AFM1 concentration beyond the FDA action level (0.5 μg/kg). CL alone or combined with SCFP were able to reduce AFM1 concentration such that mean concentrations were below the action level. Milk yield tended to be greater for cows fed with CL+SCFP than those in treatment T. The concentration of plasma glutamic oxaloacetic transaminase was reduced when CL was fed compared with T.

The effects of two adsorbents, containing yeast fractions and bentonite on health and performance of lactating Holstein cows were investigated during an aflatoxin challenge in the study of Weatherly et al. (2018) [25]. 76 animals were separated into 5 treatment groups: 1. no adsorbent or aflatoxin: control (CON); 2. no AD plus AF challenge: positive control (POS); 3. 30 g per cow per day of adsorbent 1 (AD1, proprietary composition of yeast cell wall and bentonite clay) and 4. AF challenge (P30); 60 g per cow per day of AD1 and AF challenge (P60) and 5. 60 g per cow per day of adsorbent 2 (prototype adsorbent, AD2 and AF challenge (PROT). The experiment lasted for 28 days. Measurements were made from day 22 to day 28. Aflatoxin challenge meant 100 μg of AFB1/kg of diet dry matter administered orally from day 22 to day 24. Aflatoxin excretion and transfer were calculated according to the equation described by Maki et al. (2016b) [15]. AFM1 transfer (11.4 and 0.00 ± 1.60 g/kg), excretion (29.52 and 0.00 ± 4.58 μg/d), and concentrations in milk (0.76 and 0.00 ± 0.16 μg/kg) were greater for POS than CON, respectively, however, other treatments showed no differences. A tendency for a quadratic treatment effect (p = 0.08) was observed for fecal AFB1 concentrations at 6.78, 8.55, and 5.07 ± 1.17 μg/kg for the POS, P30, and P60 treatments, respectively. The authors described a quadratic treatment effect ($p < 0.001$) for plasma superoxide dismutase (SOD) concentrations at 2.77, 1.99, and 1.97 ± 0.05 U/mL for POS, AD30, and AD60 treatments, respectively. According to the conclusions of the paper, the addition of a yeast and bentonite clay-based adsorbent altered blood metabolites and decreased AFB1 concentration in the feces. The adsorbents had no effect on cow feed intake and milk production or efficiency and aflatoxin transfer or excretion, however, they were beneficial in the reduction of inflammation during aflatoxin challenge.

4.2. Plant-based adsorbents

Ramales-Valderrama et al. (2016) [26] studied the in vitro adsorption of AFB1 and AFB2 from aqueous solutions using *Pyracantha koidzumii* biomasses (leaves, berries and the mixture of leaves/berries). The biosorbent was used at 0.5% (w/v) in samples spiked with 100 ng/mL of B-aflatoxin standards and incubated at 40°C for up to 24 h. The experiment was conducted as a completely randomized 3 x 5 factorial design, fifteen experimental conditions were carried out with three replicates. A standard biosorption methodology was used. The aflatoxins were quantified by an immunoaffinity column and UPLC methodologies. According to the results, biosorption capacity of the biomasses increased with incubation time and reached the maximum at 6 h. Subsequently, adsorption capacity did not change significantly up to 24 h. Biosorption percentages were the highest in case of biomasses from leaves alone and leaves/berries mixture, 86% and 82%, with final aflatoxin concentrations of 13.87 and 18.07 ng/mL, respectively (6 h). Biomass of berries showed moderate aflatoxin adsorption capacity (46%). According to the analysis of zeta (or electrokinetic) potential, the authors concluded that the interaction type between aflatoxin and the biosorbent is primarily electrostatic. According to the Fourier-transform infrared spectroscopy (FTIR) analysis, hydroxyl, amine, carboxyl, amide, phosphate and

ketone groups are likely responsible for the biosorption of AFB molecules. The authors concluded that their study demonstrated that conventional biomasses from *P. koidzumii* have good potential for the biosorption of AF from aqueous solutions being a natural, abundant, cost-effective and environmentally friendly biomaterial, however, the high in vitro binding capacity of biosorbents has to be further tested with in vivo experiments as well.

Naseer et al. (2018) [27] investigated the antifungal effects of methanolic extracts of garlic (*Allium sativum* L.), clove (*Syzygium aromaticum*) and neem (*Azadirachta indica*) against *Aspergillus* species. The prevalence of different fungal species in calf feed was also investigated. The plants were dried, powered grind and then extracted three times with boiled methanol. The extracts were concentrated with rotary evaporator [28]. Well diffusion method [29] was used for the evaluation of antifungal activity of methanolic extracts. The results showed antifungal activity for all plants, with maximum zone of inhibition against *A. flavus* and *A. parasiticus* was shown by garlic extract (16.5 mm) followed by clove (13.04 mm) and neem (9.06 mm). It was concluded that these extracts may be used for control of mycotoxin intoxication of concentrate feed used for calf rearing. According to the results of feed sample analyses (n = 74), out of mycotoxins contaminated concentrate feed samples, the highest frequency of *Aspergillus* (43.3%) was observed. Out of 29 *Aspergilli*, maximum frequency (72.4%) of *A. flavus* was recorded followed by *A. parasiticus* (13.7%), *A. fumigates* (6.8%) and *A. niger* (6.8%). Out of total 74 concentrate feed samples collected, 67 samples had > 20 ppb of aflatoxin B1.

Aflatoxin absorption capacity of Milk Thistle (MT) (*Silybum marianum*) seeds was studied in an in vitro model mimicking the conditions characteristic the stomachs and the intestinal tracts of chickens by Fani-Makki et al. (2018) [30]. Forty-eight flasks, each containing 25 g of rice and 250 or 500 µg/kg AFB1 in the presence of 125, or 250 mg MT seeds were used in six treatments with two samples and four replicates. The lowest levels of AFB1, 45.62 ± 4.25 µg/kg, were determined in the treatment with 250 mg of MT seeds plus 250 µg/kg AFB1. The authors concluded that MT seeds were able to effectively diminish AFB1 *in vitro*, but also noted that the mechanism by which MT seeds decreases AFB1 is not fully understood. The presence of fiber in the seeds acting as adsorbents, silymarin, a natural polyphenolic flavonoid and polyunsaturated fatty acids may also contribute to the beneficial characteristics regarding aflatoxin diminishing.

4.3. Toxin-binding premixes (combination of miscellaneous type feed additives)

4.3.1. Studies evaluating a single toxin-binding premix

In the scientific opinion prepared by European Food Safety Authority EFSA Panel on Additives and Products or Substances used in Animal Feed (FEEDAP Panel) (Rychen et al. 2016) [31], the safety and efficacy of algae interspaced bentonite as a feed additive when used as aflatoxin B1 binder for all animal species was assessed. For the preparation of the opinion, FEEDAP Panel used the data provided by the applicant together with data from peer-reviewed scientific papers and other scientific reports. According to the publication, the additive is considered safe at the maximum recommended dose of 125 mg additive/kg complete feed for the three target species examined (weaned piglets, chickens for fattening and dairy cows). The authors extrapolated this conclusion to all animal species. Regarding efficacy, based on an in vitro experiment, the additive adsorbed 70 to 99% of AFB1 at pH 3 and 68 to 93% at pH 7 and 8 at different toxin concentrations. Experimental values for maximum adsorption were approximately 100 mg AFB1/g additive independent of pH. However, there were no adequate studies that could confirm these effects *in vivo*, therefore FEEDAP Panel could not conclude on the efficacy of the additive.

The objective of the study of Xiong et al. (2018) [32] was to evaluate the effects of a proprietary additive (Solis Mos), containing sodium montmorillonite, live yeast, yeast culture, mannan oligosaccharide and vitamin E, on AFM1 content, lactation performance, plasma biochemical parameters and ruminal fermentation on dairy cows exposed to long-

term AFB1 challenge. Forty early- to mid-lactation multiparous Holstein cows were included in the study which lasted 9 weeks. The animals were grouped according to days in milk and milk production and randomly assigned to four treatment groups: control (CON, no additive, and no aflatoxin), an AFB1-contaminated diet (AF) with addition of AFB1 at
20 μg/kg of dry matter, Solis Mos diet (SM) with addition of Solis Mos at 0.25% dry matter; and a mixed diet (MIX) containing AF and SM. Dry matter intake, milk yield and milk composition were measured on day 6 and 7 each week while milk AFM1, plasma biochemical parameters and ruminal fermentation variables were analysed on the last days of week 1 and week 9. No differences were shown in dry matter intake, milk yield, percentages of milk protein, milk fat and lactose and somatic cell counts. AFM1 concentrations in milk and AFM1 excretion decreased significantly ($p < 0.01$) when adding Solis Mos adsorbent to the diet, by 35.2 and 31.6%, respectively. Aflatoxin transfer from diet to milk also decreased significantly, by 35.5%, and the transfer rate (carry-over) averaged 1.38 and 0.89% for AF and MIX treatments, respectively.

In the study of Xiong et al. (2015) [33], transfer of aflatoxin from feed to milk and the effects of a proprietary feed additive, Solis Mos (SM, a mixture of sodium montmorillonite with live yeast, yeast culture, mannan oligosaccharide, and vitamin E) on AFM1 concentration, plasma biochemical parameters, and ruminal fermentation of dairy cows fed varying doses of aflatoxin B1 (AFB1) were investigated. 24 multiparous Holstein cows in late lactation were assigned to 3 experiments. Cows received no aflatoxin, 20 μg of AFB1/kg of dry matter and 40 μg of AFB1/kg of dry matter in experiment 1, 2 and 3, respectively. In each experiment, there was a control group (CON) and a group receiving 0.25% SM of the diet dry matter (SM). After a 4-day adaptation period, aflatoxin challenge lasted from day 5 to day 11, followed by a 5-day clearance period. AFM1 transfer and secretion were calculated with the following equations:

$$\text{Secretion (ng/d)} = \text{concentration of AFM1 in milk (ng/L)} \times \text{milk yield (kg/d)}, \quad (2)$$

$$\text{Transfer (\%)} = \text{excretion of AFM1 (ng/d)/ AFB1 consumption (ng/d)} \times 100\% \quad (3)$$

equ. 2 [33]

According to the results, there was a 16.0%, 18.3% and 17.9% reduction ($p < 0.05$) in milk AFM1 concentration, secretion, and transfer, respectively, when adding SM to the 20 μg/kg AFB1 contaminated diet on the final day of dosing AFB1 (day 11). However, no effect was detected when the adsorbent was added to the diet containing higher level of AFB1. According to the authors, higher dosage of adsorbent might be needed for the reduction of AFM1 in case of higher contamination levels. These results indicated also that adding SM to diets containing 0 or 20 μg of AFB1/kg decreased milk AFM1 concentration, improved antioxidative status, and altered rumen fermentation, whereas adding SM to a diet containing 40 μg of AFB1/kg did not reduce AFB1 transfer but increased the antioxidant status of the liver.

The aim of the study of Jovaisiene et. al. (2016) [34] was to investigate the effects of long-term exposure to mycotoxins produced by *Fusarium* and *Aspergillus* species on milk composition and biochemical, hematological, immunological parameters of dairy cows and to determine the anti-mycotoxin effect of the additive Mycofix Plus 3.E (mineral components, biological constituent, live organism, phytogenic substances, phycophytic constituents). In the experiments, feeds were naturally contaminated with mycotoxins: silage, haylage, hay, barley. Zearalenon (ZEA) was the major contaminant found in the hay at levels of up to 1000.0 μg/kg of dry matter. Deoxynivalenol (DON) was another contaminant found in the hay at levels of up to 600.0 μg/kg of dry matter. The highest AFB1 concentration, 10.0 μg/kg, was found in ground barley. 28 Lithuanian red cows were separated into two groups (14–14 animals). Control group (CG) received naturally contaminated diet while experimental group (EG) received naturally contaminated diet + 40 g per day of cow Mycofix Plus 3.E. According to the results, milk production increased

by 6.1% in CG and by 12.0% in EG after 63 days. The results of the blood haematological analyses suggest that most of the examined parameters were not affected by mycotoxin contaminated diet and mycotoxins plus feed additive. Serum biochemistry data show a change from physiological parameters in case of urea, which was higher in CG. A non-significant change was shown immunity status, IgA concentrations were decreased in CG and increased in EG after 63 days. The authors concluded that Mycofix Plus 3.E can prevent physiological effects caused by low concentrations of long-term mycotoxin exposure of dairy cows.

4.3.2. Studies comparing different toxin-binding premixes

Aslam et al. (2016) [35] studied the extent of transfer of aflatoxin B1 in feed to the aflatoxin M1 metabolite in milk (carry-over) in Nili-Ravi buffaloes and also the efficacy of a commercial mycotoxin binder incorporated into feed. The mycotoxin binder used was a 50% – 50% mixture of commercial available products Mycofix Secure and Mycofix Plus of BIOMIN (Getzersdorf, Lower Austria, Austria). Mycofix Secure is composed of 100% of bentonite/dioctahedral montmorillonite. Mycofix Plus is composed of bentonite/dioctahedral montmorillonite, *Biomin BBSH 797* (*Gen. sp. nov. nov.*, formerly *Eubacterium*), *Biomin MTV* (*Trichosporon mycotoxinivorans* DSM 14153), phytophytic (*Ascophyllum nodosum*) and phytogenic (silymarin) substances. Multiparous animals (n = 28) were randomly distributed to four groups corresponding to two treatments each with two levels of aflatoxin B1. Animals were exposed to naturally contaminated feed providing a total of 1475 mg/day (Groups A and B) or 2950 mg/day (Groups C and D) of aflatoxin B1. Groups B and D were given 50 g of mycotoxin binder daily mixed with feed whereas Groups A and C were kept as controls. The mean value of total daily aflatoxin M1 excretion for Groups C and D (112.6 mg/day) was almost double than the excretion in Groups A and D (62.2 mg/day). The mean daily concentration of aflatoxin M1 in milk of animals from both treatment groups supplemented with 50 g/day of mycotoxin binder was 76.5 mg/day, nearly 22 mg lower than those without binder at 98.3 mg/day. The 50 g/day of binder was able to sequester aflatoxin B1 with the same efficiency in groups fed with high and low concentrations of aflatoxin B1. Carry over was lower in animals supplemented with 50 g/day of mycotoxin binder (3.44%) than those fed no binder (4.60%). According to the results, the examined mycotoxin binder is able to decrease the transfer rate of aflatoxin B1 to aflatoxin M1 but not able to completely exclude the risk of milk contamination.

In the study of Naveed et al. (2018) [36], carry-over rate of aflatoxin B1 to aflatoxin M1 and the effects of three toxin binders, Fixar Viva, Mycosorb, T5X were investigated in Nili-ravi buffaloes (n = 16). The animals were divided into four treatment groups, three of them were receiving the three different toxin binders added at concentration of 0.25% of dry matter intake of animal, and one was a positive control group. All of them were fed with 500 μg/kg of aflatoxin B1 for 28 days after a 6-day pre-experimental phase for depletion of potential mycotoxin residues. According to the results, the carry-over rate was 2.13%. Addition of three toxin binders Mycosorb, Fixar Viva, and T5X at a concentration of 0.25% in ration resulted in 47%, 39%, and 35% reduction in AFM1 secretion, respectively. According to the findings of the study, percentage carry-over of AFM1 in buffaloes is higher than that reported in lactating cows as well as in goats and Mycosorb can reduce the excretion of AFM1 into milk by improving the dry matter intake, milk production and protein contents.

Ullah et al. (2016) [37] compared the toxin-binding efficacy of two binders, Toxfin (Sepiolite, bentonite and companion clays) and Elitox (enzymes, hydrated sodium calcium aluminosilicates (HSCAS), biopolymers, vitamin C and natural extracts), in an in vivo experiment on 32 Beetal goats, in terms of reducing excretion of AFM1, total viable bacterial count and somatic cell count in milk obtained from goats fed with AFB1 contaminated diet. The animals were divided into 4 equal groups (A. control, B. fed with 40 μg/kg AFB1, C. fed with 40 μg/kg AFB1 along with Toxfin at 3 g/kg cotton seed cake,

D. fed with 40 μg/kg AFB1 along with Elitox at a dose rate of 1 g/kg of cotton seed cake for 7 days, respectively). According to the results, Toxfin significantly reduced the excretion of AFM1 (49.57% decrease), while Elitox caused a non-significant (19.49% decrease). There was no significant reduction in total viable cell count in milk samples of group C and D and somatic cell count in group C, however, somatic cell count in milk samples of group D decreased significantly.

In the study of Ogunade et al. (2016) [38], the effects of three sequestering agents (SEQ); proprietary mixture of *Saccharomyces cerevisiae* fermentation product containing a low (SEQ1) or high (SEQ2) dose of a chlorophyll-based additive, or a low dose of the chlorophyll-based additive and sodium bentonite clay (SEQ3) were investigated in terms of AFM1 reduction, performance, and immune status of dairy cows. Fifteen lactating animals were used in the experiment with an incomplete crossover design including four 28-d periods. Treatments were the following: a control diet (C), a toxin diet (T; 1,725 μg of AFB1/head per day; 75 μg/kg), and diets containing the toxin and 20 g/head per day of SEQ1, SEQ2 and SEQ3. The AFB1 was mixed with ground corn and molasses, weighed into gelatine capsules, and dosed orally to cows before the total mix ration (TMR) was fed on d 21 to 25 (First 20-day period was for adaptation, clearance was monitored from d 26 to d 28). Sequestering agents were top-dressed on the total mixed ration (TMR) daily in each period. Milk was sampled twice daily on days 20 to 28 and plasma was sampled on d 20 and 25. Aflatoxin M1 excretion was calculated as the product of milk AFM1 concentration and milk yield. Transfer of the toxin to milk during the dosing period was calculated as daily AFM1 excretion/daily AFB1 intake. Transfer of the toxin during the withdrawal period was calculated as daily AFM1 excretion/AFB1 intake on d 25. Aflatoxin M1 clearance rates were calculated by dividing the difference between the AFM1 concentration on d 26, 27, and 28 and that on d 25 by 24, 48, and 72 h, respectively. The authors detected no AFM1 in the milk of cows fed diet C, whereas cows fed diets T, SEQ1, SEQ2, and SEQ3 had mean milk AFM1 concentrations of 0.73, 0.74, 0.73, and 0.74 μg/kg, respectively. Adding sequestering agents to diets containing the toxin did not affect milk AFM1 concentration, excretion, or transfer during the toxin-dosing period. Therefore, none of the sequestering agent treatments prevented milk AFM1 concentrations from rising beyond the FDA action level (0.5 μg/kg). These results suggest that the active ingredient amounts in the sequestering agent mixtures were inadequate for toxin binding. However, when AFB1 was withdrawn from the diet, AFM1 concentrations decreased rapidly in SEQ groups, such that they fell below the FDA action level within 24 h, whereas it took 48 h in case of group T. Sequestering agents examined were able to modulate immune response. Regarding immune status, SEQ1, which had the greatest proportion of the *S. cerevisiae* fermentation product, had the greatest beneficial effects. SEQ2, which had the greatest proportion of the chlorophyll-based additive, had the least beneficial effect on immune response, suggesting that the additive may have been less effective at binding the aflatoxin or modulating the immune response than the *S. cerevisiae* fermentation product or the clay according to the authors' conclusions.

5. Microbes and enzymes

5.1. Yeasts (Saccharomyces and others)

In the study of Dogi et al. (2017) [39], the effects of probiotic *Saccharomyces cerevisiae* RC016 on morphometric parameters (villus length, width, and crypt depth) and goblet cells of rats exposed to aflatoxins for 60 days were studied. An in vitro experiment for investigating the effect of AFB1 exposure on yeast cell ultrastructure, diameter and cell wall thickness has also been conducted. A total of 36 male Wistar rats were divided into six different groups (n = 6): (T1) control diet + 0.2 mL 0.85% NaCl orally administered; (T2) control diet + of *S. cerevisiae* (2×10^8 viable cells in 0.85% NaCl) orally administered; (T3) experimental diet 1 + 0.2 mL 0.85% NaCl orally administered; (T4) experimental diet 2 + 0.2 mL 0.85% NaCl orally administered; (T5) experimental diet 1 + *S. cerevisiae* (2×10^8 viable

cells in 0.85% NaCl) orally administered; and (T6) experimental diet 2 + *S. cerevisiae* (2×10^8 viable cells in 0.85% NaCl). Yeast strain and NaCl were administered by gavage. Animals were fed 30 g of feed per day and consumed water *ad libitum*. According to the results, there were no differences in villus width and an increase in villus length and crypt depth in T2 compared to the control group occurred. Unexpectedly, AF-contaminated diets (T3 and T4) also resulted in increased villus length, width and crypt depth compared to T1. However, yeast and AFs did not show accumulative effect when both were administered. In T6, crypt depth was reduced, possibly due to the AF-binding to the yeast cell wall. There was no significant difference in the number of goblet cells between T2 and the control group, whereas AF-treated groups showed significantly lower cell counts ($p < 0.05$). In T6, the probiotic yeast increased the goblet cell count to the level of the control group.

In the in vitro experiment, *S. cerevisiae* RC016 cells were exposed to 20 μg/mL of AFB1 in intestinal solutions or in phosphate buffered saline and cells processed for transmission electron microscopy and high-resolution light microscopy studies. According to the results, a significant increase in the yeast cell diameter was observed when RC016 was exposed to aflatoxins, suggesting this as an advantage since a larger cell would be able to adsorb mycotoxins more efficiently. The authors concluded that the ability of this strain to act as probiotic and aflatoxin binder makes it a candidate for the formulation of new additives to improve animal performance.

González Pereyra et al. (2014) [40] investigated the safety-in-use of the probiotic *Saccharomyces cerevisiae* RC016 and tested its ability to reduce genotoxicity caused by dietary aflatoxins (AFs) in a 60-days experiment. Altogether 36 inbred male Wistar rats (*Rattus norvegicus*) were divided into 6 experimental groups: (1) Uncontaminated feed control, (2) yeast control, (3) diet 1 (40 μg/kg AFB1 + 20 μg/kg AFG1), (4) diet 2 (100 μg/kg AFB1 + 50 μg/kg AFG1), (5) diet 1 + daily oral dose of 10^8 viable S. *cerevisiae* cells, and (6) diet 2 + daily oral dose of 10^8 viable *S. cerevisiae* cells. AFB1 consumption was approximately
1.2 and 3.0 μg for animals receiving diets 1 and 2, respectively. Daily AFG1 consumption for each diet was 0.6 and 1.5 μg, respectively. Rat erythrocytes and lymphocytes was evaluated through the bone marrow rodent micronuclei assay and the comet assay, respectively, to evaluate the genotoxic effects. The induction of micronuclei in each experimental group was reported as the mean occurrence of micronucleated polychromatic erythrocytes (MNPCE) and micronucleated normochromatic erythrocytes (MNNCE). According to the results, *S. cerevisiae* RC016 in itself did not cause any genotoxicity or cytotoxicity. However, AF-contaminated diets caused genotoxicity in groups 3, 4 and 5. The administration of *S. cerevisiae* RC016 was able to reduce the genotoxicity index in group 6 (0.0189) compared with group 4 (0.0376) within animals consuming the same level of toxin ($p \leq 0.05$). The genotoxicity caused by AFs in group 6 was reduced to the level of the control group, showing no statistical difference when comparing MNPCE% (percentage of MNNCE over total erythrocytes) with groups 1 and 2. In this experiment, AFs did not cause cytotoxicity at the levels tested. According to the results of the comet assay, there were no significant difference in the mean values of tail moment (TM) used for the estimation of DNA damage. The yeast did not cause any impairment on the examined health parameters of the animals (weight and feed parameters, general health status, status of the organs). Moreover, *S. cerevisiae* RC016 improved zootechnical parameters in AFs treated animals. In summary, the beneficial effects of the administration of the yeast strain to animals were more expressed in the groups treated with both *S. cerevisiae* and AFs, compared to the untreated control group and the yeast-only treated groups.

The authors concluded that *S. cerevisiae* RC016 administration cannot be associated with genotoxicity and cytotoxicity, and its probiotic and AF-binding characteristics make it a great candidate for feed additive development.

The aflatoxin B1 binding ability of native yeast strains isolated from broilers' environment as feedstuff, faeces and gut was studied in the publication of Magnoli et al. (2016) [41]. A total of 9 yeast strains were isolated, from which five were selected to carry out the adsorption studies; *Clavispora lusitaniae* and *Pichia kudriavzevii* from feedstuff, *Cl. lusitaniae* and *Cyberlindnera fabianii* from faeces and *Candida tropicalis* from gut. According to the results, the binding level increased with increasing cell concentrations, and the adsorption capacity of the tested strains increased with increasing toxin concentrations. The most appropriate concentrations for cells and toxin were 10^7 cells/mL and 100 ng/mL of AFB1, respectively. All yeast strains showed similar adsorption capacities. The highest AFB1 affinity was observed for *Cl. lusitaniae* from feedstuff (0.032 ± 0.004 mol/l). The lowest value was observed for *P. kudriavzevii* from feedstuff (0.005 ± 0.002 mol/l). *Cy. fabianii* isolated from faeces and *Ca. tropicalis* isolated from the gut showed moderate affinity for the toxin (0.011 ± 0.001 mol/L and 0.017 ± 0.009 mol/L, respectively). The two highest values of surface excess were observed for *P. kudriavzevii* from feedstuff (19.9 ± 0.7 mg/g), while the lowest values were observed for *Cy. fabianii* (3.9 ± 0.0 mg/g) and *Cl. lusitaniae* (6.3 ± 2.0 mg/g) from faeces and Ca. *tropicalis* (4.5 ± 0.1 mg/g) from the gut (Table S2). The adsorption isotherm studies showed behaviour of L type Langmuir and varied affinity for the toxin, indicating a finite number of equivalent adsorption sites on the absorbent surface. An irreversible binding process of the AFB1-yeast complex was demonstrated regarding the stability studies. According to the authors conclusion, the tested yeast strains are potential AFB1 adsorbents with the advantage of the native avian environment for broiler production.

Table S2. Results of Magnoli et al. (2016) [41] "Aflatoxin B1 (AFB1) binding percentage (%) in different yeast strains (cells per ml) and at different toxin concentrations (ng ml $^{-1}$)".

Source	Yeast Strain	Concentration (cells per ml)	AFB$_1$ (ng ml^{-1})			
			50		100	
			Binding Level			
			%	ng ml^{-1}	%	ng ml^{-1}
**Faeces**	***Clavispora lusitaniae***	10^5	12.95	3.41 ± 1.12^{ac}	16.70	6.51 ± 1.98^{a}
		10^6	14.49	4.71 ± 1.11^{b}	19.91	9.81 ± 1.90^{b}
		10^7	15.09	5.51 ± 1.11^{c}	25.72	15.12 ± 0.42^{c}
	Cyberlindnera fabianii	10^5	12.50	3.32 ± 0.98^{a}	16.89	7.10 ± 0.70^{a}
		10^6	15.89	6.00 ± 1.13^{b}	22.34	12.51 ± 0.34^{b}
		10^7	20.12	10.1 ± 1.11^{c}	25.41	18.22 ± 0.69^{c}
Feedstuff	*Cl. lusitaniae*	10^5	12.26	3.30 ± 0.73^{a}	25.95	15.23 ± 1.40^{a}
		10^6	14.70	4.51 ± 0.11^{b}	29.37	19.12 ± 0.64^{b}
		10^7	15.55	5.22 ± 1.58^{c}	30.05	20.11 ± 0.24^{c}
	Pichia kudriavzevii	10^5	12.57	3.43 ± 1.23^{ac}	22.99	12.88 ± 1.71^{a}
		10^6	13.99	3.81 ± 1.15^{ab}	25.94	15.98 ± 1.45^{b}
		10^7	20.86	10.11 ± 1.21^{c}	30.62	20.68 ± 1.27^{c}
Gut	*Candida tropicalis*	10^5	12.95	2.45 ± 1.12^{a}	18.56	11.88 ± 1.31^{a}
		10^6	13.65	3.50 ± 1.11^{b}	22.98	14.98 ± 1.25^{b}
		10^7	20.65	9.10 ± 0.98^{c}	28.90	19.58 ± 1.01^{c}

Mean ± SD (standard deviation) n = 3.

Values with the same letter are not significantly different according to Fisher's protected least significant test ($p < 0.0001$). Statistical analysis compared the means obtained from each yeast strain separately.

In the study of Poloni et al. (2015) [42], the potentiation of the adsorbent effect for AFB1 of a commercial additive (CA) of animal feed, containing inactive lysate of three *Saccharomyces cerevisiae* strains, active enzymes, adsorbents, and a selenium–amino acid complex, when the additive was mixed separately with three *S. cerevisiae* strains (RC009,

RC012 and RC016). The binding capacity of CA concentrations of 50, 75 and 100% alone and in the presence of the yeast strains was evaluated at 20 and 50 ng/g AFB1 levels. Toxin desorption under simulated gastrointestinal conditions was also evaluated. According to viability studies, none of the CA concentrations affected the yeast strains. According to the results, there were no significant differences in the effectiveness of the tested mixtures between CA and RC009 at reducing AFB1 levels at different concentrations of CA and AFB1, the efficiency increased with decreasing percentage of CA. Desorption rates of AFB1 reached 35% and 28% with 20 ng/g and 50 ng/g AFB1, respectively.

The tested mixture between CA and RC012 was effective at 50 ng/g AFB1 and adsorption rates increased as the concentration of CA increased. Desorption rates were lower than those of the mixture CA–*S. cerevisiae* RC009, reaching 18.8% and 5.6% with 20 ng/g and 50 ng/g of AFB1, respectively.

The tested mixture between CA and RC016 had adsorption rates between 51.2% and 97.5% with 20 ng/g AFB1 and desorption percentages between 6.3% and 8.0%. Adsorption increased with increasing CA concentrations. However, with 50 ng/g AFB1 there were no differences in adsorption to the different percentages of CA used and the effectiveness was significantly lower.

Overall, the mixtures CA-RC012 (at 50 ng/g AFB1) and CA-RC016 (at 20 ng/g AFB1) were the most efficient in their capacity to adsorb AFB1 ($p < 0.0001$). In the current study, CA alone had an adsorption capacity under 50%, and higher efficiency could have been obtained when combining the CA with *S. cerevisiae* strains (RC012 and RC016). The authors concluded that CA combined with *S. cerevisiae* RC012 or RC016 at different conditions was highly efficient in AFB1 adsorption. However, they did not show a similar behaviour between adsorption percentages and mycotoxin concentrations. The explanation might be that the AFB1 adsorption is dependent from the yeast strain. Regarding AFB1 desorption, the authors concluded that toxin binding is a reversible process and in case of the mixtures, it increased with increasing CA concentration. In summary, addition of live RC012 and RC016 strains improve AFB1 binding capacity when added to CA, and besides, their probiotic characteristic also supplies beneficial effects to the animals.

The study of Gonçalves et al. (2017) [43] evaluated the effects of different sources of *Saccharomyces cerevisiae* biomass (20.0 g/d, containing 8.0×10^{10} cells-equivalent per gram) on milk yield, fat and protein proportion and aflatoxin excretion in milk from dairy cows. S. *cerevisiae* was obtained from sugarcane and the types were cell wall (CW), dried yeast (DY), autolyzed yeast (AY) and brewery yeast (BY, partially dehydrated, from beer industry). The administered dose of aflatoxin was 480 μg aflatoxin B1 per day from treatment day 1 to 6. 20 dairy cows were involved in 10 dietary treatments (2 cows per treatment): (1) negative control (no AFB1 or *S. cerevisiae*), (2) positive control (AFB1), (3-4) CW alone; CW + AFB1 (5-6) AY alone; AY + AFB1, (7-8) DY alone; DY + AFB1, (9-10) BY alone; BY + AFB1. The S. *cerevisiae*-based products were administered orally in capsules immediately after the morning and afternoon milking, for 7 consecutive days, starting on d 4 of the experiment. The experiment was conducted for 10 consecutive days, with 3 initial days of dairy cows' exposure to AFB1 alone, followed by 3 days of simultaneous exposure to AFB1 and administration of S. *cerevisiae*-based products, and finally 4 days with *S. cerevisiae*-based products alone. According to the results, there were no changes observed in the lactation performance (milk production, fat and protein content) or general health status of the animals. Aflatoxin M1 levels found in milk (μg/L) on each sampling day were multiplied by the milk volume produced (L/d) to obtain the total amount of AFM1 excreted daily into milk (μg/d). This value was used for the calculation of the percentage of carry-over rate:

$$\text{Carry-over (\%)} = \text{excretion of [total AFM1 excreted (μg/d)/AFB1 ingested (μg/d)]} \times 100 \quad \text{equation 3 [43].} \quad (4)$$

Aflatoxin M1 was detected in the milk from all aflatoxin treatment group cows, reaching maximum levels at day 3 and varying from 0.52 ± 0.03 to 1.00 ± 0.04 μg/L. The

maximum carry-over value (3.95%) was found in cows from AY treatment on day 3 of the experimental period. The lowest value (0.2%) was obtained on d 7 to 10 for AY treatment. The CW and AY treatments had the lowest carry-over values ($p < 0.05$), compared with the other treatments, starting on d 4 and 5, respectively. The DY and BY treatments also had milk with lower carryover percentages compared with the positive controls. At the end of the treatment period, CW, AY, DY, and BY removed 78%, 89%, 45%, and 50% of AFM1 from the milk, respectively, compared to the highest level found on day 3. The authors concluded that results indicate a potential application of industrial fermentation by-products, especially CW and AY, as a feed additive in the diets of dairy cows to reduce the excretion of AFM1 in milk.

In the study of Tayel et al. (2013) [44], the aim was to evaluate *Pichia anomala* ATCC 34080 strain as a biocontrol agent against *A. flavus* contamination in corn grains, and to explore the antifungal mode of action of this antagonistic yeast as well as its value to increase the protein content as well as the animals' performance after feeding.

In the experiments, the most powerful strain with the heaviest band on TLC plate from *A. flavus* isolates had the ability for aflatoxin production, was chosen as a model for aflatoxigenic fungi.

In the first experiment, in vitro antagonism between the *P. anomala* and the toxigenic *A. flavus*, on Petri dish was studied. It was shown that *P. anomala* could effectively prevent the fungal growth as proved by the clear zone surrounding yeast culture.

The experiment for hydrolytic enzymes secretion indicated that *P. anomala* could produce β-1,
3-glucanase and exo-chitinase in supplemented media with β-1,3- glucan or colloidal chitin, as a sole source of carbon, respectively. The chromogenic determination of enzymes activity indicted that the maximum exo-chitinase activity of 2.16 units was achieved after 72 h of incubation, whereas the maximum activity of β-1, 3-glucanase (4.62 units) was obtained after incubation for 96 h. For both enzymes, the enzymes activity gradually increased to their maximum levels and then decreased with the prolongation of incubation period. By scanning electron micrographs of cultured *P. anomala* with fungal hypha, it was revealed, that *A. flavus* was colonized by the biocontrol yeast which subsequently led to hyphal lysis and deterioration completely.

For the animal experiments, conducted in duplicates, eight weeks old male CD-1 mice were fed with the experimental diets for 6 weeks, and average weight gain and mortality percentage was determined for each group. According to the results, by supplementing feed with yeast culture, in addition to its growth promotion capability, *P. anomala* could efficiently prevent animals from mortality after feeding with contaminated feed. The best trial, for enhancing fed animals' performance, was the fortification of uncontaminated feed with *P. anomala* at a rate of 3 mg/g; the average weight gain was the highest (68.32%) and no animal death was recorded.

The authors concluded that *P. anomala* could be considered as a perfect model for the biocontrol yeast. The antifungal actions of this yeast could strongly prohibit the growth of aflatoxigenic *A. flavus*. *P. anomala* is recommended by the authors as a valuable protein supplement in feed formulation.

5.2. Lactic acid bacteria for improving silage fermentation

Dogi and co-workers (2015) [45] tested the efficacy of *Lactobacillus rhamnosus* RC007 and *L. plantarum* RC009 on the fermentation quality and mycrobiota of corn silage in mini-silos. They evaluated the incidence of three *Aspergillus* species and their lactic acid production and compared the results with uninoculated silage. At the beginning of the ensiling process, rapid lactic acid production and decline in pH values were seen in silage inoculated with *L. rhamnosus* RC007. From the initial 5.9 log CFU/g the yeast count decreased, on day 127, after aerobic exposure to 5.3 ± 0.3 log CFU/g and the 6.0 log CFU/g mould count decreased to 4.97 ± 0.03 log CFU/g. In comparison, after aerobic exposure, the lactic acid content declined significantly in untreated and *L. plantarum* RC009-

inoculated silages. The yeast count was 6.31 ± 0.02 log CFU/g and the mould count was 5.62 ± 095 log CFU/g on day 127. In the untreated silage yeasts count remained constant throughout the ensiling period. *L. rhamnosus* RC007 was the most efficient at inhibiting the three fungal species.

In the study of Zielinska and Fabiszewska (2018) [46], five bacterial strains were examined in different combinations in microsilos and in farm conditions: *L. buchneri* A KKP 2047 p, *L. reuteri* M KKP 2048 p, *L. plantarum* K KKP 593 p, *L. plantarum* S KKP 2021 p, *L. fermentum* N KKP 2020 p. They used bacterial inoculants (10 g/t of maize grain) before ensiling. The bacterial strains had synergistic effect and decreased the aflatoxin B1 levels by about 80% compared to control silage and by about 74% compared to silage inoculated with only one strain (*L. buchneri* A KKP 2047 p). Among the different combinations, silages inoculated with five bacterial strains resulted in the lowest yeast and mould count values. Studies relating to the synergistic activity of bacterial strains were also conducted on production scale in the following schemes: all five bacterial strains, one *L. buchneri* strain, negative control, no inoculants added. The production scale experiment confirmed the synergistic activity of the five bacterial strains. It resulted in a decrease in mould count and a decrease in aflatoxin B1 levels in silages by 74 and 75% respectively compared to negative control and by 65 and 67% respectively relative to *L. buchneri* control. Using the five bacterial strain composition, maize grain silages had high stability, microbiological and chemical purity therefore safe to use in feeding even after 3 years of preservation.

In the study of Ying et al. (2017) [47], two additives (bacterial inoculants and fibrolytic enzymes) were tested for their effects on fermentation characteristics, in vitro digestibility, mould counts and AFB1 and total aflatoxins (B1, B2, G1 and G2) content in corn silages. Reduction of AF-production in silage during exposure to air was also investigated.

Whole-plant corn was harvested with one-half milk line stage and ensiled after the following treatments: 1. untreated (CON); 2. bacterial inoculants (*Lactobacillus rhamnosus* > 6 $x10^{10}$ CFU/g) at 10^5 CFU/g of fresh forage (B); 3. fibrolytic enzymes (cellulase, glucosidase, avicelase and xylanase, 30 000 U/g) at 100 U/g of fresh forage (E); and 4. mixture of bacterial inoculants and enzymes at 10^5 CFU/g and 100 U/g of fresh forage, respectively (B+E).

According to the results, all treated silages had higher lactic acid and acetic acid contents ($p < 0.05$) than CON. Only B had higher lactic/acetic acid ratios than CON. All treatments decreased the NH3-N content ($p < 0.05$). E and B+E decreased the neutral detergent fiber (NDF) content ($p < 0.05$), and increased dry matter (DM) and crude protein (CP) content ($p < 0.05$). E also increased water-soluble carbohydrate (WSC) content ($p < 0.05$). However, there were no significant differences between treated and untreated silage for starch and acid detergent fiber (ADF) content ($p < 0.05$). Compared with CON, all the additives increased in vitro dry matter digestibility (IVDMD) ($p < 0.05$). E and B+E also increased in vitro CP digestibility (IVCPD) ($p < 0.05$) and in vitro NDF digestibility (IVNDFD) ($p < 0.05$). However, none of the additives influenced in vitro ADF digestibility (IVADFD) ($p < 0.05$).

During aerobic exposure, untreated silage had a temperature rise of more than 2°C above the ambient after 116 h. Silages treated with B, E and B+E had a temperature rise of more than 2°C above the ambient after 228, 162 and 294 h respectively.

All treatments increased silage aerobic stability compared with untreated silages ($p < 0.05$). The total AFs and AFB1 contents and mould counts of the silages after exposure to air are shown in Table S3. The mould counts in the treated silages were lower than CON throughout the period of air exposure ($p < 0.05$). At the beginning of the treatment, all the additives, especially B and B+E, had lower total AFs and AFB1 contents than CON ($p < 0.05$). Lower total AFs and AFB1 levels were also detected in the treated silages compared with CON after 1, 5 and 10 days exposure to air ($p < 0.05$). The total AFs and AFB1 contents of the B+E silages were not detected throughout the air exposure stages.

Table S3. Results of Ying et al. (2017) [47] "Aerobic stability and changes in aflatoxin (AF) contents and mold counts for the silages treated with or without additives after air exposure".

	CON		B		E		B+E		
Item	Mean	SE	Mean	SE	Mean	SE	Mean	SE	*p* value
Aerobic stability (h)	116[d]	1.68	228[b]	1.45	1.62[c]	1.07	294[a]	1.48	<0.001
Molds (lg CFU g^{-1})									
Air exposure (days)									
0	1.89[a]	0.08	1.05[c]	0.04	1.46[b]	0.09	0.55[c]	0.07	<0.001
1	2.24[a]	0.09	1.76[c]	0.15	2.05[b]	0.13	0.84[d]	0.11	<0.001
5	3.78[a]	0.05	2.12[c]	0.06	2.63[b]	0.03	1.02[d]	0.01	<0.001
10	4.69[a]	0.06	3.41[b]	0.14	3.55[b]	0.03	0.51[c]	0.18	<0.001
AFs (ng kg^{-1} in DW)									
Air exposure (days)									
0	0.32[a]	0.01	0.00[c]	0.03	0.13[b]	0.02	0.00[c]	0.01	<0.001
1	0.64[a]	0.01	0.15[c]	0.01	0.33[b]	0.03	0.00[d]	0.02	<0.001
5	2.47[a]	0.02	1.08[c]	0.02	1.69[b]	0.03	0.00[d]	0.02	<0.001
10	6.92[a]	0.06	2.98[b]	0.02	3.04[b]	0.03	0.00[c]	0.02	<0.001
AFB1 (ng kg^{-1} in DW)									
Air exposure (days)									
0	0.22[a]	0.01	0.00[c]	0.01	0.07[b]	0.01	0.00[c]	0.01	<0.001
1	0.41[a]	0.01	0.06[c]	0.01	0.21[b]	0.01	0.00[d]	0.01	<0.001
5	2.12[a]	0.03	0.64[c]	0.01	0.95[b]	0.02	0.00[d]	0.01	<0.001
10	4.86[a]	0.04	1.79[b]	0.04	1.81[b]	0.03	0.00[c]	0.01	<0.001

Within each row, means CON, B, E and B+E treatments followed by different letters differ significantly ($p < 0.05$).

In the GLM (General Linear Models) procedure, the effects of additives, day and additives x day were highly significant for all parameters.

B, bacterial inoculants, B+E bacterial inoculants plus fibrolytic enzyme, CON, control, no additive, DM, dry matter, E, fibrolityc enzyme, SE, standard error, n = 4.

The authors concluded that the application of bacterial inoculants, fibrolytic enzymes or a combination of these positively influenced the fermentation process, IVDMD, IVCPD and decreased AF content and mould counts of whole-plant corn silages. In addition, E and B+E decreased the NDF content and B and B+E increased the IVNDFD.

The publication of Ma et al. (2017) [48] describes the results of 5 experiments examining the AFB1 binding characteristics of silage inoculant lactic acid bacteria (LAB) and factors affecting the responses.

Experiments 1 and 2 examined the effects of 10 bacterial strains on AFB1 binding in vitro at 10^6 and 10^9 CFU/mL, respectively. In case of experiment 1, binding of AFB1 was generally low (0 to 4.27%). Only *Lactobacillus plantarum* PT5B bound the AFB1 and the binding capacity was low (4%). When applied at 10^9 CFU/mL, all 10 bacteria bound AFB1 (18 to 33%), but *L. plantarum* R2014 and EQ12, *L. buchneri* R1102, and *Pediococcus acidilactici* R2142 and EQ01 had the greatest capacity (23.9 to 33%).

In case of experiment 3, effects of bacterial viability and the prevailing pH on AFB1 binding was studied. The greatest binding of AFB1 within a bacterium was achieved by dead *L. plantarum* and *L. buchneri* and viable *P. acidilactici* at pH 2.5 (60.5, 66.5, and 56.9%, respectively), whereas dead *P. acidilactici* at pH 8 bound no AFB1 (0%). More AFB1 was bound across pH and viability treatments by *L. plantarum* and *L. buchneri* compared with *P. acidilactici* (28.7 and 30.5 vs. 12.2%, respectively; $P < 0.001$). Binding efficacy generally decreased in a quadratic manner as the acidity of the culture media decreased. Binding of AFB1 was greater by dead versus viable *L. plantarum* and *L. buchneri* (32.6 vs. 24.8% and

43.3 vs. 17.8%, respectively), but viable *P. acidilactici* was more effective than dead *P. acidilactici* (21.92 vs. 2.44%).

In experiment 4, the effects of mild versus severe heat and acid treatments (85 vs. 100°C; pH 2.5 vs. <1) on bacteria viability was studied, as nonviable *L. buchneri* and *L. plantarum* would not be effective silage inoculants. According to the results, all treatments reduced the bacteria population relative to the control treatment considerably. The population of bacteria was reduced from 10^9 to 10^4 CFU/mL by treatment with HCl at pH 2.5 and to 10^2 CFU/mL by 85 or 100°C, whereas acidification at pH <1 eliminated the bacteria.

Experiment 5 studied the effects of the ensiling duration and benign LAB treatments [37 (viable cells) or 85°C (heated cells) or acidification with HCl at pH 2.5 (acid-treated cells)] on AFB1 binding and silage quality during the fermentation of corn forage. Corn forage was ensiled after treatment with only deionized water (control), AFB1 (30 μg/kg of fresh forage), or a mixture of AFB1 and 10^9 CFU/g of each of the treated bacteria. According to the results, application of AFB1 to ensiled corn forage reduced the rate of acidification in the first 3 days of ensiling, increased the rate of dry matter reduction, reduced the crude protein concentration of the silage, and increased its butyric acid and neutral detergent fibre (NDF) concentration relative to the control treatment. Inoculation with bacteria prevented adverse effects of the toxin on silage butyric acid and NDF concentration. The AFB1 concentration of corn forage spiked with the toxin decreased linearly during ensiling across treatments and was reduced to a safe level for feeding within 72 h (from 30 to ≤ 0.35 μg/kg). Inoculation with bacteria did not affect the rate of AFB1 removal from the silage.

Rather et al. (2014) [49] examined the antifungal and biopreservation potentials of *Lactobacillus plantarum* YML007 isolated from Korean kimchi. In the experiment, maize grain was divided into 3 groups,

1. control group (no treatment), 2. organic acid treated maize (2 L/t of organic acid containing a mixture of potassium sorbate and sodium benzoate was added at concentration of 0.1% (w/v)) and 3. 5x concentrated cell-free supernatant of *L. plantarum* YML007 (2 L/t) to investigate the biopreservative efficacy of the bacterium. The moisture and crude fat contents, the aflatoxin level, and the mould count were analyzed in all three groups on the 1st, 15th, and 30th days of storage. Animal health parameters after feeding experiments (duplicate, with 12 healthy young Wister rats divided into 3 groups) have also been investigated in order to study the potential of the bacterium to be applied as feed additive. All groups were stored for 30 days. The antifungal spectrum of the 5x concentrated cell-free supernatant of *L. plantarum* YML007 was analysed using paper disk and agar well diffusion assays and was compared to the antifungal activity of commercially used preservatives potassium sorbate and sodium benzoate (group 2). *Aspergillus niger* KCTC16683, *Fusarium oxysporum* KCTC16909, *A. flavus* KCTC16682, *Saccharomyces cerevisiae* KCTC12106, *Candida albicans* KCTC 17485, and *Pichia membranifaciens* KCTC 7628 were used as indicator organisms.

According to the results, *L. plantarum* YML007 showed stronger antifungal activity against indicator organisms than the commercial preservatives potassium sorbate and sodium benzoate ($p < 0.01$). It is to be noted that *F. oxysporum* was highly resistant to both chemical preservatives; however, it was strongly inhibited by the *L. plantarum* YML007 supernatant.

Regarding the results of biopreservation experiment, aflatoxin production was significantly less in the treated groups ($p < 0.05$), compared to the control. There was almost no aflatoxin production in the acid-treated (2) and *L. plantarum* YML007 supernatant treated (3) maize samples. The average aflatoxin production in group 2 and 3 maize groups did not exceed 5 parts per billion (ppb).

According to the results of feeding experiments, after 30 days, animals fed with control maize showed a smaller gain in body weight, compared with animals fed with *L. plantarum* YML007-treated maize. The total mean body weight achieved by feeding with

L. plantarum YML007-treated maize was 2.95 g, which was 1.1 g more than for control and acid-treated maize.

The authors concluded that *L. plantarum* YML007 is able to prevent mould growth and aflatoxin production on long-term stored cereal grains, and also showed better results in feeding experiments on animal performance, therefore it has a significant potential to be used as an effective bio-preservative to replace chemical preservatives in food and feed.

Dogi et al. (2013) [50] studied *Lactobacillus rhamnosus* RC007 and *L. plantarum* RC009 as silage inoculants not only from the point of view of improvement of silage fermentation, but inhibition activity against *Aspergillus parasiticus* and *Fusarium gramineraum* and mycotoxin (AFB1 and ZEA) production, and the presence of antibiotics resistance gene were also evaluated. The two strains were selected from six LAB strains based on their fermentation characteristics, rapid growth rate and a consequent rapid decrease in pH value of maize extract medium.

Studies on lag phase, growth rate and AFB1 and ZEA production were carried out in vitro under different regimes of water activity (0.95 and 0.99); pH (4 and 6); temperature (25 and 37°C); and oxygen availability (normal and reduced). *L. rhamnosus* RC007 was able to completely inhibit the *F. graminearum* growth at all assayed conditions, while *L. plantarum* RC009 only did it at pH 4. Both *Lactobacillus* strains reduced the *A. parasiticus* growth rate significantly mainly at 0.99 water activity. A decrease in ZEA production was observed as result of Lactobacillus strains – *F. graminearum* interaction; however, the *A. parasiticus* - *L. plantarum* interaction resulted in an increased AFB1 production. *L. rhamnosus* RC007 proved to have no genes for resistance to the tested antibiotics. For studying the antibiotic resistance of the strains, the presence of vanA, vanB, mecA, blaZ and ermB resistance genes was evaluated.

The authors concluded that the ability of *L. rhamnosus* RC007 to inhibit fungal growth and mycotoxin production and also absent in antibiotic resistance genes, therefore it is a potential biocontrol inoculant agent in animal feed.

5.3. Lactic acid bacteria for aflatoxin reduction

Drobná et al. (2017) [51] investigated the antifungal activity and aflatoxin binding potential of new potentially probiotic strains isolated from stomach mucus of lamb, namely, *L. reuteri* E and *L. mucosae* D, *L. murinus* C and, from goatling *L. reuteri* KO5, *L. reuteri* KO4b, *L. reuteri* KO4m, and *L. plantarum* KG1, *L. plantarum* KG4.

Antifungal activity was tested by dual culture overlay assay against seven fungal species (common food contaminants), from which *A. flavus* and *F. culmorum* are mycotoxin producers. Growth inhibition was measured as the reduction area of mycelial growth. All tested *Lactobacillus* strains showed antifungal activity (7.4–43.0% of growth inhibition), most potent were *L. reuteri* CCM 3625, *L. reuteri* KO4b, *L. reuteri* KO5, *L. plantarum* KG4 and *L. mucosae* D. The lowest antifungal activity showed *L. murinus* C. The highest growth inhibition of *A. flavus* was showed by *L. mucosae* D (30.9%). The antifungal activity of the sole cell-free supernatant (CFS) of *Lactobacilli* was determined in the acidic region of pH (pH 3, pH 4 and pH 5) on Sabouraud agar. For the majority of the strains and fungi, the highest inhibition of fungal growth was observed at pH 4. At this pH, strains *L. reuteri* E and *L. reuteri* CCM 3625 were the most active against fungal species.

Impact of culture conditions on antifungal activity of CFS was examined by supplementing glycerol and inactivated *E. coli* to cultivated *L. reuterii* strains (Sabouraud agar, pH 4). According to the results, activity of the individual strains was not significant, except for the activity against *R. oryzae* ($p < 0.01$). Regarding the medium composition on antifungal activity, a significant difference in antifungal activity with and without the aminoacid supplementation (phenylalanine, tyrosine and α-ketoglutarate) was detected against *Aspergillus flavus* and *Rhizopus oryzae*.

Regarding 4-hydroxyphenyllactic acid (4-OHPLA) and 3-phenyllactic acid (PLA) production, all strains, except *L. reuteri* KO4b, produced them. The highest amount of PLA was produced by

L. plantarum KG4 (0.7400 ± 0.0217 mmol/L) and *L. reuteri* E (0.6800 ± 0.0224 mmol/L). The highest production of 4-OHPLA was observed in *L. reuteri* CCM3625 (0.4000 ± 0.0126 mmol/L).

The ability of five isolates to bind AFB1 was tested in vitro. Three strains (*L. reuteri* E, *L. reuteri* KO4b and *L. plantarum* KG4) were selected according to their antifungal activity. Strain *L. mucosae* D was selected for its ability to bind to intestinal mucosa (unpublished results of the authors). *L. reuteri* CCM 3625 was used as a reference strain. Binding activity was evaluated at three time intervals. According to the results, all the analysed lactobacilli were able to bind AFB1 (10.8–66.7%). The extent of binding ability was directly proportional to the length of incubation with AFB1 for all strains with the exception of *L. mucosae* D strain, which showed a decreased activity with time. The best results concerning AFB1 reduction and reproducibility of the reduction process were obtained with *L. reuteri* KO4b strain (66.7% ± 1.0%) followed by *L. plantarum* KG4 (59.4% ± 1.6%) after an incubation of 24 h. The authors concluded that a combination of both the antifungal activity and AFB1 binding activity indicates the potential of the strains as feed supplements as biological conservation agents.

Nasrabadi et al. (2013) [52] investigated the effects of oral administration of *Lactobacillus casei* Shirota (LcS) in aflatoxin-induced rats. In the experiment, twenty-four rats (male Sprague Dawley rats, 7–8 weeks old) were divided into three groups: 1. untreated control under normal diet, 2. AFB1-induced group (25 µg/kg body weight daily for 5 days per week over the next two weeks) and 3. AFB1-induced group with LcS supplementation (10^8 CFU by oral gavage daily for 20 consecutive days). Blood samples were collected on days 18, 19 and 20. The level of blood alanine transaminase (ALT) and aspartate transaminase (AST) was measured for liver function test, and blood creatinine (CREA) and urea level (UREA) were measured for kidney function test.

According to the results, there was a significant difference in body weight measures between only AFB1-induced group (decreasing values) and the other two groups (control and LcS supplemented groups) from day nine till the end of the study (day 20), however, there was no significant difference between the body weight of control group and LcS group.

Induction of aflatoxicosis (group 2) elevated the liver and kidney enzymes. However, with the supplementation of probiotic, a significant reduction in liver enzymes and slight reduction in kidney enzymes were observed. The mean reduction percentage of ALT and AST is 1.43 and 1.25%, respectively, while the mean percentage reduction in CREA and UREA is 1.04 and 1.03%, respectively.

AFB1 was detected from all serum samples except for serum samples in control group. As results indicated, the mean of AFB1 level in the group induced with AFB1 only (1.38 ± 0.55 ppb/mL) was significantly higher ($p < 0.05$) than the group treated with LcS (0.50 ± 0.15 ppb/mL) and there was no significant difference between the control group and the group treated with probiotic.

The authors concluded that LcS supplementation has beneficial effects on rats' body weight, plasma biochemical parameters and AFB1 reduction in blood serum during chronic AFB1 exposure of rats.

The aims of the study of Jebali et al. (2015) [53] were to evaluate the AFB1 and AFM1-binding/degrading ability of *Lactobacillus plantarum* MON03 (isolated from Tunisian artisanal butter) for biological detoxification, to examine its ability to degrade AFB1 and AFM1 in liquid medium, and to evaluate its potential for in vivo preventative effects against AFB1- and AFM1-induced immunomodulation in mice.

In the in vitro studies, *L. plantarum* showed a significant binding ability to AFB1 and AFM1 in phosphate-buffered saline (PBS). Viable *L. plantarum* adsorbed 54.3 ± 7.3% of AFB1 after 12 h and the binding amount rose to 82.3 ± 8.3% after 24 h of incubation in PBS. Regarding AFM1, live *L. plantarum* adsorbed 64.5 ± 5.2% of AFB1 after 12 h and reached 89.1 ± 6.2% by 24 h incubation in PBS. With heated
L. plantarum cells, AFB1 and AFM1 binding decreased to 39.8 ± 0.4% and 52.2 ± 0.5% at 24

h, respectively. *L. plantarum* was able to tolerate gastric acidity (10^6 CFU/mL at pH 3 and 2) and had strongly hydrophilic cell surface properties and adhered Caco-2 cells *in vitro*.

In the in vivo studies, Balb/c mice (10-weeks-of-age, female) were distributed into six treatment groups (10 mice/group) and treated orally (by oral gavage) for 15 days as follows: (1) untreated control, (2) treated with *L. plantarum* (2×10^9 CFU/kg bw), (3) treated with AFB1 (0.25 mg/kg bw) or AFM1 (0.27 mg/kg), and (4) treated with *L. plantarum* +AFB1 or *L. plantarum* +AFM1.

The examined parameters in the study were body weight, lymphoid organ indexes, thymus and spleen cellularity (splenocyte and thymocyte count), hepatologic parameters, histopathological parameters and inflammatory cytokine mRNA expression.

Treatments with AFB1 and AFM1 led to significantly decreased body weight gains, histopathological changes, and decrements in all hematologic (WBC, HCT, HGB, PLT, RBC levels) and immune parameters assessed. According to the results, generally mice receiving AFB1 + *L. plantarum* or AFM1 + *L. plantarum* co-treatment displayed no significant differences in the assayed parameters as compared to the control mice. There were improvements of 37.3 and 49.7% for splenocyte and thymocyte counts relative to those seen in mice in the corresponding AFB1 or AFM1-alone groups. The thymus of each mouse treated with *L. plantarum* alone or with *L. plantarum* with AFB1 or AFM1 had normal histologic appearances in contrast to AFB1 and AFM1 treated samples.

AFB1 and AFM1 exposure during treatment resulted in significantly decreased mRNA expression of IFNγ and TNFα and increased expression level of IL-4 in the spleen. This indicated that AFB1 and AFM1 could have a direct impact on inflammatory cytokine expression. Treatments of AFB1+ *L. plantarum* and AFM1+ *L. plantarum* ameliorated the adverse effects on mRNA expression levels of IFNγ, TNFα, and IL-4.2.

The authors concluded that AFB1 and AFM1 were immunotoxic in Balb/c mice and that addition of *L. plantarum* bacteria to the AFB1 and AFM1 treatment regimen succeeded in ameliorating/preventing the toxicities caused by both AF. In addition, the *L. plantarum* was able to remove highly AFB1 and AFM1 from solution. Its general safeness makes *L. plantarum* a possible candidate for biotechnological development for mycotoxin detoxification.

In the study of Zhang et al. (2019) [54], the effects of *Lactobacillus rhamnosus* GG (ATCC 53013) was evaluated on growth performance and hepatotoxicity in calves fed a single dose of aflatoxin B1 (AFB1). Absorption, distribution, and elimination of AFB1 and AFM1 in rumen fluid, blood, and excretions was also evaluated. Twenty-four male Holstein calves were blocked for BW and age before being randomly assigned to 1 of 3 treatment groups (8 calves per group): (1) untreated control (CON), (2) treated with 4.80 mg of AFB1 (AFB1 only), or (3) treated with 1×10^{10} CFU of *L. rhamnosus* GG suspension and 4.80 mg of AFB1 (AFB1 plus *L. rhamnosus* GG). The amount of AFB1 administered corresponded to approximately 0.038 mg/kg of BW. The calves received a suspension of *L. rhamnosus* GG in 50 mL of PBS daily via oral administration for 14 d before and on the day when they received a single oral dose of AFB1. Body weight was recorded at the beginning of the study (before LGG administration), at the day of AFB1 administration, and at the end of the trial. Rumen fluid, blood, urine, and feces samples were collected continuously for 96 h after AFB1 administration. Average daily gain (ADG) and plasma biochemical parameters were analyzed, and concentrations of AFB1 and AFM1 in the samples were determined for monitoring excretion pattern and toxicokinetics.

According to the results, ADG decreased in AFB1-treated animals and *L. rhamnosus* GG administration mitigated the decrease (0.85 ± 0.08 vs. 0.76 ± 0.18 kg of gain/d). AFB1 treatment increased plasma aspartate aminotransferase, alkaline phosphatase, and lactate dehydrogenase levels. *L. rhamnosus* GG administration was able to mitigate the increase in plasma enzymes activity.

Administration of *L. rhamnosus* GG reduced the fluctuation in plasma enzyme activity induced by AFB1, with a significant effect on AST and LDH (both $p < 0.05$) compared with the AFB1 only calves. These findings indicated that the probiotic treatment increased AFB1 retention within the gastrointestinal tract and may consequently reduce the toxic effects of a single dose of AFB1 in calves. No significant differences were observed for total protein, albumin, globulin, ALT, or triglyceride levels ($p > 0.05$), suggesting that the concentration of AFB1 administered in this trial had only low and transient negative effects on hepatocytes.

The excretion patterns were regular in the study, toxins rapidly appeared in all samples after a single oral dose of AFB1 administration and the peak was sequentially reached in all samples except AFM1 in rumen fluid, the clearance pattern from the peak of concentration was well fitted by exponential decreasing function. Administration of *L. rhamnosus* GG decreased the concentrations of free AFB1 and AFM1 in rumen fluid and reduced the release of toxins into plasma and urine. There was a general reduction in toxokinetic parameters by *L. rhamnosus* GG administration, however, the time of maximum concentration and the terminal half-life showed no significant reduction. Maximum concentration (C_{max}) and area under the time-concentration curve ($AUC_{0\text{-}last}$) parameters represent indirectly the amount of toxin. Administration of *L. rhamnosus* GG reduced C_{max} by 44.50 and 40.14% for AFB1 and AFM1 compared with the AFB1 only treatment calves, respectively; for $AUC_{0\text{-}last}$, the reduction was 44.34 and 43.31%, respectively.

The authors concluded that the absorption, distribution, and excretion of AFB1 and AFM1 were rapid in calves fed a single dose of AFB1. Urine was the main route for the excretion of AFM1. Administration of *L. rhamnosus* GG reduced the absorption of AFB1 in the gastrointestinal tract by increasing the excretion via the feces, thus alleviating the hepatotoxic effect of AFB1.

The study of Ben Salah-Abbés et. al. (2015) [55] is somewhat similar to the study of Jebali et al. (2015) [53] described above regarding research objectives and experiments. The authors of the two studies and the publication timeframes are overlapping. The aim of this study is to investigate the AFM1-binding/degrading microorganism *Lactobacillus plantarum* MON03 isolated from Tunisian artisanal butter for biologic detoxification, to examine its ability to degrade AFM1 in liquid medium, and to evaluate its potential for in vivo preventative effects against AFM1-induced immunotoxicity and genotoxicity in mice. AFB1 is not examined in this study.

In the in vitro experiments, *L. plantarum* tolerance to acid and bile salts, and binding to Caco-2 cells and binding of AFM1 was studied. According to the screening results for tolerance to low pH and bile salts, *L. plantarum* was found to be able to survive (at levels of 10^6 CFU/mL) at pH 2 or under conditions of 1% bile salt. The *L. plantarum* expressed strong adhesive ability to the Caco-2 cells as compared to that by the control *L. rhamnosus GG*. It was found that the AFM1-binding by *L. plantarum* increased in a time-dependent manner. Live *L. plantarum* adsorbed 67.1% (± 6.2%) of AFM1 provided after 12 h, and the binding rose to 93.1 % (± 5.2%) by 24 h of incubation. With the heat-killed *L. plantarum*, AFM1-binding decreased to 55.1% (± 4.5%) after 24 h of incubation in PBS. Note that, in the study of Jebali et al. (2015) [53] bile salt tolerance has not been investigated.

In the in vivo study, Balb/c mice were used and divided into eight treatment groups (n = 10 mice/group) and treated orally (by gavage) daily for 15 days as follows: (1) untreated controls; (2) *L. plantarum* (10^9 CFU/L, ~1 mg/kg body weight) alone; (3) AFM1 only (100 mg/kg); (4) *L. plantarum* (10^9 CFU/kg) + AFM1 (100 mg/kg body weight); (5) a single dose of colchicine (4 mg/kg; positive control for induction of micronuclei); or (6) a single dose of mitomycin C (1 mg/kg; positive control group for chromosomal aberration induction). The colchicine and mitomycin C treatments of the specified mice were given by gavage on d 13 of the treatment protocol.

The results showed that, compared to in control mice, AFM1 treatment led to significantly decreased body weight gains, and caused cytotoxic/genotoxic effects as

indicated by increases in frequencies of polychromatic erythrocytes, as well as those with micronucleation (PCEMN) and chromosomal aberrations, among bone marrow cells. The concurrent administration of *L. plantarum* with AFM1 strongly reduced the adverse effects of AFM1 on each parameter. Mice receiving AFM1 + *L. plantarum* co-treatment displayed no significant differences in the assayed parameters as compared to the control mice.

The *L. plantarum* alone led to an increase in the absolute weights of the thymus of ~20.5%, and AFM1 alone to an increase of ~25.0%. With respect to the spleen, *L. plantarum* alone led to an increase in absolute organ weight of 8.8% and AFM1-alone to an increase of ~14.0%. Co-treatment with *L. plantarum* + AFM1 resulted in an increase in weight of 13.2%. Regarding the splenocyte and thymocyte counts, changes represented improvements of 34.5 and 53.9% in AFM1 + *L. plantarum* co-treatment groups relative to those seen in the mice in the AFM1-alone group.

According to the results of the bone marrow micronucleus assay, mice treated simultaneously with AFM1 and *L. plantarum* showed a significant decrease in the elevation of PCEMN number (now 7.1 [± 0.3]; reduction = 97.4%) that had occurred from the AFM1 treatment, although these values were still higher than in the control mice.

Regarding in vivo chromosomal aberrations, *L. plantarum* + AFM1 co-treatment resulted in significant decreases in total aberrations induced by AFM1 (now 6.6 [± 0.8]/300 metaphase cells counted/mouse; reduction = 96.2%).

By itself, the bacteria caused no adverse effects.

The authors concluded that based on the data, the test bacteria could potentially be beneficial in the detoxification of AFM1-contaminated foods and feeds for humans and animals.

5.4. Other microbes

The goal of the study of Intanoo et al. (2018) [56] was to obtain isolates from ruminal fluid of dairy cattle that can be cultivated under normal conditions for the purpose of biomass production and could survive the conditions in animal stomach for the purpose of AFs detoxification. Isolation was done in 2 steps: in an anaerobic condition followed by an aerobic condition using media with AFB1 addition at 200 ng/mL. AFB1. The top 5-5 isolates of yeast and bacteria showing the least effect from the high dosage of AFB1 were chosen for further assessment. Their abilities to detoxify AFB1 were reported as clear zone size from the diffusion assay. In general, yeast isolates showed a superior ability to detoxify and withstand AFB1, especially at high AFB1 concentrations. At a low AFB1 concentration (200 ng/mL), all isolates performed similarly. A significant drop in the performance of the bacteria was observed when the AFB1 concentration was 600 ng/mL and above. The performance of the yeast remained relatively constant, though statistically different, for up to 1000 ng/mL AFB1.

According to the results of aflatoxin detoxifying efficiency assessed in liquid media containing 200 ng/mL of AFB1, generally, yeast had a superior AFB1 detoxification rate. Most of the AFB1 was degraded by yeasts within 12 h of incubation. All yeast isolates performed similarly in detoxifying AFB1 with detoxification percentages around 89–93%. Bacterial detoxification showed a slower rate, AFB1 decreased continuously over the 24 h period. The percentage of detoxification by bacteria in liquid media ranged from 60% to 82%.

According to the results of the in vitro study, where rumen fluid and TMR diets were used instead of liquid media, bacteria isolates showed faster detoxification rates than of liquid medium, however, the detoxification percentages were similar. Top performers for bacteria isolates in the in vitro system were isolates that showed a 78% and 81% detoxification. AFB1 detoxification rates by yeast isolates in the in vitro system were similar to those in the liquid medium (86–89% detoxification).

According to the identification studies, the best yeast isolates were identified as *Kluyveromyces marxianus* and *Pichia kudriavzevii*. The authors acknowledged that this present study was the first to report the ability of *K. marxianus* CPY1 and RSY5 in AFB1

detoxification. The best bacteria isolates were identified as *Enterococcus faecium*, *Corynebacterium phoceense* and *C. vitaeruminis*.

A preliminary assessment on the biomass production of all isolates has been conducted. It indicated that the biomasses of the isolates could be produced in bulk for their potential use as feed supplement for dairy cattle. The authors concluded that live cell supplements could be an alternative approach for improving mycotoxins detoxifying of microbial in cattle's rumen and improve the milk quality.

In the study of Wang et al. (2018) [57], the microbial consortium TADC7 was domesticated by co-culturing with 500 mg/L AFB1 and 500 mg/L zearalenone (ZEA), to intensify and stabilize its mycotoxin degradation ability. Applying the derived microbial consortium (TMDC), the simultaneous degradation of AFB1 and ZEA was investigated. The influence of the two mycotoxins on consortium composition was also analysed by 16S rRNA sequencing.

TMDC cultured with 500 mg/L AFB1 and 500 mg/L ZEA for 168 h was analysed by LC-MS/MS. Dramatic declines of both AFB1 and ZEA were observed after 168 h of cultivation, indicating that TMDC could simultaneously degrade AFB1 and ZEA effectively.

Degradation of 2000 mg/L AFB1, 2000mg/L ZEA, or their combination by TMDC was determined separately. Degradation mainly occurred within 72 h for both AFB1 and ZEA. The simultaneous addition of AFB1 and ZEA resulted in a slower AFB1 degradation than that observed for AFB1 alone (85.2% degradation at 48 h and 90.1% degradation at 72 h vs. 88.1% degradation at 48 h and 94.7% degradation at 72 h; $p < 0.001$), whereas their final AFB1 degradation ratios were similar (98.9% degradation vs. 99.0% degradation at 168 h; $p = 0.718$). ZEA degradation was more rapid in the group treated with both AFB1 and ZEA than in the group treated with ZEA alone (75.2% degradation at 48 h and 69.2% degradation at 72 h vs. 69.2% degradation at 48 h and 79.3% degradation at 72 h; $p < 0.001$), whereas the final ZEA residual amounts were similar at 168 h (88.5% vs. 88.4%; $p = 0.867$).

For the examination of degradation of mycotoxins by different components of the TMDC culture, after culture in PCS medium without mycotoxins for 48 h, the cell-free supernatants, cell pellets, and intracellular extracts of TMDC were prepared and then cultured with 5000 mg/L AFB1 and 5000 mg/L ZEA in the dark at 55°C for 72 h. After 72 h of cultivation, the cell-free supernatants of TMDC degraded 93.8% of AFB1 and 90.3% of ZEA, and the intracellular extracts degraded 54.3% of AFB1 and 42.1% of ZEA. According to the authors, this indicates that supernatants of TMDC play a dominant role in mycotoxin degradation by the microbial consortium. In contrast, only 27.4% of AFB1 and 25.1% of ZEA were degraded by the viable cells in 72 h, which indicates that there was a little contribution of adsorption to mycotoxin decontamination by TMDC. TMDC cell-free supernatants obtained from TMDC cultured in PCS medium without mycotoxins for 24 h, 48 h, or 72 h were treated with 5000 mg/L AFB1, 5000 mg/L ZEA, or their combination for an extra 0, 4, 8, 24, 48, and 72 h. The TMDC supernatants obtained from the 48-h mycotoxin-free culture showed the maximum degradation rate, i.e., 92.5% for treatment with AFB1 alone, 91.1% for treatment with ZEA alone, and 93.9% AFB1 and 90.3% ZEA for the simultaneous addition of AFB1 plus ZEA ($p < 0.05$). The mycotoxin dynamics showed that the degradation of both AFB1 and ZEA by TMDC supernatants mainly occurred within 48 h.

According to the results of examining microbial diversity dynamics of TMDC during mycotoxin degradation (16S rRNA sequencing), the initial TMDC mainly consisted of the genera *Geobacillus* (12.30%), *Tepidimicrobium* (36.65%), *Clostridium* III (21.20%), *Aeribacillus* (8.84%), *Cellulosibacter* (5.10%), *Desulfotomaculum* (6.44%), and *Tepidanaerobacter* (3.14%). The dominant genera during the rapid mycotoxin degradation stage (24 h) were *Geobacillus* (48.72% for AFB1, 50.00% for ZEA, and 57.39% for AFB1 plus ZEA) and *Tepidimicrobium* (26.61% for AFB1, 29.92% for ZEA, and 22.38% for AFB1 plus ZEA). These findings imply that these two genera might play important roles in AFB1 and ZEA degradation by TMDC.

The authors concluded that their results would facilitate the practical application of microbial consortia in mycotoxin degradation.

In the study of Wang et al. (2019) [58], an AFB1 degrading bacteria *Escherichia coli* CG1061 was isolated from chicken cecum. It exhibited a 93.7% AFB1 degradation rate (HPLC analysis). According to 16S rRNA gene sequence analysis and a multiplex PCR experiment, the bacteria was a non-pathogenic *Escherichia coli*.

The culture supernatant of the 48 h culture of *E. coli* showed an 61.8% degradation rate for AFB1, whereas only 17.6% degradation rate was detected for the intracellular extracts, demonstrating that the active component was constitutively secreted into the extracellular space. When the culture supernatant was treated with proteinase K, a significant decrease in degradation rate from 61.8 to 37.5% occurred. The authors assumed that the active components may be heat-resistant proteins, because after heating of 100°C for 20 min, the degradation rate was 51.3%.

Culture supernatant of *E. coli* was incubated with AFB1 for 72 h, and AFB1 degradation rates were determined in every 12 h (HPLC). In the first 12 h, AFB1 degradation rates increased rapidly from 0 to 63.5%, then reach to 79.0% at 24 h, 86.3% at 48 h, and 91.9% at 72 h. According to the results, culture supernatant of *E. coli* could significantly reduce AFB1 content in first 24 h (reduced nearly 80%). According to the results of examining the effect of incubation pH, the degradation rates of alkaline conditions were higher than that of acid conditions. The highest degradation rate was 44.8% at pH 8.5. Over a broad temperature range from 37 to 70°C, the degradation rates were more than 50%. The highest degradation activity appeared at 55°C (degradation rate, 63.5%). The culture supernatant did not lose its degradation activity at 70°C, indicating that the active components involved were thermostable.

Regarding the results of analysis of degraded AFB1 products, AFB1 was bio-transformed to the product *E. coli* and other metabolites. Based on the results of in vitro experiments on chicken hepatocellular carcinoma (LMH) cells and in vivo experiments on mice, the authors concluded that *E. coli*-degraded AFB1, products by *E. coli* CG1061, are less toxic than the standard AFB1.

The authors concluded that as the isolated bacteria derives from chicken cecum, it is likely to be able to colonize animal gut, which, together with the results of the characterisation make it a possible candidate for the purpose detoxification of AFB1 in food and feed industry.

In the paper of Prettl et al. (2017) [59] aflatoxin degradation efficiency of *Rhodococcus pyridinivorans* K408 strain has been evaluated for detoxification of animal feed sourcing from bioethanol production as a by-product, namely dried distillers grain with solubles (DDGS). In the first experiment, the strain has been added as bacterial inoculum and as bacterial extract in both the liquid and the solid phases of an *Aspergillus flavus* infected bioethanol by-product (i.e., corn-based whole stillage) during a three-day incubation period. AFB1 mobility from the solid phase of the whole stillage was tested by washing it with a phosphate buffer. According to the results, washing removed only small amounts of the toxin, therefore the so-called pre-treated stillage had almost the same AFB1 amounts than the original. The separation into two phases in order to measure in which phase and in which form the bacterium performs better in toxin reduction. Highest level of toxin reduction in the thin stillage was found when *R. pyridinivorans* as inoculum was added to the degradable liquid (94.5% reduction, compared to the initial concentration, however, the difference was not significant compared to *R. pyridinivorans* extract treatment. The main AFB1 content of the whole stillage derived from the solid phase. In the solid phase of the whole stillage the degrading agents did not show any activity in reducing the toxin concentration.

Because of the lack of toxin degradation in the three-day experiment, a longer, 12-day long experiment has also been conducted with only *R. pyridinivorans* inoculum.

According to the results, the aflatoxin content of the stillage significantly decreased between the 3rd and 7th days of the microbial reaction. By the twelfth day, the AFB1

degradation rate of *R. pyridinivorans* strain, in the solid phase, reached 63%. Despite the lower speed the degradation performance appeared to be 75%, which is greater by 12% than in the solid phase.

The growth of bacteria was also monitored. According to the results, the exponential stage of biomass growth changed to a stagnant state after seven days incubation time, in harmony with the mycotoxin degradation rate.

The authors demonstrated the AFB1 degrading characteristics of *R. pyridinivorans* strain in thin and whole stillage phases of bioethanol production by-product and concluded that more optimization is needed to improve bacterial viability and to be able to reduce aflatoxin B1 concentration below the regulatory limit.

In the study of Xu et al. (2017) [60], 43 bacterial isolates collected from maize, rice, and soil samples were tested for aflatoxin-reducing activity. All of them were able to reduce AFB1 to varying degrees in nutrient broth (NB) after incubation for 72 h at 37°C. The highest activity (71.7%) was detected in isolate *Bacillus shackletonii* L7, which was identified as *B. shackletonii* strain LMG 18435 according to a 16S rRNA gene sequence analysis. This is the first report of a bacterium of this genus exhibiting mycotoxin-degrading ability. Degradation efficiency of *B. shackletonii* against various aflatoxins was evaluated and a thermostable enzyme named *Bacillus* aflatoxin-degrading enzyme (BADE) responsible for AFB1 degradation activity was purified and characterized. Genotoxicity of AFB1 degradation products treated with proteins from strain *B. shackletonii* was also analysed.

According to the results, *B. shackletonii* reduced AFB1, AFB2, and AFM1 levels by 92.1%, 84.1%, and 90.4%, respectively, after 72 h at 37°C. The culture supernatant of isolate *B. shackletonii* was more effective than viable cells and cell extracts in degrading higher concentrations of AFB1 after 72 h (77.9% vs. 28.6% and 17.2%, respectively; $p < 0.05$). The AFB1-degrading ability of the supernatant declined to 52.6% and 15.3% upon treatment with proteinase K without and with sodium dodecyl sulphate (SDS), respectively. According to the authors, these results suggest that proteins/enzymes secreted by *B. shackletonii* are involved in AFB1 degradation. A thermostable enzyme, *Bacillus* aflatoxin-degrading enzyme (BADE), the major protein involved in AFB1 detoxification was purified from the boiled supernatant. It has an estimated molecular mass of 22 kDa. According to the results, AFB1-degrading activity is a function of temperature, its optimal value is 70°C (with 47.51% degradation rate measured). The degradation ratio of AFB1 treated with BADE increased following the pH increase, and the degradation rate at pH 8.0 was the highest (31.18%). It was enhanced by Cu^{2+} and inhibited by Zn^{2+}, Mn^{2+}, Mg^{2+} and Li^{+}.

The authors concluded that BADE from *B. shackletonii* L7 is a promising new agent for aflatoxin biodegradation and has potential applications in the detoxification of aflatoxins during food and feed processing. Cloning and expression of the BADE-encoding gene is underway.

5.5. Extracellular enzymes of Basidiomycota

In the study of Scarpari et al. (2014) [61], different uses of white rot basidiomycetous fungus *Trametes versicolor* are proposed as cost-effective methods for the reduction of aflatoxin contamination in maize kernel at large scale.

In the first experiment, inhibition of AB1 in liquid culture of *A. flavus* by liquid filtrate (LF, grown on molasses) and lyophilized solid (LS, grown on solid substrate, inoculated on beet pulp (BPTV) of *T. versicolor* CF117 strain was investigated. According to the results, an inhibition of AB1 production, ranging from 70% to 90% ($p < 0.001$), occurred in case of LF treatment. LS was added to a liquid culture of *A. flavus* at 0.1, 0.5 and 1% w/v. The results showed that the LS added at 1% w/v was able to inhibit completely ($p < 0.01$) the production of aflatoxin B1 up to 7 days post inoculation (dpi) and to reduce dramatically fungal growth. LS added at 0.1% and 0.5% w/v, showed a significant percentage of aflatoxin inhibition as well.

In the second experiment, AFB1-inhibition of LS and LF was studied on maize kernels contaminated by *A. flavus*. According to the results, LS treatment inhibited AFB1 formation up to 21 dpi ($p < 0.001$). *No results were shown in the study regarding LF treatment, however, conclusions drawn by the authors regarding this experiment mention AFB1-inhibiting effects of both LS and LF.*

It is concluded by the authors regarding the first two experiments, that the marked inhibition of aflatoxin biosynthesis, demonstrated by LF and LS on both liquid culture and maize seeds, allowed considering its application for possible use on a large scale.

In the third experiment, degradation of AFB1 by lignin degrading enzymes (laccase) of *T. versicolor* CF294 strain culture filtrate has been investigated because of the low substrate specificity and the AFB1 degrading effects (unknown mechanism) of laccase enzyme. The results showed the ability of
T. versicolor CF294 culture filtrate, displaying a laccase activity of 1.8 U/mL, to degrade - under in vitro conditions, up to 70% of aflatoxin B1 after 48 h ($p < 0.01$). Th effects was also investigated under in vivo conditions, where the culture filtrate was sprayed over previously contaminated maize seeds without significantly altering their moisture level. When treating 0.1 kg of *A. flavus*-inoculated maize kernels, with the culture filtrate of CF294 having laccase activity of 3.5 U/mL, the best performance (70% of toxin degradation) was achieved using the culture filtrate added at 2% v/w ($p < 0.01$). At larger scale, 30 kg of naturally contaminated maize seeds and milled maize seeds were treated. According to the results, both treated samples (seeds and milled seeds) showed a significant degradation of AFB1 content after 7 days of the treatment up to 30% for seeds and 43% for milled seeds ($p < 0.01$ and $p < 0.05$ respectively). The authors concluded that the data obtained in vitro (liquid) and in vivo (maize) experiments indicate that culture filtrates of CF294 was able to reduce aflatoxin content significantly.

In the last experiment, the by-products of AFB1 degradation by laccase have been tested for their putative toxicity on two human lymphoma cancer cells such as OCI-AML3 and U937, being AFB1 known for their cytotoxicity against human immune system cells (MTT assays). The results indicated that 150 ng of AFB1 presented a slight, but significant ($p < 0.01$) toxicity on OCI-AML3 and U937 cells compared to the control (buffer). AFB1 by-products originated from laccase treatment did not affect cell viability compared to the control and to the sole AFB1 ($p < 0.05$).

In the study of Das et al. (2014) [62], the effects of initial concentration of AFB1, metal salts and surfactants on AFB1 degradation in contaminated rice straw by white rot basidiomycetous fungus *Pleurotus ostreatus* strains and analysis of degradation products were investigated, because lignolytic extracellular enzymes of the fungus (laccase and manganese peroxidase (MnP)) have been reported to degrade many environmentally hazardous compounds.

The first experiment examined the effect of initial concentration of AFB1 on its degradation. Different concentrations of AFB1 were added to moist rice straw. According to the results, *p. ostreatus* MTCC 142 exhibited highest laccase (1.68 U/ gds) and MnP (2.06 U/gds) activities at 0.5 μg/mL concentration of AFB1. *P. ostreatus* GHBBF10 demonstrated maximum laccase (1.95 U/gds) and MnP (2.84 U/gds) activities at 0.5 μg/mL concentration of AFB1. Highest degradation of AFB1 (89.14 % and 91.76%) was recorded for *P. ostreatus* MTCC 142 and *P. ostreatus* GHBBF10, respectively, at 0.5 μg/mL concentration of AFB1. HPLC analysis indicated that AFB1 degradation potential of *P. ostreatus* GHBBF10 was higher than *P. ostreatus* MTCC 142 for AFB1 concentrations ranging from 0.5 to 5 μg/mL, however, at 10 and 15 μg/mL, degradation was comparatively lower. With an increase in AFB1 concentration, progressive decrease in degradation was encountered. Lowest degradation was observed at toxin concentration of 15 μg/mL.

The second experiment investigated the effect of metal ions on AFB1 degradation of *P. ostreatus* MTCC 142 and *P. ostreatus* GHBBF10. All the inorganic salts in the experiment supported the hyphal growth of the two strains. However, *P. ostreatus* MTCC 142 showed maximum laccase (2.97 U/gds) and MnP (3.28 U/gds) activities in the presence of Cu^{2+} and

Mn^{2+}, respectively. Highest degradation (92.43%) was observed in the presence of Cu^{2+}, compared to the inoculated control (devoid of Cu^{2+}) which showed 75.67% degradation. In the presence of other salts, AFB1 degradation increased in the following order: Zn^{2+} (82.72%) < Mg^{2+} (85.78%) < Mn^{2+} (88.5%). In contrast, *P. ostreatus* GHBBF10 showed maximum laccase activity (2.76 U/gds) in the presence of Zn^{2+}. Highest degradation of AFB1 (92.06%) was also recorded in the presence of Zn^{2+}. However, highest titre of MnP (2.91 U/gds) was observed in the presence of Mn^{2+}. The result indicated that *P. ostreatus* GHBBF10 may tolerate Zn^{2+} better than Cu^{2+}, thereby yielding higher percentage of AFB1 degradation with Zn^{2+}. In the presence of other metal ions, *P. ostreatus* GHBBF10 exhibited degradation in the following order of increase: Mn^{2+} (86.7%) < Mg^{2+} (86.81%) < Cu^{2+} (91.26%).

The third experiment studied the effect of surfactants on AFB1 degradation. According to the results, all the surfactants used in the study demonstrated substrate colonization by the basidiomycete strains, as observed after 15 days of incubation. *P. ostreatus* MTCC 142 showed highest laccase (2.83 U/gds) and MnP (2.61 U/gds) activities in the presence of Triton X-100, whereas, *P. ostreatus* GHBBF10 demonstrated maximum laccase (2.14 U/gds) and MnP (1.96 U/gds) activities in the presence of Tween 80. Highest degradation of AFB1 (93.88%) by *P. ostreatus* MTCC 142 was recorded in the presence of Triton X-100 followed by Tween 80 (93.07%). *P. ostreatus* GHBBF10, on the other hand, showed maximum degradation (91.09%) in the presence of Tween 80.

In summary of the best results of the experiments, highest degradation was recorded for both *P. ostreatus* MTCC 142 (89.14%) and *P. ostreatus* GHBBF10 (91.76%) at 0.5 μg/mL initial concentration of AFB1. Enhanced degradation was noted for *P. ostreatus* MTCC 142 in the presence of Cu^{2+} and Triton X-100, at toxin concentration of 5 μg/mL. *P. ostreatus* GHBBF10 showed highest degradation in the presence of Zn^{2+} and Tween 80.

The authors concluded that the degradation of AFB1 by *P. ostreatus* strains could be an eco-friendly approach to reduce the level of this mycotoxin in animal feeds. The efficiency of AFB1 degradation could be improved by incorporation of salts of Cu^{2+} and Zn^{2+}, and surfactants like Triton X-100 and Tween 80.

In the study of Branà et al. (2017) [63], the capability of the white-rot and edible fungus *Plerotus eryngii* (king oyster mushroom) to degrade AFB1 both in vitro and in a laboratory-scale mushroom cultivation, using a substrate similar to that routinely used in mushroom farms was investigated. Translocation of AFB1 and aflatoxicol (AFOL) through the thallus to the basidiocarps (fruit bodies) was also examined.

When studying the inhibitory effect of AFB1 on growth of *P. eryngii*, the results indicated that the growth on solid medium (malt extractagar, MEA) was significantly reduced at concentrations of AFB1 500 ng/mL or higher. However, the addition of 5% wheat straw to the culture medium increased the tolerance of *P. eryngii* to AFB1 and no inhibition was observed at a AFB1 content of 500 ng/mL; degradation of AFB1 in MEA supplemented with 5% wheat straw and 2.5% (w/v) maize flour was 71 ± 94% after 30 days of growth.

According to the results of examining the degradation of AFB1 in liquid medium by *P. eryngii*, after 10 days of growth the isolates ITEM 13730, ITEM 17015 and ITEM 13662 degraded 99% of AFB1. The isolates ITEM 13682 and ITEM 13696 degraded AFB1 by 95% and 94%, respectively, and the isolates ITEM 13681, ITEM 13697 and ITEM 13676 showed approximately 90% degradation. The isolate that showed the lowest degradative capability (80%) was ITEM 13688. However, after 20 days of incubation the differences among the strains were not statistically significant and after 30 days all the isolates totally degraded AFB1. In the experiment, where degradation of AFB1 by the *P. eryngii* isolates grown on the agar medium (MEASM) was analysed, the results show that after 15 days of incubation, all the strains significantly degraded the AFB1 supplemented to the medium, in percentages that ranged from 43 to 59%. After 30 days the degradation of AFB1 by the strain ITEM 17015 increased to 84%, and that of the strains ITEM 13696 and ITEM 13662 were 83 and 82% respectively. The strains ITEM 13688 and ITEM 13676, the least

effective strains in degradation of AFB1, degraded AFB1 in the medium by 71 and 65% respectively.

Further, AFB1 degradation by *P. eryngii* strain ITEM 13681 was tested in a laboratory-scale mushroom cultivation. The mushroom growth medium contained 25% (w/w) of maize spiked with AFB1 to the final content of 128 μg/kg. According to the results, 7 d degradation (after inoculation) was 13 ± 6% and at day 28 the level of degradation reached the maximum value of 86 ± 0.2% that did not change significantly until the end of the experiment (42 d). There was no significant reduction of either biological efficiency or mushroom yield (72 ± 11% and 9 ± 1 g, compared to the control group with values of 70 ± 5% and 9 ± 0,8 g, respectively). Neither the biomass produced on the mushroom substrate nor the mature basidiocarps contained detectable levels of AFB1 or its metabolite aflatoxicol, thus ruling out the translocation of these toxins through the fungal thallus.

The authors concluded that these findings contribute towards the development of a novel technology for remediation of AFB1-contaminated corn through the exploitation of the degradative capability of *P. eryngii* and its bioconversion into high nutritional value material intended for feed production.

References

1. Chiewchan, N.; Mujumdar, A.S.; Devahastin, S. Application of Drying Technology to Control Aflatoxins in Foods and Feeds: A Review. *Dry. Technol.* **2015**, *33*, 1700–1707, https://doi.org/10.1080/07373937.2015.1068795.
2. Ismail, A.; Gonçalves, B.L.; de Neeff, D.V.; Ponzilacqua, B.; Coppa, C.C.; Hintzsche, H.; Sajid, M.; da Cruz, A.G.; Corassin, C.H.; Oliveira, C.A. Aflatoxin in foodstuffs: Occurrence and recent advances in decontamination. *Food Res. Int.* **2018**, *113*, 74–85, https://doi.org/10.1016/j.foodres.2018.06.067.
3. Koc, F.; Kara, S. Environmental Factors Affecting Efficacy of Some Essential Oils and Potassium Sorbate to Control Growth of Aspergillus Flavus, Aspergillus Parasiticus on Wheat and Maize Grains. *J. Agric. Sci. Technol.* **2014**, *16*, 1325–1334.
4. Garcia, D.; Ramos, A.J.; Sanchis, V.; Marin, S. Equisetum arvense hydro-alcoholic extract: phenolic composition and antifungal and antimycotoxigenic effect against Aspergillus flavus and Fusarium verticillioides in stored maize. *J. Sci. Food Agric.* **2012**, *93*, 2248–2253, https://doi.org/10.1002/jsfa.6033.
5. Shi, H.; Li, S.; Bai, Y.; Prates, L.L.; Lei, Y.; Yu, P. Mycotoxin contamination of food and feed in China: Occurrence, detection techniques, toxicological effects and advances in mitigation technologies. *Food Control* **2018**, *91*, 202–215, https://doi.org/10.1016/j.foodcont.2018.03.036.
6. Asghar, M.A.; Zahir, E.; Shahid, S.M.; Khan, M.N.; Iqbal, J.; Walker, G. Iron, copper and silver nanoparticles: Green synthesis using green and black tea leaves extracts and evaluation of antibacterial, antifungal and aflatoxin B1 adsorption activity. *LWT* **2018**, *90*, 98–107, https://doi.org/10.1016/j.lwt.2017.12.009.
7. Aiko, V.; Edamana, P.; Mehta, A. Decomposition and detoxification of aflatoxin B1by lactic acid. *J. Sci. Food Agric.* **2015**, *96*, 1959–1966, https://doi.org/10.1002/jsfa.7304.
8. Singh, R.; Mandal, A.B. Efficacy of Fumaric and Citric Acids in Preventing Biosynthesis of Aflatoxins in Poultry Feed with Variable Moisture Content. Indian *J. Anim. Sci.* **2014**, *84*, 453–456.
9. Alam, S.; Shah, H.U.; Khan, N.A.; Zeb, A.; Shah, A.S.; Magan, N. Water availability and calcium propionate affect fungal population and aflatoxins production in broiler finisher feed during storage. *Food Addit. Contam. Part A* **2014**, *31*, 1896–1903, https://doi.org/10.1080/19440049.2014.963699.
10. Lee, J.; Her, J.-Y.; Lee, K.-G. Reduction of aflatoxins (B1, B2, G1, and G2) in soybean-based model systems. *Food Chem.* **2015**, *189*, 45–51, https://doi.org/10.1016/j.foodchem.2015.02.013.
11. Alam, S.S.; Deng, Y.; Dixon, J.B. Minimal interference of glucose and ethanol on aflatoxin B1 adsorption by smectites. *Appl. Clay Sci.* **2015**, *104*, 143–149, https://doi.org/10.1016/j.clay.2014.11.022.

12. Antonelo, D.; Lancaster, N.A.; Melnichenko, S.; Muegge, C.R.; Schoonmaker, J.P. Effects of clay on toxin binding capacity, ruminal fermentation, diet digestibility, and growth of steers fed high-concentrate diets.. *J. Anim. Sci.* **2017**, *95*, 4658–4667, https://doi.org/10.2527/jas2017.1473.
13. Joannis-Cassan, C.; Tozlovanu, M.; Hadjeba-Medjdoub, K.; Ballet, N.; Pfohl-Leszkowicz, A. Binding of Zearalenone, Aflatoxin B1, and Ochratoxin A by Yeast-Based Products: A Method for Quantification of Adsorption Performance. *J. Food Prot.* **2011**, *74*, 1175–1185, https://doi.org/10.4315/0362-028x.jfp-11-023.
14. Fraser, C.M. Merck Veterinary Manual; 7th ed.; Merck and Company, Inc: Rahway, NJ., 1991
15. Maki, C.; Monteiro, A.; Elmore, S.; Tao, S.; Bernard, J.; Harvey, R.; Romoser, A.; Phillips, T. Calcium montmorillonite clay in dairy feed reduces aflatoxin concentrations in milk without interfering with milk quality, composition or yield. *Anim. Feed Sci. Technol.* **2016**, *214*, 130–135, https://doi.org/10.1016/j.anifeedsci.2016.02.014.
16. Maki, C.; Thomas, A.; Elmore, S.; Romoser, A.; Harvey, R.; Ramirez, H.A.R.; Phillips, T. Effects of calcium montmorillonite clay and aflatoxin exposure on dry matter intake, milk production, and milk composition. *J. Dairy Sci.* **2016**, *99*, 1039–1046, https://doi.org/10.3168/jds.2015-10242.
17. Pate, R.; Compart, D.P.; Cardoso, F. Aluminosilicate clay improves production responses and reduces inflammation during an aflatoxin challenge in lactating Holstein cows. *J. Dairy Sci.* **2018**, *101*, 11421–11434, https://doi.org/10.3168/jds.2018-15024.
18. Sulzberger, S.; Melnichenko, S.; Cardoso, F. Effects of clay after an aflatoxin challenge on aflatoxin clearance, milk production, and metabolism of Holstein cows. *J. Dairy Sci.* **2017**, *100*, 1856–1869, https://doi.org/10.3168/jds.2016-11612.
19. Soufiani, G.N.; Razmara, M.; Kermanshahi, H.; Velázquez, A.B.; Daneshmand, A. Assessment of aflatoxin B 1 adsorption efficacy of natural and processed bentonites: In vitro and in vivo assays. *Appl. Clay Sci.* **2016**, *123*, 129–133, https://doi.org/10.1016/j.clay.2016.01.019.
20. Akhtar, R.; Sardar, M.; Saima, N.; Saleem, G.; Imran, S.; Aslam, A. Responses of Nili-Ravi buffalo to aflatoxin B1 with and without toxin adsorbents. *J. Anim. Feed Sci.* **2014**, *23*, 317–323, https://doi.org/10.22358/jafs/65667/2014.
21. Jiang, Y.-H.; Wang, P.; Yang, H.-J.; Chen, Y. The Efficacy of Bamboo Charcoal in Comparison with Smectite to Reduce the Detrimental Effect of Aflatoxin B1 on In Vitro Rumen Fermentation of a Hay-Rich Feed Mixture. *Toxins* **2014**, *6*, 2008–2023, https://doi.org/10.3390/toxins6072008.
22. Rojo, F.; Patricio Martinez, S.; Isaias Espinoza, V.H.; Nathal Vera, M.A.; De Lucas Palacios, E.; Reyes Velazquez, W.P. Comparison of Methods to Evaluate Aflatoxin B1 Exposure in Dairy Cattle and the Effect of Mycotoxin Adsorbents to Reduce AFM1 Residues in Milk. *Rev. Mex. Cienc.* Pecu. **2014**, *5*, 1–15.
23. Kissell, L.; Davidson, S.; Hopkins, B.A.; Smith, G.W.; Whitlow, L.W. Effect of experimental feed additives on aflatoxin in milk of dairy cows fed aflatoxin-contaminated diets. *J. Anim. Physiol. Anim. Nutr.* **2012**, *97*, 694–700, https://doi.org/10.1111/j.1439-0396.2012.01311.x.
24. Jiang, Y.; Ogunade, I.; Kim, D.; Li, X.; Pech-Cervantes, A.; Arriola, K.; Oliveira, A.; Driver, J.; Ferraretto, L.; Staples, C.; et al. Effect of adding clay with or without a Saccharomyces cerevisiae fermentation product on the health and performance of lactating dairy cows challenged with dietary aflatoxin B1. *J. Dairy Sci.* **2018**, *101*, 3008–3020, https://doi.org/10.3168/jds.2017-13678.
25. Weatherly, M.; Pate, R.; Rottinghaus, G.; Filho, F.R.; Cardoso, F. Physiological responses to a yeast and clay-based adsorbent during an aflatoxin challenge in Holstein cows. *Anim. Feed Sci. Technol.* **2018**, *235*, 147–157, https://doi.org/10.1016/j.anifeedsci.2017.11.019.
26. Ramales-Valderrama, R.A.; Vázquez-Durán, A.; Méndez-Albores, A. Biosorption of B-aflatoxins Using Biomasses Obtained from Formosa Firethorn [Pyracantha koidzumii (Hayata) Rehder]. *Toxins* **2016**, *8*, 218, https://doi.org/10.3390/toxins8070218.
27. Naseer, O.; Khan, J.A.; Khan, M.S.; Omer, M.O.; Anjum, K.M.; Naseer, J.; Sohail, M.L. Study of Prevalence of Different Fungal Species in Calf Feed and Comparative Antifungal Efficacy of Methanolic Extracts of Indigenous Plants against Aspergillus Species. *Buffalo Bull.* **2018**, *37*, 181–190.

28. Antibacterial and Antifungal Survey in Plants used in Indigenous Herbal-Medicine of South East Regions of Iran. *J. Biol. Sci.* **2004**, *4*, 405–412, https://doi.org/10.3923/jbs.2004.405.412.
29. Santoyo, S.; Ramírez-Anguiano, A.C.; Reglero, G.; Soler-Rivas, C. Improvement of the antimicrobial activity of edible mushroom extracts by inhibition of oxidative enzymes. *Int. J. Food Sci. Technol.* **2009**, *44*, 1057–1064, https://doi.org/10.1111/j.1365-2621.2008.01896.x.
30. Fani-Makki, O.; Omidi, A.; Ansari-Nik, H.; Hasheminejad, S.A. In Vitro Assessment of Milk Thistle Seeds as a Natural Anti-Aflatoxin B1. *Acta Veter- Eurasia* **2020**, *44*, 1–5, https://doi.org/10.5152/actavet.2018.002.
31. (Feedap), E.P.O.A.A.P.O.S.U.I.A.F.; Rychen, G.; Aquilina, G.; Azimonti, G.; Bampidis, V.; Bastos, M.D.L.; Bories, G.; Chesson, A.; Cocconcelli, P.S.; Flachowsky, G.; et al. Safety and efficacy of a preparation of algae interspaced bentonite as a feed additive for all animal species. *EFSA J.* **2016**, *14*, https://doi.org/10.2903/j.efsa.2016.4623.
32. Xiong, J.; Wang, Y.; Zhou, H.; Liu, J. Effects of dietary adsorbent on milk aflatoxin M1 content and the health of lactating dairy cows exposed to long-term aflatoxin B1 challenge. *J. Dairy Sci.* **2018**, *101*, 8944–8953, https://doi.org/10.3168/jds.2018-14645.
33. Xiong, J.; Wang, Y.; Nennich, T.; Li, Y.; Liu, J. Transfer of dietary aflatoxin B1 to milk aflatoxin M1 and effect of inclusion of adsorbent in the diet of dairy cows. *J. Dairy Sci.* **2015**, *98*, 2545–2554, https://doi.org/10.3168/jds.2013-7842.
34. Jovaišienė, J.; Bakutis, B.; Baliukonienė, V.; Gerulis, G. Fusarium and Aspergillus mycotoxins effects on dairy cow health, performance and the efficacy of Anti-Mycotoxin Additive. *Pol. J. Veter- Sci.* **2016**, *19*, 79–87, https://doi.org/10.1515/pjvs-2016-0011.
35. Aslam, N.; Rodrigues, I.; McGill, D.M.; Warriach, H.M.; Cowling, A.; Haque, A.; Wynn, P.C. Transfer of aflatoxins from naturally contaminated feed to milk of Nili-Ravi buffaloes fed a mycotoxin binder. *Anim. Prod. Sci.* **2016**, *56*, 1637–1642, https://doi.org/10.1071/an14909.
36. Naveed, S.; Chohan, K.; Jabbar, M.; Ditta, Y.; Ahmed, S.; Ahmad, N.; Akhtar, R. Aflatoxin M1 in Nili-ravi buffaloes and its detoxification using organic and inorganic toxin binders. *J. Hell. Veter- Med Soc.* **2018**, *69*, 873–878, https://doi.org/10.12681/jhvms.16848.
37. Ullah, H.A.; Durrani, A.Z.; Ijaz, M.; Javeed, A.; Sadique, U.; Hassan, Z.U.; Rahman, A.U.; Shah, M.; Khattak, I. Dietary mycotoxins binders: a strategy to reduce aflatoxin m1 residues and improve milk quality of lactating Beetal goats. *J. Consum. Prot. Food Saf.* **2016**, *11*, 305–309, https://doi.org/10.1007/s00003-016-1046-0.
38. Ogunade, I.; Arriola, K.; Jiang, Y.; Driver, J.; Staples, C.; Adesogan, A. Effects of 3 sequestering agents on milk aflatoxin M1 concentration and the performance and immune status of dairy cows fed diets artificially contaminated with aflatoxin B1. *J. Dairy Sci.* **2016**, *99*, 6263–6273, https://doi.org/10.3168/jds.2016-10905.
39. Dogi, C.; Cristofolini, A.; Pereyra, M.G.; García, G.; Fochesato, A.; Merkis, C.; Dalcero, A.; Cavaglieri, L. Aflatoxins and Saccharomyces cerevisiae: yeast modulates the intestinal effect of aflatoxins, while aflatoxin B1 influences yeast ultrastructure. *World Mycotoxin J.* **2017**, *10*, 171–181, https://doi.org/10.3920/wmj2016.2115.
40. Pereyra, M.G.; Dogi, C.; Lisa, A.T.; Wittouck, P.; Ortíz, M.; Escobar, F.; Bagnis, G.; Yaciuk, R.; Poloni, L.; Torres, A.M.; et al. Genotoxicity and cytotoxicity evaluation of probiotic Saccharomyces cerevisiae RC016: a 60-day subchronic oral toxicity study in rats. *J. Appl. Microbiol.* **2014**, *117*, 824–833, https://doi.org/10.1111/jam.12552.
41. Magnoli, A.; Rodriguez, M.; Poloni, V.; Rojo, M.; Combina, M.; Chiacchiera, S.; Dalcero, A.; Cavaglieri, L. Novel yeast isolated from broilers' feedstuff, gut and faeces as aflatoxin B1adsorbents. *J. Appl. Microbiol.* **2016**, *121*, 1766–1776, https://doi.org/10.1111/jam.13297.
42. Poloni, V.; Dogi, C.; Pereyra, C.M.; Juri, M.G.F.; Köhler, P.; Rosa, C.A.R.; Dalcero, A.M.; Cavaglieri, L.R. Potentiation of the effect of a commercial animal feed additive mixed with different probiotic yeast strains on the adsorption of aflatoxin B1. *Food Addit. Contam. Part A* **2015**, *32*, 970–976, https://doi.org/10.1080/19440049.2015.1024761.

43. Gonçalves, J.L.; Rosim, R.; Cappato, L.; da Cruz, A.G.; Oliveira, C.; Corassin, C. Effects of different sources of Saccharomyces cerevisiae biomass on milk production, composition, and aflatoxin M1 excretion in milk from dairy cows fed aflatoxin B1. *J. Dairy Sci.* **2017**, *100*, 5701–5708, https://doi.org/10.3168/jds.2016-12215.
44. A Tayel, A.; El-Tras, W.F.; Moussa, S.; A El-Agamy, M. Antifungal action ofPichia anomalaagainst aflatoxigenicAspergillus flavusand its application as a feed supplement. *J. Sci. Food Agric.* **2013**, *93*, 3259–3263, https://doi.org/10.1002/jsfa.6169.
45. Dogi, C.A.; Pellegrino, M.; Poloni, V.; Poloni, L.; Pereyra, C.M.; Sanabria, A.; Pianzzola, M.J.; Dalcero, A.; Cavaglieri, L. Efficacy of corn silage inoculants on the fermentation quality under farm conditions and their influence on Aspergillus parasitucus, A. flavus and A. fumigatus determined by q-PCR. *Food Addit. Contam. Part A* **2015**, *32*, 229–235, https://doi.org/10.1080/19440049.2014.986223.
46. Zielińska, K.J.; Fabiszewska, A.U. Improvement of the quality of maize grain silage by a synergistic action of selected lactobacilli strains. *World J. Microbiol. Biotechnol.* **2017**, *34*, 9–9, https://doi.org/10.1007/s11274-017-2400-9.
47. Ying, H.L.; Borjigin, N.; Yu, Z. Effect of inoculants and fibrolytic enzymes on the fermentation characteristics,in vitrodigestibility and aflatoxins accumulation of whole-crop corn silage. *Grassl. Sci.* **2017**, *63*, 69–78, https://doi.org/10.1111/grs.12157.
48. Z.X. Gainesville 32608 (host institution) University of Florida Department of Animal Sciences; Amaro, F.; Romero, J.; Pereira, O.; Jeong, K.; Adesogan, A. The capacity of silage inoculant bacteria to bind aflatoxin B1 in vitro and in artificially contaminated corn silage. *J. Dairy Sci.* **2017**, *100*, 7198–7210, https://doi.org/10.3168/jds.2016-12370.
49. Rather, I.A.; Seo, B.J.; Kumar, V.J.R.; Choi, U.-H.; Choi, K.-H.; Lim, J.; Park, Y.-H. Biopreservative potential of Lactobacillus plantarum YML007 and efficacy as a replacement for chemical preservatives in animal feed. *Food Sci. Biotechnol.* **2013**, *23*, 195–200, https://doi.org/10.1007/s10068-014-0026-3.
50. Dogi, C.; Fochesato, A.; Armando, R.; Pribull, B.; de Souza, M.; Coelho, I.D.S.; de Melo, D.A.; Dalcero, A.; Cavaglieri, L. Selection of lactic acid bacteria to promote an efficient silage fermentation capable of inhibiting the activity of Aspergillus parasiticus and Fusarium gramineraum and mycotoxin production. *J. Appl. Microbiol.* **2013**, *114*, 1650–1660, https://doi.org/10.1111/jam.12173.
51. Drobna, E.; Rauova, D.; Majekova, H.; Greif, G.; Mikus, P. Antifungal Activity and Aflatoxin Binding Ability of Lactobacillus Species Isolated from Lamb and Goatling Stomach Mucus. *J. Food Nutr. Res.* **2017**, *56*, 255–264.
52. Nasrabadi, E.N.; Jamaluddin, R.; Mutalib, M.A.; Khaza'Ai, H.; Khalesi, S.; Redzwan, S.M. Reduction of aflatoxin level in aflatoxin-induced rats by the activity of probiotic Lactobacillus casei strain Shirota. *J. Appl. Microbiol.* **2013**, *114*, 1507–1515, https://doi.org/10.1111/jam.12148.
53. Jebali, R.; Abbès, S.; Ben Salah-Abbès, J.; Ben Younes, R.; Haous, Z.; Oueslati, R. Ability ofLactobacillus plantarumMON03 to mitigate aflatoxins (B1and M1) immunotoxicities in mice. *J. Immunotoxicol.* **2014**, *12*, 290–299, https://doi.org/10.3109/1547691x.2014.973622.
54. Zhang, L.; Liu, S.; Zhao, X.; Wang, N.; Jiang, X.; Xin, H.; Zhang, Y. Lactobacillus rhamnosus GG modulates gastrointestinal absorption, excretion patterns, and toxicity in Holstein calves fed a single dose of aflatoxin B1. *J. Dairy Sci.* **2019**, *102*, 1330–1340, https://doi.org/10.3168/jds.2018-15444.
55. Ben Salah-Abbès, J.; Abbès, S.; Jebali, R.; Haous, Z.; Oueslati, R. Potential preventive role of lactic acid bacteria against Aflatoxin M1immunotoxicity and genotoxicity in mice. *J. Immunotoxicol.* **2014**, *12*, 107–114, https://doi.org/10.3109/1547691x.2014.904025.
56. Intanoo, M.; Kongkeitkajorn, M.B.; Pattarajinda, V.; Bernard, J.K.; Callaway, T.R.; Suriyasathaporn, W.; Phasuk, Y. Isolation and screening of aflatoxin-detoxifying yeast and bacteria from ruminal fluids to reduce aflatoxin B1 contamination in dairy cattle feed. *J. Appl. Microbiol.* **2018**, *125*, 1603–1613, https://doi.org/10.1111/jam.14060.
57. Wang, Y.; Zhao, C.; Zhang, D.; Zhao, M.; Zheng, D.; Peng, M.; Cheng, W.; Guo, P.; Cui, Z. Simultaneous degradation of aflatoxin B 1 and zearalenone by a microbial consortium. *Toxicon* **2018**, *146*, 69–76, https://doi.org/10.1016/j.toxicon.2018.04.007.
58. Wang, L.; Wu, J.; Liu, Z.; Shi, Y.; Liu, J.; Xu, X.; Hao, S.; Mu, P.; Deng, F.; Deng, Y. Aflatoxin B1 Degradation and Detoxification by Escherichia coli CG1061 Isolated From Chicken Cecum. *Front. Pharmacol.* **2019**, *9*, https://doi.org/10.3389/fphar.2018.01548.

59. Prettl, Z.; Dési, E.; Lepossa, A.; Kriszt, B.; Kukolya, J.; Nagy, E. Biological degradation of aflatoxin B 1 by a Rhodococcus pyridinivorans strain in by-product of bioethanol. *Anim. Feed Sci. Technol.* **2016**, *224*, 104–114, https://doi.org/10.1016/j.anifeedsci.2016.12.011.
60. Xu, L.; Ahmed, M.F.E.; Sangare, L.; Yueju Zhao; Selvaraj, J.N.; Fuguo Xing; Yan Wang; Hongping Yang; Yang Liu Novel Aflatoxin-Degrading Enzyme from Bacillus Shackletonii L7. *Toxins* **2017**, *9*, 36, https://doi.org/10.3390/toxins9010036.
61. Scarpari, M.; Bello, C.; Pietricola, C.; Zaccaria, M.; Bertocchi, L.; Angelucci, A.; Ricciardi, M.R.; Scala, V.; Parroni, A.; Fabbri, A.A.; et al. Aflatoxin Control in Maize by Trametes versicolor. *Toxins* **2014**, *6*, 3426–3437, https://doi.org/10.3390/toxins6123426.
62. Das, A.; Bhattacharya, S.; Palaniswamy, M.; Angayarkanni, J. Biodegradation of aflatoxin B1 in contaminated rice straw by Pleurotus ostreatus MTCC 142 and Pleurotus ostreatus GHBBF10 in the presence of metal salts and surfactants. *World J. Microbiol. Biotechnol.* **2014**, *30*, 2315–2324, https://doi.org/10.1007/s11274-014-1657-5.
63. Branà, M.T.; Cimmarusti, M.T.; Haidukowski, M.; Logrieco, A.F.; Altomare, C. Bioremediation of aflatoxin B1-contaminated maize by king oyster mushroom (Pleurotus eryngii). *PLoS ONE* **2017**, *12*, e0182574, https://doi.org/10.1371/journal.pone.0182574.
